# Physicochemical Properties and Stability of Antioxidant Peptides from Swim Bladder of Grass Carp (*Ctenopharyngodon idella*)

**DOI:** 10.3390/foods14071216

**Published:** 2025-03-30

**Authors:** Suxin Li, Jinhui Gu, Yiyi Liu, Weiqiang Qiu, Wenzheng Shi

**Affiliations:** 1 College of Food Science and Technology, Shanghai Ocean University, Shanghai 201306, China; sher_rylai@163.com (S.L.); 13861621339@163.com (J.G.); yiiyiliu@163.com (Y.L.); 2 Marine Biomedical Science and Technology Innovation Platform of Lin-gang Special Area, Shanghai 201306, China

**Keywords:** peptide, swim bladder, antioxidative activity, stability, peptide synthesis, digestion simulation

## Abstract

Grass carp swim bladder collagen peptides (GCPs) were purified by ultrafiltration and Sephadex G-15 chromatography to obtain GCP-II, which exhibited superior antioxidant activity. GCP-II exhibited 78.22% ABTS^+^ scavenging activity and 72.91% Fe^2^⁺ chelating activity, along with around 90% thermal stability between 4 °C and 100 °C. Environmental factors such as 4% NaCl reduced superoxide scavenging to 59.16% of the original and 0.2% citric acid reduced it to 71.57% of the original, while glucose showed minimal impact on the antioxidant activity of GCP-II. From 464 GCP-II sequences analyzed via LC-MS/MS, 7 bioactive peptides were selected based on antioxidant activity and functional sequence motifs, and were named peptides 1 to 7, respectively. These peptides were synthesized through solid-phase methods, validated for purity exceeding 95% using HPLC and mass spectrometry, and tested for antioxidant performance. Peptides 1, 3, 6, and 7 demonstrated notable antioxidant efficacy: peptide 6 showed 63.31% ABTS^+^ scavenging activity at 1 mg/mL, while peptides 3 and 6 exhibited synergistic effects in DPPH and hydroxyl radical scavenging experiments, surpassing theoretical values by 0.88% and 2.16%, respectively. This study underscores the potential of synthetic GCPs and GCP-II-derived peptides as functional antioxidants, particularly peptide 3 and peptide 6.

## 1. Introduction

Grass carp, as one of the important freshwater fish species in China, is abundant in swim bladder resources, which were previously often discarded or used as low-value feed [1]. However, with the deepening research on collagen peptides from grass carp swim bladders (GCPs) in recent years, various biological activities such as antioxidant, anti-aging, and wound-healing promotion properties have been discovered [2], providing a scientific basis for their development and application in functional foods, cosmetics, and biomedical fields. The escalating demand for natural antioxidants in the food and pharmaceutical industries has driven extensive research into bioactive peptides derived from underutilized protein sources [3], including marine and aquatic by-products [4].

The integration of antioxidant peptides into functional foods and nutraceuticals is a rapidly growing field [5]. Similarly, marine collagen peptides exhibit synergistic effects with ascorbic acid in anti-aging skincare formulations by neutralizing reactive oxygen species (ROS) and enhancing collagen synthesis [6].

Peptides are particularly advantageous due to their high thermal stability (retaining >90% activity at 100 °C) and resistance to gastrointestinal digestion, making them suitable for baked goods, pasteurized beverages, and enteric-coated supplements [7]. Recent innovations include their encapsulation in chitosan nanoparticles to enhance bioavailability in functional foods [8] and their use as natural preservatives in ready-to-eat seafood [9]. These applications align with global trends toward clean-label ingredients and sustainable utilization of fishery by-products [10,11,12]. Therefore, studying the antioxidant stability of peptides in different food ingredients and temperature environments is of great importance for analyzing their potential as food ingredients with high antioxidant activity [13].

This study aims to explore the preparation process of GCPs through systematic experimental methods, characterize their structural features, evaluate their in vitro antioxidant activity and digestive stability, and further verify the experimental conclusions through synthetic peptides, thereby providing a theoretical basis and technical support for the development and application of GCPs.

## 2. Materials and Methods

### 2.1. Materials

The experimental materials and instrumentation used in this chapter are listed in Table 1. All equipment and materials used in this study were procured from Shanghai, China, unless otherwise specified.

### 2.2. Preparation and Purification of GCPs

Using medium-sized fresh grass carp swim bladders as the primary raw material, GCPs were prepared through an ultrasound-assisted dual-enzyme hydrolysis method [14]. An enzymatic hydrolysis substrate pH of 9, a solid–liquid ratio of 1:10, and an ultrasound frequency of 25 kHz were maintained. Additionally, the total enzyme dosage was set at 1% of the sample weight, the ultrasound power at 145 W, the ultrasound treatment time for 25 min, and the specific ratio of alkaline protease to neutral protease at 0.46%:0.54%. The obtained GCPs were then subjected to ultrafiltration to fractionate into GCPs with molecular weights greater than 100 kDa, 100 kDa to 5 kDa, 5 kDa to 3 kDa, and less than 3 kDa. Among these fractions, the GCPs with a molecular weight of less than 3 kDa were further purified using G-15 dextran gel chromatography. The three fractions obtained from chromatography were named GCP-I, GCP-II, and GCP-III, respectively. All sample solutions mentioned above were freeze-dried and stored at −80 °C for subsequent use [15].

### 2.3. Determination of Amino Acid Composition

To gain a more specific understanding of the types and quantities of all amino acids constituting the active peptides from GCPs, an LA8080 amino acid analyzer was used to conduct amino acid composition analysis of the unpurified GCP enzymatic hydrolysate. The amino acid composition of the freeze-dried powder of GCPs obtained in Section 2.2, prior to purification, was determined with slight modifications to the method described by Shen [16].

### 2.4. Determination of In Vitro Antioxidant Activity of GCPs

GCP fractions of various molecular weights obtained from Section 2.2, along with GCP-I, GCP-II, and GCP-III, were subjected to six in vitro antioxidant activity assays: ABTS radical scavenging assay [17], DPPH radical scavenging assay [18], hydroxyl radical scavenging assay [19], superoxide anion radical scavenging assay [20], total reducing power determination [21], and ferrous ion chelating assay [22]. Based on the comprehensive experimental results, the primary samples for subsequent experiments were selected. To explore the impact of concentration on the antioxidant activity of the samples, the freeze-dried peptide powders of four gradient molecular weights obtained after ultrafiltration were first dissolved in ultrapure water and then adjusted to concentrations of 1 mg/mL, 5 mg/mL, and 10 mg/mL. Antioxidant activity assays were then conducted.

### 2.5. In Vitro Simulated Gastrointestinal Digestion Experiments

A simulated gastrointestinal digestion system was established with minor adjustments based on the method described by Minekus M et al. [23].

#### 2.5.1. In Vitro Simulated Oral Digestion

Firstly, 5.00 g of freeze-dried powder of GCP-I, GCP-II, and GCP-III, obtained through ultrafiltration and chromatography, was separately placed in 50 mL centrifuge tubes. Secondly, 4 mL of Simulated Salivary Fluid (SSF) solution at pH 7 (containing 15.1 mmol/L KCl, 3.7 mmol/L KH_2_PO_4_, 3.6 mmol/L NaHCO_3_, 0.15 mmol/L MgCl_2_(H_2_O)_6_, and 0.06 mmol/L (NH_4_)_2_CO_3_) and 25 μL of CaCl_2_ were added to the centrifuge tubes. The tubes were then oscillated at a constant temperature of 37 °C for 5 min and stored at −80 °C.

#### 2.5.2. In Vitro Simulated Gastric Digestion

Next, 8 mL of Simulated Gastric Fluid (SGF) solution at pH 3 (containing 6.9 mmol/L KCl, 0.9 mmol/L KH_2_PO_4_, 25 mmol/L NaHCO_3_, 47.2 mmol/L NaCl, 0.12 mmol/L MgCl_2_(H_2_O)_6_, and 0.5 mmol/L (NH_4_)_2_CO_3_), 4000 U/mL of pepsin, and 5 μL of CaCl_2_ were added into the samples that had completed oral digestion. The samples were oscillated at a constant temperature of 37 °C for 2 h and stored at −80 °C.

#### 2.5.3. In Vitro Simulated Intestinal Digestion

Then, 16 mL of Simulated Intestinal Fluid (SIF) solution at pH 7 (containing 6.8 mmol/L KCl, 0.8 mmol/L KH_2_PO_4_, 85 mmol/L NaHCO_3_, 38.4 mmol/L NaCl, and 0.33 mmol/L MgCl_2_(H_2_O)_6_), 2 mg/mL of pancreatic enzymes, 2 mg/mL of porcine bile salts, and 25 μL of CaCl_2_ were added to the samples that had completed gastric digestion. The samples were oscillated at a constant temperature of 37 °C for 4 h. After oscillation, the enzymes were inactivated through a boiling water bath, and the samples were freeze-dried and stored at −80 °C for future use.

### 2.6. Circular Dichroism Analysis

Based on the results of previous experiments on antioxidant activity, GCP-II was initially selected for secondary structure determination. A certain amount of purified GCP-II freeze-dried powder and its digestive products were weighed and prepared as 0.5 mg/mL solutions for circular dichroism analysis. The scanning wavelength range was set from 190 nm to 250 nm, with a frequency of 50 nm/min and a response time of 1 s.

### 2.7. Amino Acid Composition Analysis

Amino acid analysis was performed on the GCP-II digestion products. The method for amino acid composition analysis was the same as in Section 2.3.

### 2.8. Determination of Antioxidant Stability of GCPs

To investigate the influence of processing environments and food ingredients on the antioxidant activity stability of GCPs, GCP-II was used as the main sample to determine the stability of its antioxidant activity in different temperatures, concentrations, and food component environments. Based on the experimental results in Section 2.4, selected antioxidant activity indicators were used as criteria for assessing antioxidant stability, providing a comprehensive evaluation of the antioxidant stability of GCPs.

#### 2.8.1. Effect of Temperature on the Antioxidant Stability of GCPs

GCP freeze-dried powder was dissolved in ultrapure water to prepare a 1 mg/mL sample solution as control group *A*_0_. The same concentration of sample solution was placed in water baths at 4 °C, 50 °C, and 100 °C for 1 h, respectively. Their antioxidant activities were measured and compared with the control group. The calculation formula is as follows:

(1) $$Antioxidant\;Stability\left(\%\right)=\frac{A}{A_{0}}\times 100$$

where *A* represents the antioxidant activity of the GCPs after different treatments, and *A*_0_ represents the antioxidant activity of the untreated GCPs.

#### 2.8.2. Effect of NaCl, Glucose, and Citric Acid on Antioxidant Stability of GCPs

GCP freeze-dried powder was dissolved in ultrapure water to prepare a 1 mg/mL sample solution as control group *A*_0_.

NaCl was added to the same concentration of sample solution to adjust the NaCl percentage concentration to 1%, 2%, and 4%, respectively. Glucose was added to the same concentration of sample solution to adjust the glucose percentage concentration to 1%, 4%, and 7%, respectively. Citric acid was added to the same concentration of sample solution to adjust the citric acid percentage concentration to 0.04%, 0.12%, and 0.20%, respectively.

The mixtures were homogenized and allowed to stand at room temperature for 1 h. Their antioxidant activities were measured and compared with the control group.

#### 2.8.3. Study of Antioxidant Stability of Digestive Products of GCPs

The freeze-dried powder of GCP-II digestive products at the same concentration was used to investigate the antioxidant stability after digestion, and the antioxidant activities were measured according to the method in Section 2.4 of this study.

### 2.9. Structural Identification of GCPs

Based on previous experiments, the sample with the highest comprehensive antioxidant activity was selected for sequence identification and subsequent experiments. Mass spectrometry identification of the sample was conducted by Scientific Compass. The experimental procedure was as follows: the freeze-dried sample was redissolved, and 10 kDa ultrafiltration was performed to remove impurities and desalt the sample before mass spectrometry detection.

After the experiment, the raw mass spectrometry files were searched using MaxQuant 1.5.5.1 for peptide sequence identification and quantitative analysis.

### 2.10. Solid-Phase Synthesis of Peptides

To further validate the purity of the synthesized peptides for subsequent experiments, mass spectrometry analysis was conducted by Jiangsu GenScript Biotechnology Corporation. The parameters were set as follows: nebulizer gas flow rate of 1.5 L/min; CDL temperature of 250 °C; drying gas flow rate of 5 L/min; blocking temperature of 200 °C; T-flow rate of 0.2 mL/min; and solvent B consisting of 50% H_2_O + 50% MeOH.

### 2.11. Prediction of Physicochemical Properties of Peptides

The methods for predicting the physicochemical properties of the synthesized peptides were shown in Table 2.

### 2.12. Verification of Antioxidant Activity of Peptides

The antioxidant activities of synthetic peptides (Section 2.10) prepared via solid-phase synthesis (Section 2.10) were evaluated using ABTS radical scavenging assay, DPPH radical scavenging assay, hydroxyl radical scavenging assay, superoxide anion radical scavenging assay, and ferrous ion chelating assay as described in Section 2.4.

### 2.13. Determination of Synergistic Effects of Peptides

Based on the results of the antioxidant activity verification experiments, two or more synthetic peptides were mixed in equal amounts to prepare samples of a certain concentration, and antioxidant activity experiments were performed. The results obtained from the synergistic experiments were considered as measured values, while the weighted average of the antioxidant capacities of each individual peptide served as the corresponding actual value. By comparing the theoretical values with the measured values, the synergistic or antagonistic relationships among the selected peptides in terms of antioxidant and whitening activities were determined.

## 3. Results and Discussion

### 3.1. Amino Acid Composition Analysis of GCPs

The amino acid composition analysis was conducted to identify the types and quantities of amino acids present in the peptide mixture, which is critical for understanding their antioxidant properties. In total, 17 amino acids were detected, including 6 essential amino acids and 7 hydrophobic amino acids. As shown in Table 3, the total amino acid content in GCPs was 69.092 g/100 g, with hydrophobic amino acids accounting for 20.97 g/100 g and essential amino acids for humans accounting for 24.084 g/100 g. Phenylalanine, the most abundant amino acid in GCPs, serves as a precursor for synthesizing other compounds with antioxidant activity, such as phenylacetic acid and phenyllactic acid [24], thus having an impact on the antioxidant activity of substances [25]. Other hydrophobic amino acids, such as Ala, Leu, and Ile, have also been found to play a role in antioxidant activity [26]. Additionally, studies have shown that arginine possesses antioxidant properties, and its content in GCPs was relatively high [27] at 5.270 g/100 g. Therefore, it was speculated that GCPs have potential antioxidant functions.

### 3.2. In Vitro Assessment of Antioxidant Activity of GCPs

**Figure 1:**
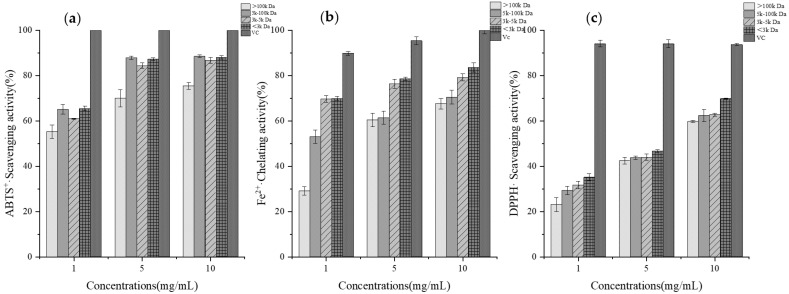

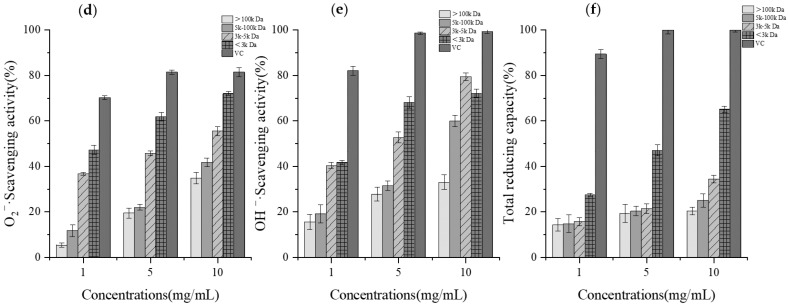
ABTS^+^· scavenging activity (**a**), DPPH· scavenging activity (**b**), OH^−^·scavenging activity (**c**) of GCPs, O_2_^−^· scavenging activity (**d**), total reducing capacity (**e**), Fe^2+^· chelating activity (**f**) with different molecular weight and concentration of GCPs.

The antioxidant activity of GCPs with different molecular weights and concentrations was determined experimentally, as shown in Figure 1. “VC” refers to Vitamin C, used as a positive control in the antioxidant assays.

The analysis revealed that the purified GCPs exhibited considerable antioxidant activity in vitro. The antioxidant activity of GCPs increased with concentration. The effect of molecular weight on its antioxidant activity was unstable, but in most cases, GCPs with lower molecular weight had stronger antioxidant capacity.

**Figure 2:**
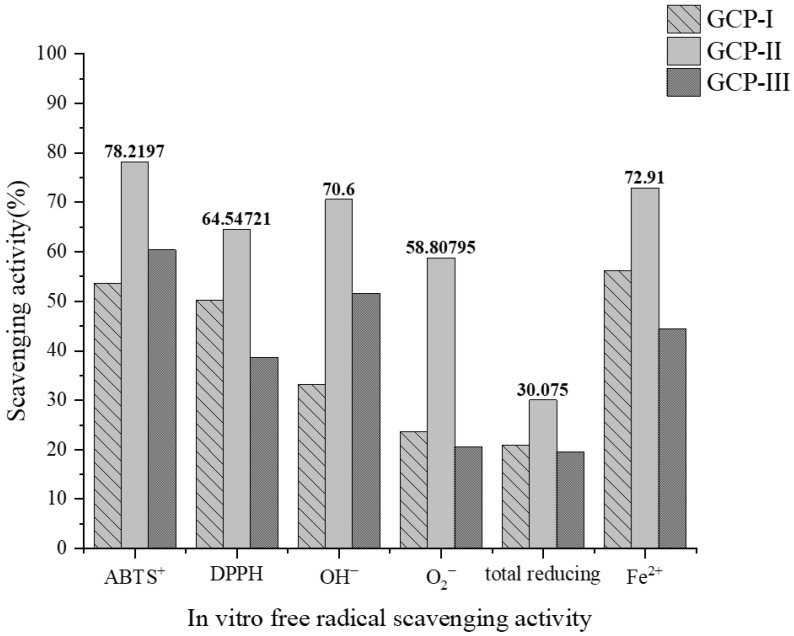
In vitro antioxidant activity of GCP-I, GCP-II, and GCP-III at same concentration.

GCPs with molecular weight <3 kDa were further purified by G-15 dextran gel chromatography to obtain GCP-I, GCP-II, and GCP-III. As shown in Figure 2, after in vitro antioxidant activity assays of these three fractions, it was found that GCP-II exhibited the highest antioxidant activity in vitro. Its ABTS radical scavenging activity and ferrous ion chelating ability were relatively good, with maximum values reaching 78.22% and 72.91%, respectively. In comparison, its DPPH radical scavenging activity was weaker, and its total reducing power was only 30.08%.

### 3.3. Secondary Structure Analysis of GCPs and Digestive Products

**Figure 3:**
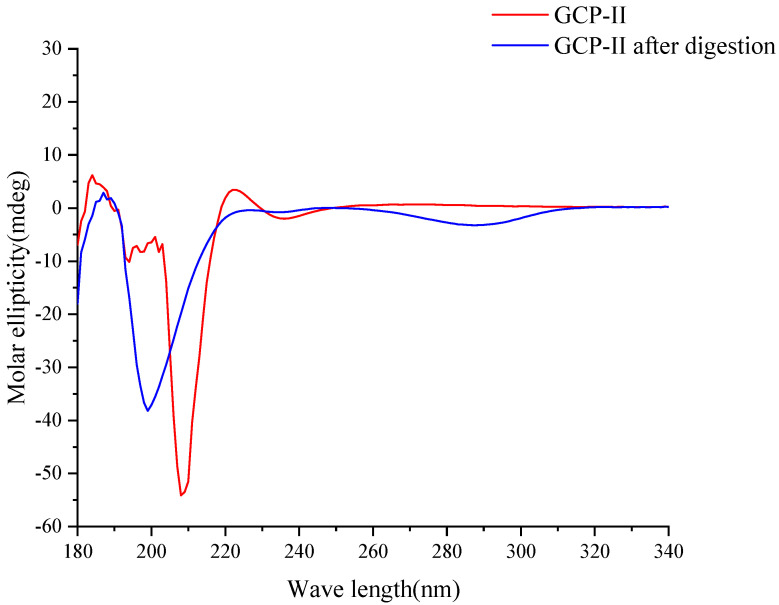
The effect of in vitro simulated digestion on the secondary structure of GCP-II under the circular dichroism.

The secondary structures of collagen primarily include α-helix, β-sheet, β-turn, and random coil [28]. During digestion, these structures were broken down into smaller peptide fragments and amino acids due to the action of enzymes, resulting in changes to the secondary structures. As shown in Figure 3, GCP-II exhibited a distinct positive absorption peak near a wavelength of 190 nm and notable negative absorption peaks at 208 nm and near 230 nm, indicative of a typical α-helix structure [29]. Its β-sheet structure was present near 208 nm, manifesting as a negative absorption peak, while the positive absorption peak at 222 nm suggests that GCP-II also possesses a β-sheet structure [30,31]. In contrast, the digestive products of GCP-II exhibited a significant negative absorption peak near 200 nm, indicating that the original secondary structures have been disrupted by digestion, leading to the formation of a disordered structure of random coils. Additionally, the negative absorption peaks of GCP-II digestive products in the range of 260–310 nm further confirm the generation of random coil structures.

### 3.4. Analysis of Amino Acid Composition of Digestive Products of GCPs

To investigate the impact of simulated in vitro digestion experiments on the amino acid composition of GCPs, GCP-II—which exhibited the highest overall antioxidant activity in previous experiments—and its digestive products were selected as samples for further research and discussion. As shown in Table 4, glycine and arginine were abundant in GCP-II, with concentrations reaching 7.032 g/100 g and 2.819 g/100 g, respectively. These two amino acids were often considered to have a strong correlation with the antioxidant activity of substances, indicating that the notable antioxidant capacity of GCP-II. During the simulated in vitro digestion experiments, the amino acid composition of the peptides may undergo a series of changes, primarily influenced by the enzymatic action during the simulated digestion process, the digestion conditions, and the intrinsic properties of the peptides themselves. Following a series of digestive reactions, the total amino acid content in GCP-II decreased to 1.448 g/100 g, with nearly all detected amino acids showing substantial reductions in their concentrations. The contents of glycine, arginine, and hydrophobic amino acids such as phenylalanine and valine, which were known to influence antioxidant activity, also decreased significantly. Therefore, it was speculated that the in vitro antioxidant capacity of the digestive products of GCP-II will be markedly reduced after digestion treatment.

### 3.5. Impact of Simulated In Vitro Digestion on Antioxidant Activity of GCPs

**Figure 4:**
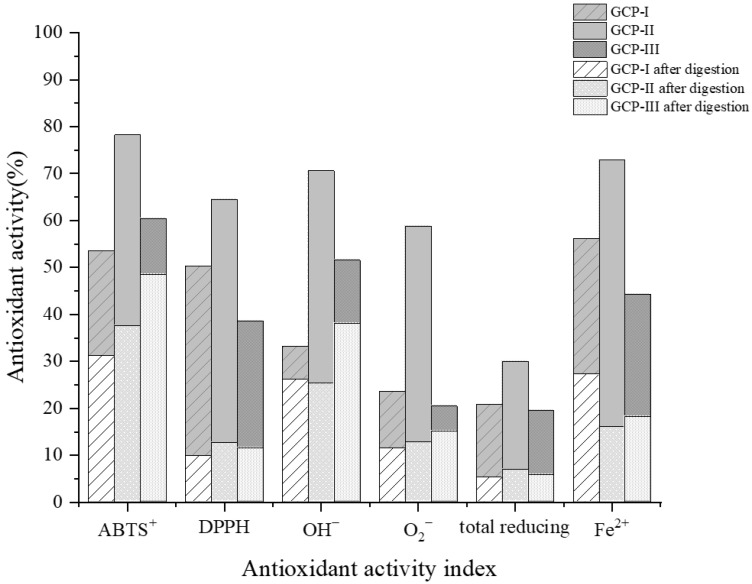
In vitro free antioxidant activity of GCP-I, GCP-II, and GCP-III before and after digestion.

Studies have indicated that during simulated gastrointestinal digestion, the action of digestive enzymes may lead to the cleavage or hydrolysis of peptides, altering their structure and potentially affecting their antioxidant activity [32]. As shown in Figure 4, after undergoing the complete simulated in vitro digestion process, all active components isolated through chromatography exhibited varying degrees of decline in their antioxidant activity. Specifically, the DPPH radical scavenging rates dropped from their original values of 50.29%, 64.55%, and 38.63% to 9.68%, 12.43%, and 11.33%, respectively, representing the largest decrease among the tested indices. It was speculated that during gastrointestinal digestion, GCPs undergo further enzymatic hydrolysis into smaller peptide fragments and amino acids, and the antioxidant activity of these newly generated small molecules was significantly lower than that of the original active fragments of GCPs. Observations from gastrointestinal digestion experiments revealed that GCPs exhibited relatively stable ABTS radical scavenging activity and hydroxyl radical scavenging activity during the digestion process. Based on these findings, it was hypothesized that certain antioxidant functions of GCPs possess good digestive stability.

### 3.6. Antioxidant Stability Assessment Results of GCP-II

The determination of the antioxidant stability of peptides was of great significance for evaluating their antioxidant capacity, guiding applications, exploring mechanisms of action, and facilitating research and development, as well as optimization. Based on the results of previous experiments, GCP-II was selected as the main object of the antioxidant stability experiment for its great antioxidant activity.

#### 3.6.1. Impact of Temperature on Antioxidant Stability of GCP-II

**Figure 5:**
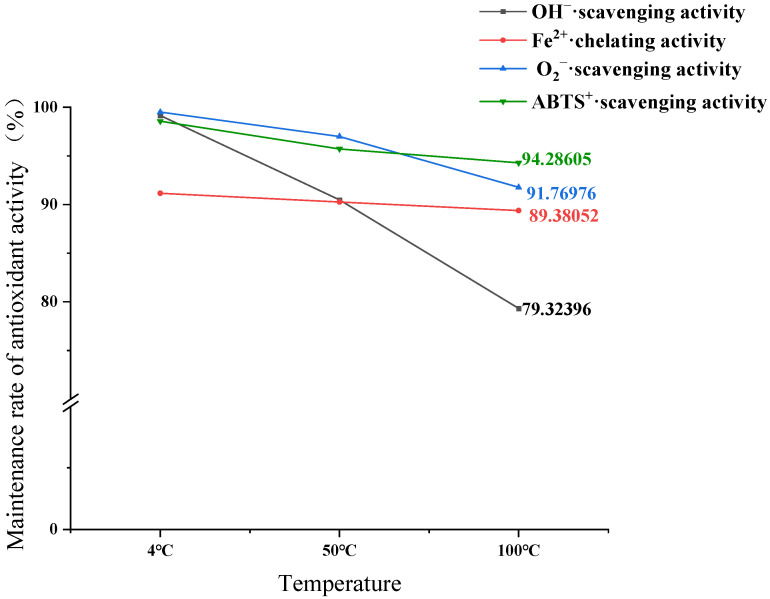
The maintenance rate of the antioxidant activity of GCP-II in the range of 4 °C to 100 °C.

As shown in Figure 5, GCP-II generally exhibits good antioxidant stability at 4 °C, maintaining around 90% activity. The antioxidant property most affected by temperature was its hydroxyl radical scavenging activity, which decreases significantly as the temperature rises. When heated to 100 °C, the retention rate of hydroxyl radical scavenging activity was 79.32%. The observed decline in hydroxyl radical scavenging activity at elevated temperatures was attributed to the denaturation of heat-sensitive residues, such as histidine and tyrosine, which are critical for radical neutralization via hydrogen donation or electron transfer [33].

Both the Fe^2+^ chelating ability and superoxide anion scavenging capacity of GCP-II display considerable thermal stability, with antioxidant retention rates still above 90% at 100 °C. Overall, the antioxidant activity of GCPs demonstrates good thermal stability in the range of 4 °C to 100 °C.

#### 3.6.2. Impact of Food Ingredient Components on Antioxidant Stability of GCP-II

**Figure 6:**
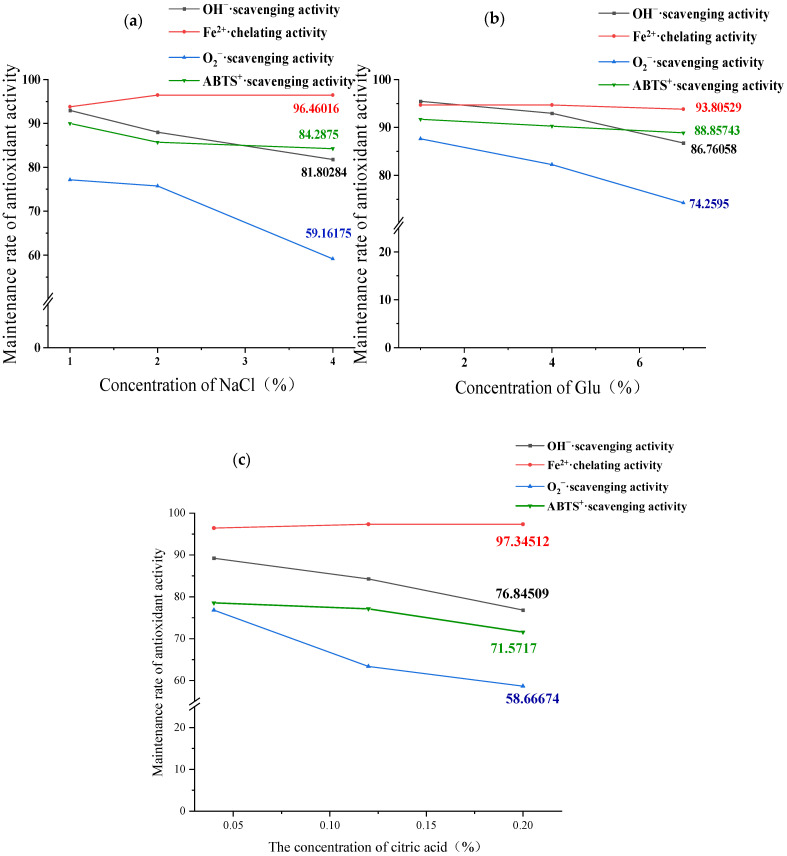
Effects of different concentrations of NaCl (**a**), glucose (**b**), and citric acid (**c**) on the antioxidant activity of GCP-II.

The effects of common food ingredient components on the antioxidant stability of GCP-II are illustrated in Figure 6. Among them, the ferrous ion chelating ability of GCP-II was largely unaffected by various concentrations and types of food ingredients, remaining stable at over 95%. In contrast to its thermal stability, the superoxide anion scavenging capacity of GCP-II was more significantly influenced by food components. Both NaCl and citric acid can significantly reduce its stability when their concentrations reach 4% and 0.2%, respectively. Among them, the superoxide anion scavenging ability of GCP-II was most affected by the composition of food ingredients. When the concentration of NaCl reached 1%, the superoxide anion scavenging retention rate decreased to 77.15%, and when the concentration of NaCl reached 4%, the superoxide anion scavenging retention rate decreased to 59.16%. In addition, in the experiment on the effect of different concentrations of citric acid on the antioxidant stability of GCP-II, the ABTS^+^ scavenging ability was also greatly affected, and its activity retention rate was 71.57% when the concentration of citric acid was 0.2%. Therefore, during the processing and storage of GCP-II, it was advisable to avoid high-concentration salt environments and acidic conditions to the greatest extent possible.

### 3.7. Structural Identification of GCP-II

**Figure 7:**
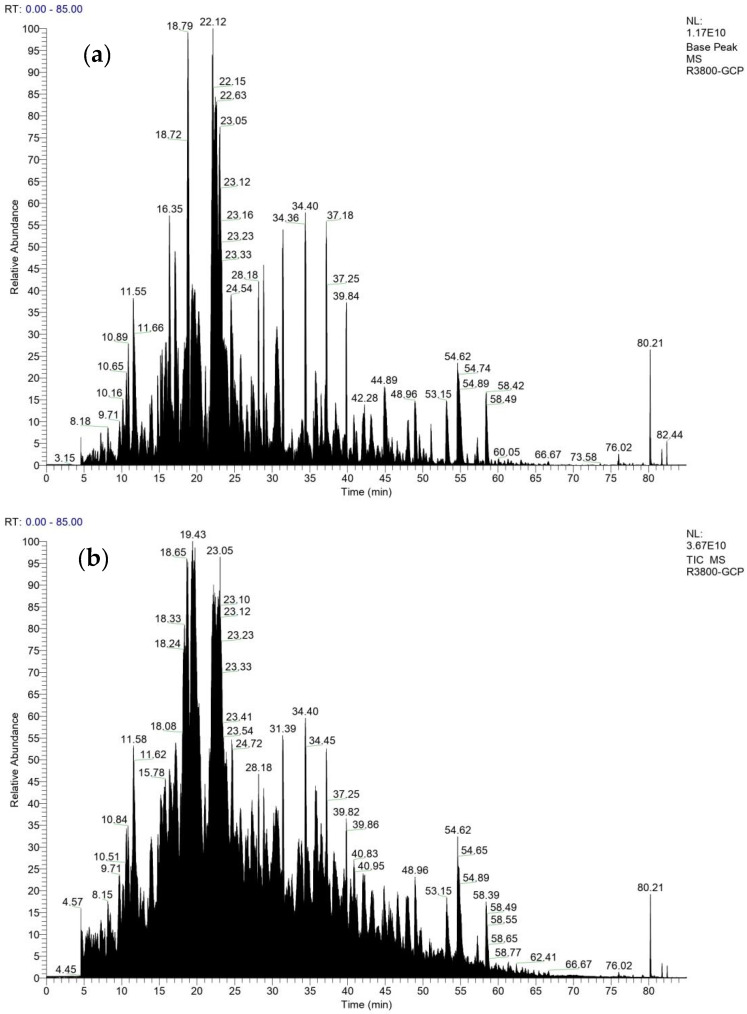
Base Peak Chromatogram (**a**) and Total Ion Chromatogram (**b**) of GCP-II.

Structural identification of GCP-II was conducted using LC-MS/MS, resulting in the detection of 464 sequences. Among these, 137 sequences were composed of 3 to 7 amino acids, and 327 sequences were composed of 8 to 25 amino acids. A total of 295 sequences had a confidence score of 90 or above. The Base Peak Chromatogram (BPC) of GCP-II is shown in Figure 7a below, while its Total Ion Chromatogram (TIC) is presented in Figure 7b. By analyzing the BPC spectrum, researchers can gain insights into the distribution of different components in the sample and their relative abundances [34]. This analysis aids in identifying the peptide components within the sample and provides an initial assessment of their concentrations. In contrast to the BPC, the TIC reflects the chromatogram of all ions in the sample, providing comprehensive information about the overall distribution and abundance of all ions in the sample [35]. It thus offers a more holistic view of the chromatographic distribution of all ions within the sample.

### 3.8. Peptide Selection and Prediction of Physicochemical Properties

Seven active fragments with potential antioxidant activity from GCP-II were identified and selected through a comparison with the BIOPEP database. Their specific sequences and the predictions of physicochemical properties are as follows (Table 5).

**Figure 8:**
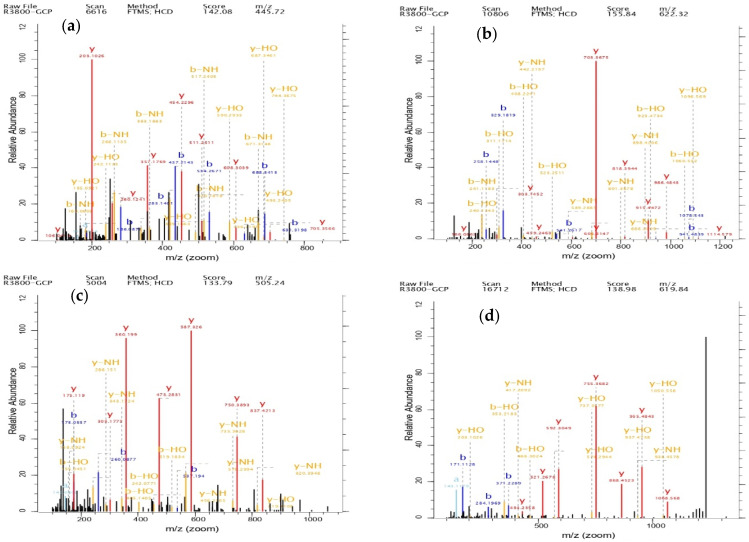

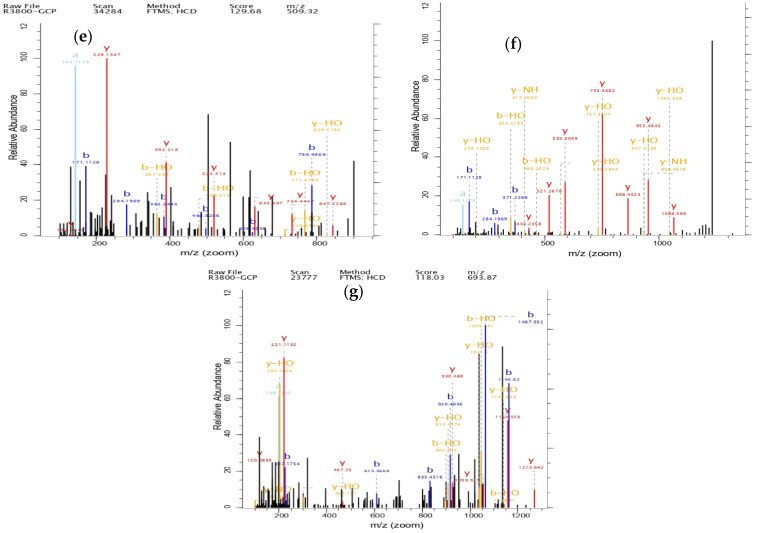
The mass spectrum of EKAPDPFRHF (**a**), QGPPGPPGPS (**b**), GERGPPGPM (**c**), DGSYNIGQR (**d**), GILTLKYPI (**e**), VLSLYASGRTT (**f**), and ILTERGYSFVTT (**g**).

After conducting mass spectrometry analysis on these seven peptides using LC-MS/MS, the mass spectra are presented as shown in Figure 8 below:

After screening, seven peptide sequences, namely EKAPDPFRHF, GILTLKYPI, GERGPPGPM, ILTERGYSFVTT, QGPPGPPGPS, VLSLYASGRTT, and DGSYNIGQR, were selected for polypeptide synthesis. The screening process primarily considered the antioxidant activities of the sequences recorded in BIOPEP-UWM, along with the comprehensive scores from mass spectrometry analysis, the number of times they were tested, and their peak intensities. The comprehensive score of a peptide sequence indicates its reliability; the seven selected peptide sequences all had scores above 100, suggesting high reliability. Peak intensity represents the signal strength of the peptide sequence, which can be used for quantitative analysis and as a criterion for peptide selection. Among the seven selected peptide sequences, four peptide sequences were predicted to have good water solubility, at least six were non-toxic, and all seven contained amino acid sequences that were documented to have high antioxidant activity, such as LY, RHF, LK, TERGY, GPP, LKYPI, YNI, and YA. Therefore, it was speculated that these seven peptide sequences possessed high antioxidant activity.

### 3.9. Verification of Physicochemical Properties of Synthesized Peptides

The seven peptide sequences from Section 3.8 were synthesized and named as peptide 1, peptide 2, peptide 3, peptide 4, peptide 5, peptide 6, and peptide 7, respectively, from top to bottom according to Table 6.

The synthesis of specific peptides derived from GCP-II was conducted to validate the contribution of individual sequences to the observed antioxidant activity and eliminate potential confounding effects from the crude hydrolysate. By isolating and characterizing these peptides, we aimed to confirm that the bioactivity identified in GCP-II originated from discrete peptide sequences rather than synergistic interactions within the mixture, and to identify key structural motifs (e.g., aromatic residues, acidic amino acids) responsible for antioxidant efficacy. This approach aligns with established methodologies for bioactive peptide research, ensuring reproducibility and mechanistic clarity while advancing the development of targeted antioxidant agents from underutilized biomass.

#### 3.9.1. Purity Information of Synthesized Peptides

**Figure 9:**
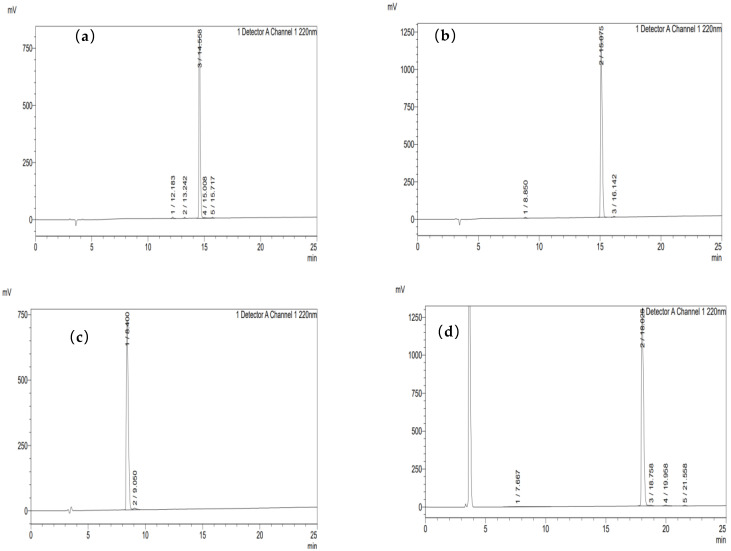

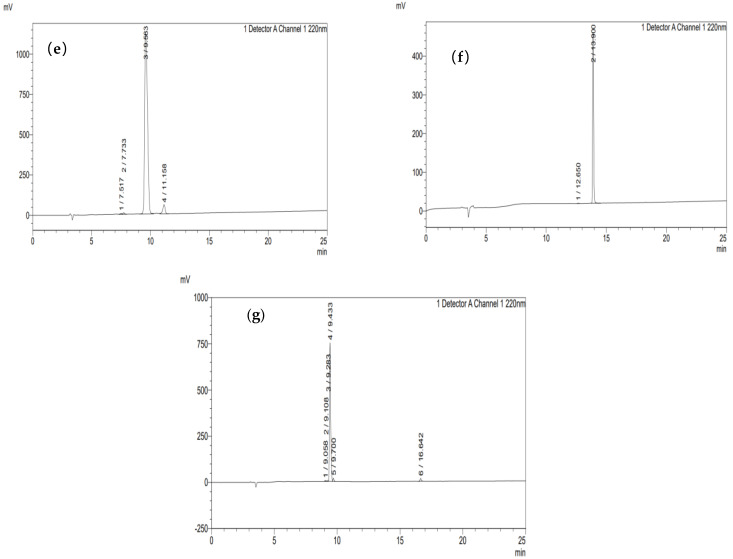
HPLC plots of peptide 1 (**a**), peptide 2 (**b**), peptide 3 (**c**), peptide 4 (**d**), peptide 5 (**e**), peptide 6 (**f**), and peptide 7 (**g**).

As observed from the HPLC chromatograms (Figure 9), the purity of all seven synthesized peptides reached above 95%, making them suitable for subsequent research on antioxidant activity and tyrosinase inhibition.

#### 3.9.2. Verification of Mass Spectrometry Information for Synthesized Peptides

**Figure 10:**
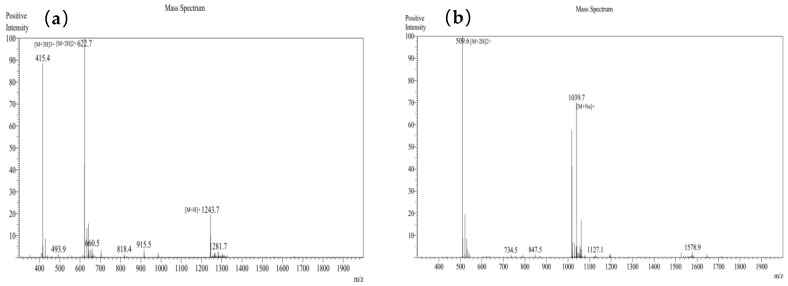

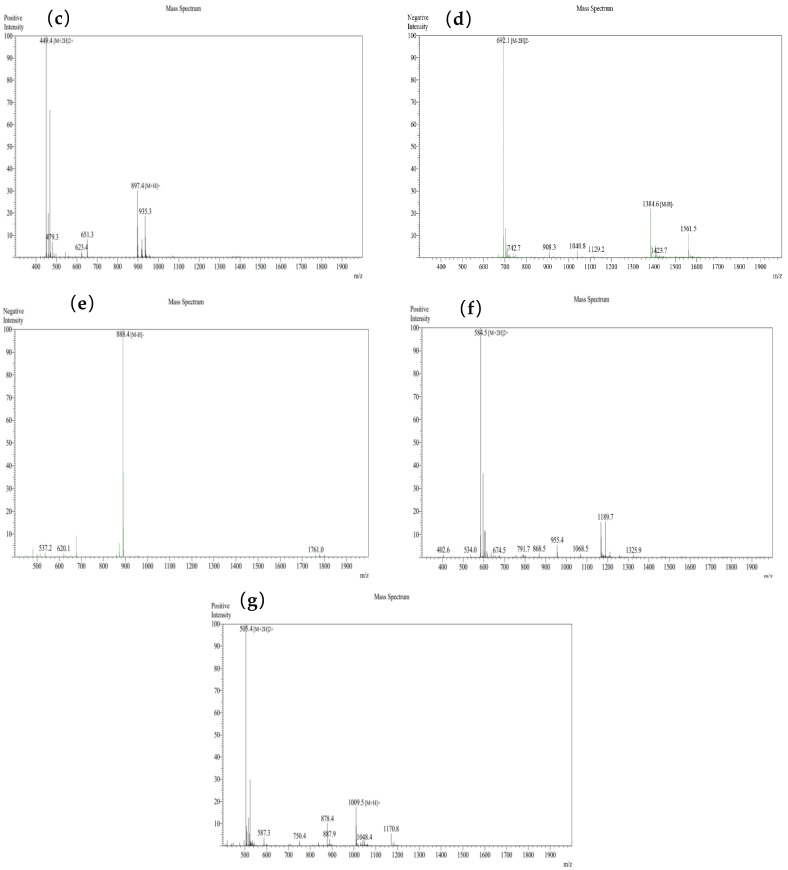
Mass spectrum of peptide 1 (**a**), peptide 2 (**b**), peptide 3 (**c**), peptide 4 (**d**), peptide 5 (**e**), peptide 6 (**f**), and peptide 7 (**g**).

Based on the mass spectrometry analysis from Jiangsu GenScript Biotechnology Corporation, the mass spectrometry results for the seven peptides are presented in Figure 10.

#### 3.9.3. Verification of Antioxidant Activity of Synthetic Peptides

##### Determination of ABTS Radical Scavenging Activity

**Figure 11:**
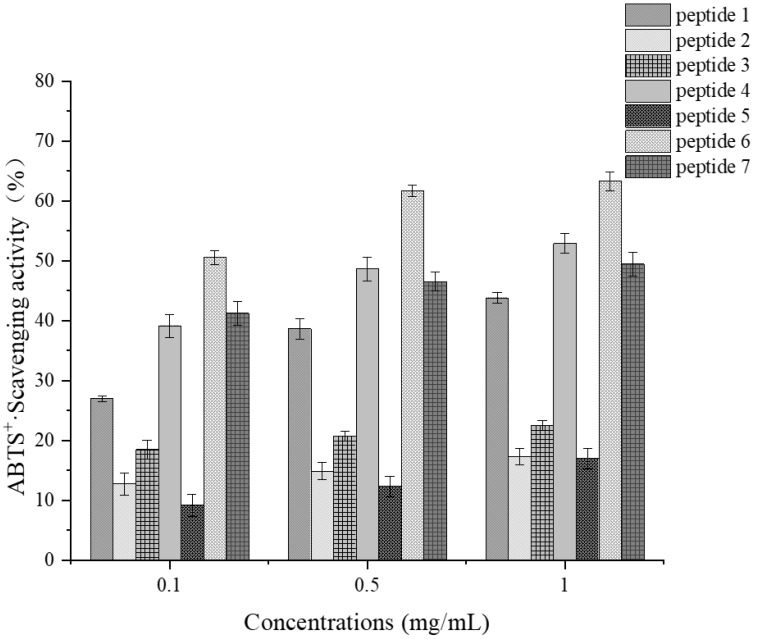
ABTS^+^· scavenging activity at various concentrations for each synthetic peptide.

As shown in Figure 11, the overall ABTS radical scavenging activity of the seven synthetic peptide segments is relatively good. Among them, peptide 6 exhibits the highest activity, with a scavenging rate of 63.31% at a concentration of 1 mg/mL. In addition to peptide 6, peptides 1, 4, and 7 also demonstrate considerable ABTS radical scavenging abilities, all exceeding 40%. Notably, these three peptides contain the aromatic amino acid Y (Tyr). Studies have indicated that all phenolic hydroxyl groups on tyrosine significantly impact the antioxidant activity of active substances [36]. These hydroxyl groups can act as hydrogen donors to capture radicals, and the phenoxy radicals released after a series of reactions exhibit strong stability, thereby enhancing the ABTS radical scavenging capacity of these active substances [37]. Furthermore, research has found that H (His) also exerts a certain influence on the antioxidant activity of active fragments [38,39].

However, in comparison, the antioxidant activity of the peptide GQCHV was substantially improved. For the peptide GQCH, both activities were substantially enhanced. When C was replaced with A, both activities were significantly reduced, and the removal of H resulted in the loss of antioxidant activity [40]. This indicates that the aromatic amino acids Tyr (Y) and His (H) have a beneficial effect on the ABTS radical scavenging activity of substances [41].

##### Determination of DPPH Radical Scavenging Activity

**Figure 12:**
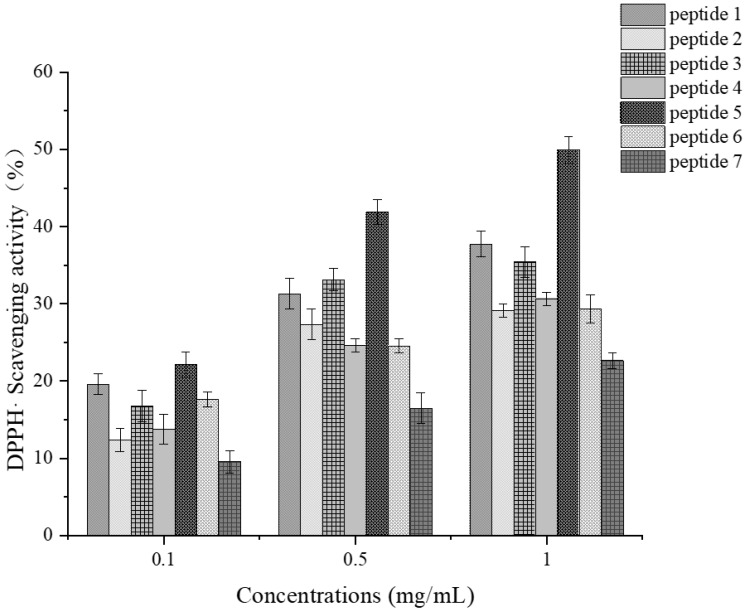
DPPH· scavenging activity at various concentrations for each synthetic peptide.

The DPPH radical scavenging activity of each active fragment increased steadily with increasing concentration, but the overall scavenging activity was lower than that observed for ABTS radicals, which was similar to the trend observed for GCPs. As shown in Figure 12, peptide 5 exhibited the relatively strongest DPPH radical scavenging activity, reaching 49.98%, followed by peptides 1 and 3 with 37.73% and 35.41%, respectively. The higher scavenging activity of peptide 5 may be attributed to the significant impact of proline at the third position from its N-terminus on its antioxidant properties, which was similar to the results obtained by Rodríguez, M. [42] for the DPPH radical scavenging activity of short peptides synthesized from oligopeptide sequences derived from the enzymatic hydrolysis products of grass carp fish meat.

Furthermore, researchers studied and analyzed the DPPH radical scavenging activity of GL-9 and found that it exhibited high scavenging activity with a trend similar to that of glutathione [43]. Another study indicated that the sequence obtained from the digestive hydrolysates of halfbeak anchovy possesses relatively high DPPH radical scavenging ability [44]. Researchers extracted a peptide with a sequence from cheddar cheese, which exhibited DPPH radical scavenging activity comparable to that of commercial antioxidants such as BHA, t-BHQ, and ferulic acid [45].

Taken together, the content of proline, histidine, glycine, and tryptophan has a significant impact on the DPPH radical scavenging capacity of active fragments.

##### Determination of Hydroxyl Radical Scavenging Activity

**Figure 13:**
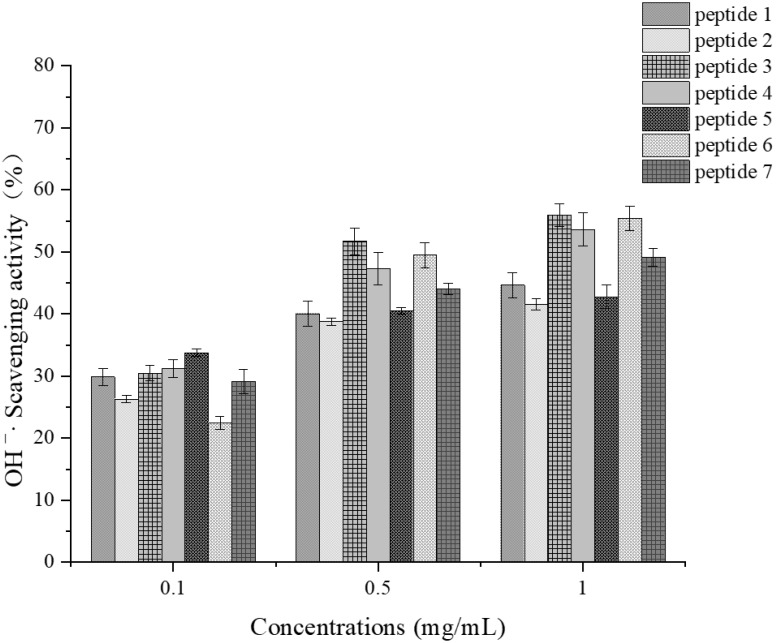
OH^−^· scavenging activity at various concentrations for each synthetic peptide.

As shown in Figure 13, in the experiment assessing hydroxyl radical scavenging activity, peptides 3 and 6 demonstrated strong scavenging capabilities, with scavenging rates of 55.98% and 55.42%, respectively, at a concentration of 1 mg/mL. Overall, the hydroxyl radical scavenging activity of all synthetic peptides was considerable, with scavenging rates almost exceeding 30% at the highest concentration and maintaining above 10% even at the lowest concentration of 0.1 mg/mL. Notably, all seven peptides synthesized in our experiment contained hydrophobic amino acids to a high degree overall, which may account for their high hydroxyl radical scavenging activity [46].

Additionally, studies have found that glycine (Gly) has a significant influence on the antioxidant activity of substances. Peptide 3, which has a high glycine content [47], also exhibited high hydroxyl radical scavenging activity, aligning with these findings.

##### Determination of Superoxide Anion Scavenging Activity

**Figure 14:**
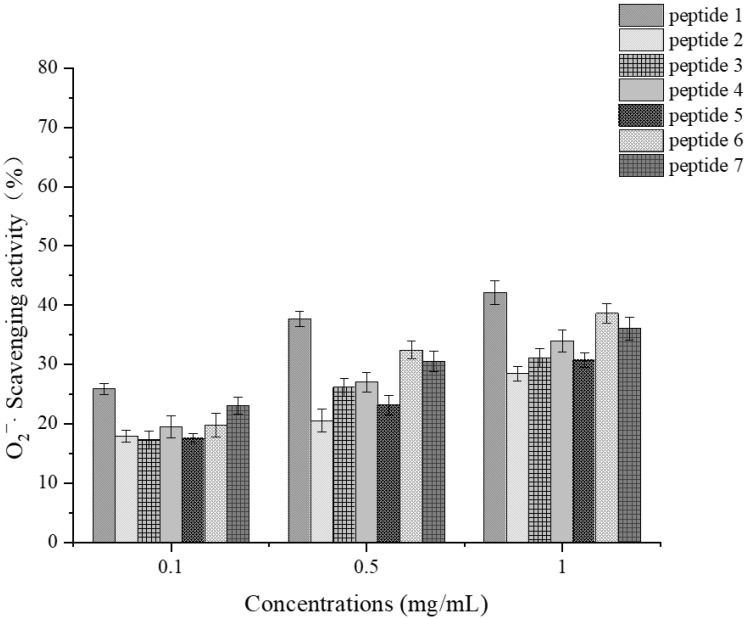
O_2_^−^· scavenging activity at various concentrations for each synthetic peptide.

In the superoxide anion scavenging experiments, the synthetic peptides generally exhibited strong scavenging capabilities. As shown in Figure 14, among them, peptide 1 demonstrated the highest scavenging activity, reaching 42.14%, followed by peptides 6 and 7 with scavenging rates of 38.61% and 36.09%, respectively. Studies have speculated that aspartic acid present in peptide sequences may significantly contribute to their superoxide anion scavenging ability. Compared to other synthetic fragments, peptides 1 and 7 contain aspartic acid, aligning with the research direction inferred from these studies [48]. Additionally, the sequence which contains aspartic acid (D) exhibited the highest overall antioxidant activity and demonstrated strong superoxide anion scavenging ability in mouse oxidative stress response experiments [49].

Furthermore, studies have indicated that valine (Val) or leucine (Leu) at the N-terminus can positively affect the antioxidant activity of active fragments. Peptide 6, which has valine at its *N*-terminus and is rich in hydrophobic amino acids, was speculated to possess high superoxide anion scavenging ability due to these characteristics [50].

##### Determination of Ferrous Ion Chelating Ability

**Figure 15:**
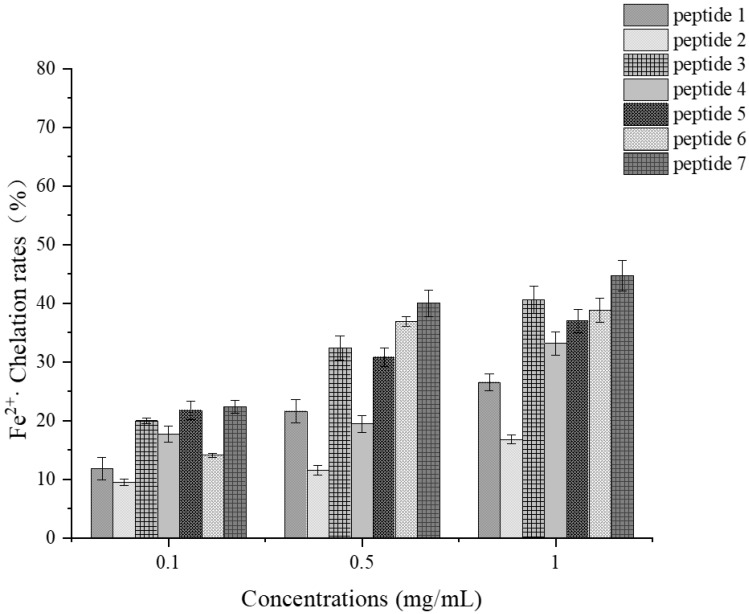
Fe^2+^· chelation rates at various concentrations for each synthetic peptide.

As illustrated in Figure 15, peptides 7 and 3 exhibit strong ferrous ion chelating abilities, while peptide 2 demonstrates the weakest metal chelating ability, with a chelating rate of only 16.81% at a concentration of 1 mg/mL. At the same concentration, peptide 7 achieves a chelating rate of 44.72%, and peptide 3 reaches 40.59%. Both of these peptides contain two acidic amino acids, glutamic acid (E) and aspartic acid (D). Studies have indicated that acidic amino acid residues can chelate transition metal ions, thereby achieving antioxidant effects [51]. It was speculated that the presence of these two acidic amino acids may contribute to the strong metal chelating abilities of these two peptides [52,53]. Similarly, peptide 7 not only contains abundant hydrophobic amino acids but also is enriched with acidic amino acids such as glutamine (Q), tyrosine (Y), tryptophan (W), and asparagine (N). This might be one of the reasons for the good antioxidant activity of peptide 7.

### 3.10. Analysis of Synergistic Interactions in Antioxidant Activity

Based on the experimental results from Section 3.9.3, peptides 3 and 6 were selected for evaluation of their synergistic effects. Considering the antioxidant activities of these two peptides, DPPH radical scavenging ability, hydroxyl radical scavenging ability, and superoxide anion scavenging ability were chosen as the indicators for assessing their synergistic effects.

**Figure 16:**
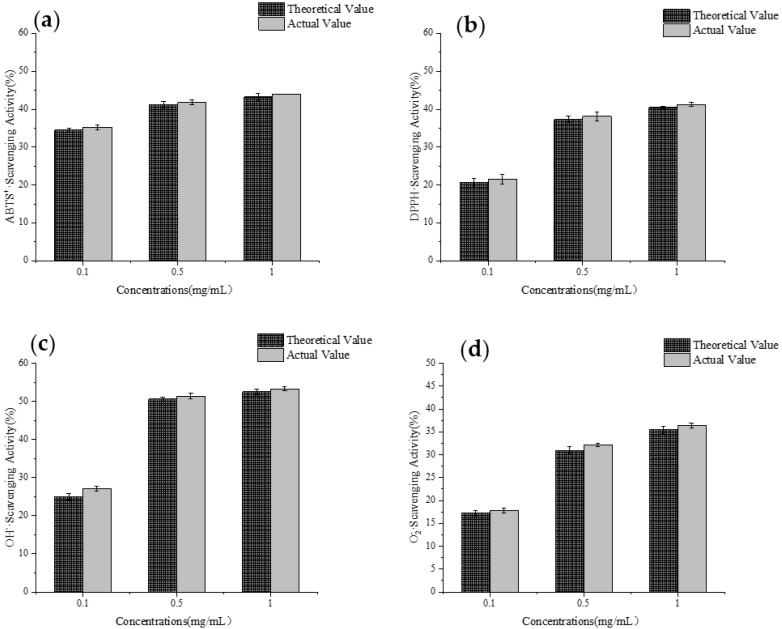
Analysis of synergistic effect of synthetic peptides. ABTS^+^· scavenging activity (**a**), DPPH· scavenging activity (**b**), OH^−^·scavenging activity (**c**) of GCPs, O_2_^−^· scavenging activity (**d**) with different concentration of theoretical value and actual value.

As shown in Figure 16, with increasing concentrations, these two peptides demonstrated a certain degree of synergistic effect overall. In the ABTS radical scavenging experiment and superoxide anion scavenging experiment, the actual values for peptides 3 and 6 at concentration gradients of 0.1 to 1 mg/mL were higher than the theoretical values. Similarly, in the DPPH radical scavenging experiment and hydroxyl radical scavenging experiment, peptides 3 and 6 also exhibited notable synergistic effects, with the most pronounced effect observed at a concentration of 0.1 mg/mL. At this concentration, the DPPH radical scavenging ability of peptides 3 and 6 increased from a theoretical value of 20.65% to an actual value of 21.53%, surpassing theoretical values by 0.88% [54]. Under the same concentration, their hydroxyl radical scavenging ability also increased from 24.96% to 27.12%, surpassing theoretical values by 2.16% [55].

In a word, it was concluded that peptides 3 and 6 exhibited a certain degree of synergism in antioxidant activity experiments at different concentrations.

## 4. Conclusions

Through systematic experimental design and data analysis, this study conducted an in-depth exploration of the preparation, purification, structural characterization, antioxidant activity, and stability of collagen peptides derived from grass carp swim bladders (GCPs). Then, ultrafiltration and chromatography were performed on the preliminarily prepared GCPs, and the samples obtained by chromatography were named GCP-I, GCP-II, and GCP-III, respectively. After an analysis of the results of previous antioxidant experiments, GCP-II was selected as the main sample for the synthetic peptide experiments. The sequence of GCP-II was analyzed, from which seven bioactive fragments with high antioxidant potential were selected and synthesized. Their sequences were EKAPDPFRHF, GILTLKYPI, GERGPPGPM, ILTERGYSFVTT, QGPPGPPGPS, VLSLYASGRTT, and DGSYNIGQR and were named as peptide 1, peptide 2, peptide 3, peptide 4, peptide 5, peptide 6, and peptide 7, respectively. Among them, the two sequences with the highest comprehensive antioxidant activity were peptide 3 and peptide 6, and these two peptides showed synergistic effects in many antioxidant experiments. The research results indicated that GCPs, as a polypeptide with considerable antioxidant activity, maintains high antioxidant activity under the influence of different environments and concentrations of food ingredients, thus demonstrating good overall stability.

The findings demonstrate that grass carp swim bladder-derived peptides (GCP-II) and their synthetic counterparts exhibit robust antioxidant properties with significant practical potential. The thermal stability of GCP-II (>90% retention at 100 °C) and its resistance to NaCl/citric acid position it as a viable natural preservative for thermally processed foods (e.g., baked goods) and acidic beverages, addressing clean-label demands in the food industry. Additionally, the synergistic effects of peptides 3 and 6 highlight their promise in nutraceuticals targeting oxidative stress-related pathologies. These results advocate for the sustainable valorization of underutilized fish processing by-products in functional ingredient development.

However, there were still some limitations in this study. For instance, although peptide segments with high antioxidant activity have been screened out, further in-depth research is needed to investigate their specific antioxidant mechanisms. Additionally, the safety and effectiveness of GCPs in practical applications need to be verified through more animal experiments and clinical trials.

## Figures and Tables

**Table 1:** Experimental materials, instruments, and equipment.

Items	Manufacturer	Country and City
Fresh grass carp swim bladder	Shanghai Pudong New Area Nanhui new town aquatic products shop	Shanghai, China
Alkaline protease (200 μ/mg)	Shanghai Yuanye Bio-Technology Co., Ltd.	Shanghai, China
Neutral protease (50 μ/mg)	Shanghai Aladdin Biochemical Technology Co., Ltd.	Shanghai, China
NaOH (AR)	Sinopharm Chemical Reagent Co., Ltd.	Shanghai, China
HCl (AR)	Sinopharm Chemical Reagent Co., Ltd.	Shanghai, China
NaCl	Sinopharm Chemical Reagent Co., Ltd.	Shanghai, China
Glucose	Sinopharm Chemical Reagent Co., Ltd.	Shanghai, China
Citric acid	Sinopharm Chemical Reagent Co., Ltd.	Shanghai, China
pH meter	Mettler Toledo International Inc.	Columbus, OH, USA
1,1-Diphenyl-2-picrylhydrazyl	FTY. Phygene Life Sciences Co., Ltd.	Fuzhou, China
ABTS	FTY. Phygene Life Sciences Co., Ltd.	Fuzhou, China
H_2_O_2_	Guangdong Hengjian Pharmaceutical Co., Ltd.	Jiangmen, China
Pyrogallol	Sinopharm Chemical Reagent Co., Ltd.	Shanghai, China
Potassium ferricyanide	Sinopharm Chemical Reagent Co., Ltd.	Shanghai, China
FeCl_3_	Sinopharm Chemical Reagent Co., Ltd.	Shanghai, China
FeCl_2_	Sinopharm Chemical Reagent Co., Ltd.	Shanghai, China
1,10-Phenanthroline	Shanghai Yien Chemical Technology Co., Ltd.	Shanghai, China
Freeze dryer XY-FD-L1	Shanghai XinYU Instrument Co., Ltd.	Shanghai, China
Ultrafiltration membrane	Sartorius AG	Göttingen, Germany
Sephadex G-15	Sigma-Aldrich (Shanghai) Trading Co., Ltd.	Shanghai, China
Total amino acid analyzer LA8080	Hitachi Limited	Hitachi, Japan
Circular dichroism spectrometer	Applied Photophysics Ltd.	Leatherhead, UK
H1750R High-speed refrigerated centrifuge	Xiangyi centrifuge Instrument Co., Ltd	Changsha, China
Visible spectrophotometer	Shanghai Metash Instruments Co., Ltd.	Shanghai, China
LC-MS/MS	Science Compass	Wenzhou, China
Synthetic peptide	Jiangsu Jinsilui Biotechnology Co., Ltd	Yangzhou, China

All other reagents used were analytically pure.

**Table 2:** Methods for predicting the physical and chemical properties of peptides.

Items	Forecasting Methods
Water solubility	Innovagen
Toxicity assessment	ToxinPred
Molecular weight and isoelectric point	Expasy-compute
Net charge and hydrophobicity	Pepdraw

**Table 3:** Amino acid composition of peptides derived from grass carp swim bladder.

Items	Content (g/100 g)
Gly	19.632
Phe *^#^	8.584
Lys *	7.157
Arg	5.370
Leu *^#^	4.454
Glu	4.429
Tyr	4.298
Ala ^#^	2.965
Asp	2.936
Ser	2.084
Ile *^#^	2.012
Thr	1.815
Val *^#^	1.533
Pro ^#^	1.078
His	0.391
Met *^#^	0.344
Cys	0.010

Note: * indicates essential amino acids for humans, and ^#^ indicates hydrophobic amino acids.

**Table 4:** Analysis of amino acid component of GCP-II before and after digestion (g/100 g).

Items	GCP-II	GCP-II After Digestion
Gly	7.032	0.169
Lys *	5.459	0.058
Arg	2.819	0.311
Ser	1.797	0.098
Asp	1.135	0.129
Glu	0.855	0.153
Phe *^#^	0.815	0.104
Thr	0.641	0.071
Val *^#^	0.639	0.075
Leu *^#^	0.487	0.094
Gcu ^#^	0.419	0.068
Ile *^#^	0.304	0.049
Met *^#^	0.207	0.011
His	0.125	0.019
Tyr	0.025	0.038
Pro ^#^	0.009	0.001
Toatal	22.768	1.448

Note: * indicates essential amino acids for humans, and ^#^ indicates hydrophobic amino acids.

**Table 5:** Prediction of physicochemical properties of peptides.

Sequence	Water Solubility	Hydroph-Obicity kcal/mol	Toxicity Assessment	Isoelectric Point	MW (Molecular Weight)	Net Charge
EKAPDPFRHF	High	19.47	Non-toxic	6.85	1243.39	0
QGPPGPPGPS	High	13.28	/	5.52	889.96	0
GERGPPGPM	High	16.54	Non-toxic	6.00	897.02	0
DGSYNIGQR	High	15.90	Non-toxic	6.84	1009.04	0
GILTLKYPI	Lower	6.79	Non-toxic	8.59	1017.28	1
VLSLYASGRTT	Lower	9.11	Non-toxic	8.72	1167.33	1
ILTERGYSFVTT	Lower	10.45	Non-toxic	6.00	1386.57	0

**Table 6:** Information on selected peptides.

Items	Sequence	Length	Score	Frequency of Detection	Peak Intensity
Peptide 1	EKAPDPFRHF	10	155.84	1	3,260,300,000
Peptide 2	GILTLKYPI	9	129.68	2	1,151,900,000
Peptide 3	GERGPPGPM	9	139.14	1	804,920,000
Peptide 4	ILTERGYSFVTT	12	118.03	1	581,870,000
Peptide 5	QGPPGPPGPS	10	142.08	1	359,000,000
Peptide 6	VLSLYASGRTT	11	122.13	3	347,310,000
Peptide 7	DGSYNIGQR	9	133.79	1	313,090,000

## Data Availability

The original contributions presented in the study are included in the article; further inquiries can be directed to the corresponding authors.

## Supplementary Materials

The following supporting information can be downloaded at: https://www.mdpi.com/article/10.3390/foods14071216/s1.
