# Supplementary material for: Physicochemical Properties and Stability of Antioxidant Peptides from Swim Bladder of Grass Carp (Ctenopharyngodon idella)
Source: Foods. 2025 Mar 30;14(7):1216. doi: 10.3390/foods14071216 (PMC12512379; doi:10.3390/foods14071216)
Supplement: Supplementary file 1 [file foods-14-01216-s001.zip › foods-3526957-supplementary.pdf]

foods

*Article*

# Physicochemical Properties and Stability of Antioxidant Peptides from Swim Bladder of Grass Carp (*Ctenopharyngodon idella*)

**Li Suxin [1,†], Gu Jinhui [1,†], Liu Yiyi [1], Qiu Weiqiang [1,*] and Shi Wenzheng [1,2,*]**

[1] College of Food Science and Technology, Shanghai Ocean University, Shanghai 201306, China
[2] Marine Biomedical Science and Technology Innovation Platform of Lin-gang Special Area, Shanghai 201306, China
* The corresponding author of this paper.
† These authors contributed equally to this work.

**Abstract:** Grass carp swim bladder was used as the raw material to prepare grass carp swim bladder collagen peptides (GCPs) through enzymatic hydrolysis. Purification, structural characterization, antioxidant activity assessment, and digestive stability studies were conducted on the GCPs. Initially, GCPs of high purity were obtained by optimizing the enzymatic hydrolysis conditions and purification methods. Subsequently, the obtained GCPs were subjected to six *in vitro* antioxidant activity assessments with different concentrations and molecular weights as gradients. After screening GCP-II with the relatively optimal comprehensive antioxidant activity as the main research object for subsequent studies, it was found that digestion had a significant impact on GCP-II, yet GCP-II exhibited good thermal stability in the range of 4 °C to 100 °C. Additionally, except for its superoxide anion scavenging ability, different concentrations and food ingredients, including NaCl, glucose and citric acid, had little overall impact on its antioxidant activity, suggesting good antioxidant stability. Seven peptide segments were screened from the 464 sequences of GCP-II using LC-MS/MS analysis, based on their antioxidant activity and sequence characteristics, which named peptides 1 to 7, respectively, among which peptides 1, 3, 6, and 7 exhibited higher antioxidant activity. With peptides 3 and 6 as the main research objects, it was found that they generally exhibited the synergism in antioxidant activity experiments.

**Keywords:** peptide; swim bladder; antioxidative activity; stability; peptide synthesis, digestion simulation

## 1. Introduction

Collagen, as one of the most abundant protein in organisms, not only fulfills the vital function of maintaining the morphological and structural integrity of organisms but also demonstrates extensive application prospects in various fields such as medicine, food, and cosmetics due to its biocompatibility, degradability, and unique biological activity. In recent years, with the escalating demand for natural and healthy products, the extraction and development of high-activity, high-value-added collagen products from natural resources have become a market focus. Collagen peptides, which have smaller molecular weights, were more easily absorbed by the human body, and exhibit higher biosafety and utilization rates, have gradually entered the public eye and become one of the research hotspots both domestically and internationally.

Grass carp, as one of the important freshwater fish species in China, is abundant in swim bladder resources, which were previously often discarded or used as low-value feed[1]. However, with the deepening research on collagen peptides from grass carp swim

bladders(GCPs) in recent years, various biological activities such as antioxidant, anti-aging, and wound healing promotion properties have been discovered[2], providing a scientific basis for their development and application in functional foods, cosmetics, and biomedical fields.

Therefore, conducting research on the preparation, purification, and biological activity of GCPs is of great significance for enhancing the added value of fish processing and promoting the sustainable development of the collagen peptide industry. However, research on the preparation process, structural characteristics, biological activity, and stability of GCPs remains inadequate. Meanwhile, studying the antioxidant stability of peptides in different food ingredients and temperature environments is of great importance for analyzing their potential as food ingredients with high antioxidant activity[3].

This study aims to explore the preparation process of GCPss through systematic experimental methods, characterize their structural features, evaluate their *in vitro* antioxidant activity and digestive stability, and further verify the experimental conclusions through synthetic peptides, thereby providing theoretical basis and technical support for the development and application of GCPs.

## Materials and Methods

### 2.1. Materials

The experimental materials and instrumentation used in this chapter were listed in Table 1.

**Table 1.** Experimental materials, instruments and equipments.

| Items | Manufacturer |
|---|---|
| Fresh grass carp swim bladder | Shanghai Pudong New Area Nanhui new town aquatic products shop |
| Alkaline protease (200u/mg) | Shanghai Yuanye Bio-Technology Co., Ltd |
| Neutral protease (50u/mg) | Shanghai Aladdin Biochemical Technology Co., Ltd |
| NaOH (AR) | Sinopharm Chemical Reagent Co., Ltd |
| HCl (AR) | Sinopharm Chemical Reagent Co., Ltd |
| NaCl | Sinopharm Chemical Reagent Co., Ltd |
| Glucose | Sinopharm Chemical Reagent Co., Ltd |
| Citric acid | Sinopharm Chemical Reagent Co., Ltd |
| pH meter | METTLER TOLEDO |
| 1,1-Diphenyl-2-picrylhydrazyl | PHYGENE |
| ABTS | PHYGENE |
| $H_2O_2$ | Guangdong Hengjian Pharmaceutical Co. LTD |
| Pyrogallol | Sinopharm Chemical Reagent Co., Ltd |
| Potassium ferricyanide | Sinopharm Chemical Reagent Co., Ltd |
| FeCl3 | Sinopharm Chemical Reagent Co., Ltd |
| FeCl2 | Sinopharm Chemical Reagent Co., Ltd |
| 1,10-Phenanthroline | Shanghai Yien Chemical Technology Co., Ltd. |
| Freeze dryer XY-FD-L1 | Shanghai XinYU Instrument Co. LTD |
| Ultrafiltration membrane | Sartorius AG |
| Sephadex G-15 | Sigma Aldrich (Shanghai) Trading Co., LTD |
| Total amino acid analyzer LA8080 | Hitachi Limited |
| Circular dichroism spectrometer | Applied Photophysics Ltd |
| H1750R-High speed refrigerated centrifuge | Xiangyi centrifuge Instrument Co., LTD |
| Visible spectrophotometer | METASH |
| LC-MSMS | Science Compass |
| Synthetic peptide | Jiangsu Jinsilui Biotechnology Co., LTD |

All other reagents used were analytically pure.

### 2.2. Preparation and Purification of GCPs

Using medium-sized fresh grass carp swim bladders as the primary raw material, GCPs were prepared through an ultrasound-assisted dual-enzyme hydrolysis method[4]. The obtained GCPs were then subjected to ultrafiltration to fractionate into GCPs with molecular weights greater than 100 kDa, 100 kDa to 5 kDa, 5 kDa to 3 kDa, and less than 3 kDa. Among these fractions, the GCPs with a molecular weight of less than 3 kDa were further purified using G-15 dextran gel chromatography. The three fractions obtained from chromatography were named GCP-I, GCP-II, and GCP-III, respectively. All sample solutions mentioned above were freeze-dried and stored at -80°C for subsequent use[5].

### *2.3. Scanning Electron Microscopy (SEM) Observation of GCPs*

A small amount of freeze-dried powder of GCPs prior to purification, as obtained in Section 2.2, was taken and ensured to be evenly dispersed on the surface of conductive double-sided adhesive tape. It was then securely fixed onto the sample stage and gently placed into an ion sputtering coater for gold sputtering to shield ion interference. Collagen peptides were observed using various magnifications.

### *2.4. Determination of Amino Acid Composition*

The amino acid composition analysis was conducted to identify the types and quantities of amino acids present in the peptide mixture, which are critical for understanding their antioxidant properties. The amino acid composition of the freeze-dried powder of GCPs obtained in Section 2.2, prior to purification, was determined with slight modifications to the method described by Shen[6].

### *2.5. Determination of in vitro Antioxidant Activity of GCPs*

GCPs fractions of various molecular weights obtained from Section 2.2, along with GCP-I, GCP-II, and GCP-III, were subjected to six *in vitro* antioxidant activity assays: ABTS radical scavenging assay[7], DPPH radical scavenging assay[8], hydroxyl radical scavenging assay[9], superoxide anion radical scavenging assay[10], total reducing power determination[11], and ferrous ion chelating assay[12]. Based on the comprehensive experimental results, the primary samples for subsequent experiments were selected.

### *2.6. In vitro Simulated Gastrointestinal Digestion Experiments*

A simulated gastrointestinal digestion system was established with minor adjustments based on the method described by Minekus M et al[13].

#### 2.6.1. *In vitro* Simulated Oral Digestion

5.00 g of washed, impurity-removed, room temperature-dried, and chopped grass carp swim bladder, as well as freeze-dried powder of **GCPs** obtained from previous experiments without purification, and GCP-I, GCP-II, and GCP-III obtained through ultrafiltration and chromatography, were placed in 50 mL centrifuge tubes separately. 4 mL of SSF solution at pH 7 (containing 15.1 mmol/L KCl, 3.7 mmol/L $KH_2PO_4$, 3.6 mmol/L $NaHCO_3$, 0.15 mmol/L $MgCl_2(H_2O)_6$, and 0.06 mmol/L $(NH_4)_2CO_3$) and 25 μL of $CaCl_2$ were added to the centrifuge tubes. The tubes were then oscillated at a constant temperature of 37°C for 5 minutes and stored at -80°C.

#### 2.6.2. *In vitro* Simulated Gastric Digestion

8 mL of SGF solution at pH 3 (containing 6.9 mmol/L KCl, 0.9 mmol/L $KH_2PO_4$, 25 mmol/L $NaHCO_3$, 47.2 mmol/L NaCl, 0.12 mmol/L $MgCl_2(H_2O)_6$, and 0.5 mmol/L $(NH_4)_2CO_3$), 4000 U/mL of pepsin, and 5 μL of $CaCl_2$ were added into the samples that had completed oral digestion. The samples were oscillated at a constant temperature of 37°C for 2 hours and stored at -80°C.

#### 2.6.3. *In vitro* Simulated Intestinal Digestion

16 mL of SIF solution at pH 7 (containing 6.8 mmol/L KCl, 0.8 mmol/L $KH_2PO_4$, 85 mmol/L $NaHCO_3$, 38.4 mmol/L NaCl, and 0.33 mmol/L $MgCl_2(H_2O)_6$), 2 mg/mL of pancreatic enzymes, 2 mg/mL of porcine bile salts, and 25 μL of $CaCl_2$ were added to the samples that had completed gastric digestion. The samples were oscillated at a constant temperature of 37°C for 4 hours. After oscillation, the enzymes were inactivated through boiling water bath, and the samples were freeze-dried and stored at -80°C for future use.

### *2.7. Circular Dichroism Analysis*

Based on the results of previous experiments on antioxidant activity, GCP-II was initially selected for secondary structure determination. A certain amount of purified GCP-II freeze-dried powder and its digestive products were weighed and prepared into 0.5 mg/mL solutions for circular dichroism analysis. The scanning wavelength range was set from 190 nm to 250 nm, with a frequency of 50 nm/min and a response time of 1 second.

### *2.8. Amino Acid Composition Analysis*

Amino acid analysis was performed on the GCP-II digestion products. The method for amino acid composition analysis was the same as in Section 2.4.

### *2.9. Determination of Antioxidant Stability of GCPs*

To investigate the influence of processing environments and food ingredients on the antioxidant activity stability of GCPs, GCP-II was used as the main sample to determine the stability of its antioxidant activity in different temperatures, concentrations, and food component environments. Based on the experimental results in Section 2.5, selected antioxidant activity indicators were used as criteria for assessing antioxidant stability, providing a comprehensive evaluation of the antioxidant stability of GCPs.

#### 2.9.1. Effect of Temperature on the Antioxidant Stability of GCPs

GCPs freeze-dried powder was dissolved in ultrapure water to prepare a 1 mg/mL sample solution as control group A0. The same concentration of sample solution was placed in water baths at 4°C, 50°C, and 100°C for 1 hour, respectively. Their antioxidant activities were measured and compared with the control group. The calculation formula is as follows:

$$\text{Antioxidant Stability}(\%) = \frac{A}{A_0} \times 100 \quad (1)$$

Where A represents the antioxidant activity of GCPs after different treatments, and $A_0$ represents the antioxidant activity of untreated GCPs.

#### 2.9.2. Effect of NaCl on the Antioxidant Stability of GCPs

GCPs freeze-dried powder was dissolved in ultrapure water to prepare a 1 mg/mL sample solution as control group A0. NaCl was added to the same concentration of sample solution to adjust the NaCl percentage concentration to 1%, 2%, and 4%, respectively. The mixtures were homogenized and allowed to stand at room temperature for 1 hour. Their antioxidant activities were measured and compared with the control group.

#### 2.9.3. Effect of Glucose on the Antioxidant Stability of GCPs

GCPs freeze-dried powder was dissolved in ultrapure water to prepare a 1 mg/mL sample solution as control group A0. Fructose was added to the same concentration of sample solution to adjust the fructose percentage concentration to 1%, 4%, and 7%, respectively. The mixtures were homogenized and allowed to stand at room temperature for 1 hour. Their antioxidant activities were measured and compared with the control group.

#### 2.9.4. Effect of Citric Acid on the Antioxidant Stability of GCPs

GCPs freeze-dried powder was dissolved in ultrapure water to prepare a 1 mg/mL sample solution as control group A0. Citric acid was added to the same concentration of sample solution to adjust the citric acid percentage concentration to 0.04%, 0.12%, and 0.20%, respectively. The mixtures were homogenized and allowed to stand at room temperature for 1 hour. Their antioxidant activities were measured and compared with the control group.

2.9.5. Study on the Antioxidant Stability of Digestive Products of GCPs

The freeze-dried powder of GCP-II digestive products at the same concentration was used to investigate the antioxidant stability after digestion, and the antioxidant activities were measured according to the method in Section 2.5 of this study.

### *2.10. Structural Identification of GCPs*

Based on previous experiments, the sample with the highest comprehensive antioxidant activity was selected for sequence identification and subsequent experiments. Mass spectrometry identification of the sample was conducted by Scientific Compass. The experimental procedure was as follows: the freeze-dried sample was redissolved, and 10 kDa ultrafiltration was performed to remove impurities and desalt the sample before mass spectrometry detection.

The raw mass spectrometry files after the experiment were searched using MaxQuant 1.5.5.1 for peptide sequence identification and quantitative analysis.

### *2.11. Solid-Phase Synthesis of Peptides*

The selected peptides were synthesized by Jiangsu GenScript Biotech Corporation.

### *2.12. Prediction of Physicochemical Properties of Peptides*

The methods for predicting the physicochemical properties of the synthesized peptides were shown in Table 2.

**Table 2.** Methods for predicting the physical and chemical properties of peptides.

| Items | Forecasting Methods |
| --- | --- |
| Water solubility | Innovagen |
| Toxicity assessment | ToxinPred |
| Molecular weight and isoelectric point | Expasy-compute |
| Net charge and hydrophobicity | Pepdraw |

### *2.13. Verification of Antioxidant Activity of Peptides*

The ABTS radical scavenging assay, DPPH radical scavenging assay, hydroxyl radical scavenging assay, superoxide anion radical scavenging assay, and ferrous ion chelating assay were all conducted according to the methods outlined in Section 2.5.

### *2.14. Determination of Synergistic Effects of Peptides*

Based on the results of the antioxidant activity verification experiments, two or more synthetic peptides were mixed in equal amounts to prepare samples of a certain concentration, and antioxidant activity experiments were performed. The results obtained from the synergistic experiments were considered as measured values, while the weighted average of the antioxidant capacities of each individual peptide served as the corresponding actual value. By comparing the theoretical values with the measured values, the synergistic or antagonistic relationships among the selected peptides in terms of antioxidant and whitening activities were determined.

## 3. Results and Discussion

### *3.1. Structural Characterization of GCPs*

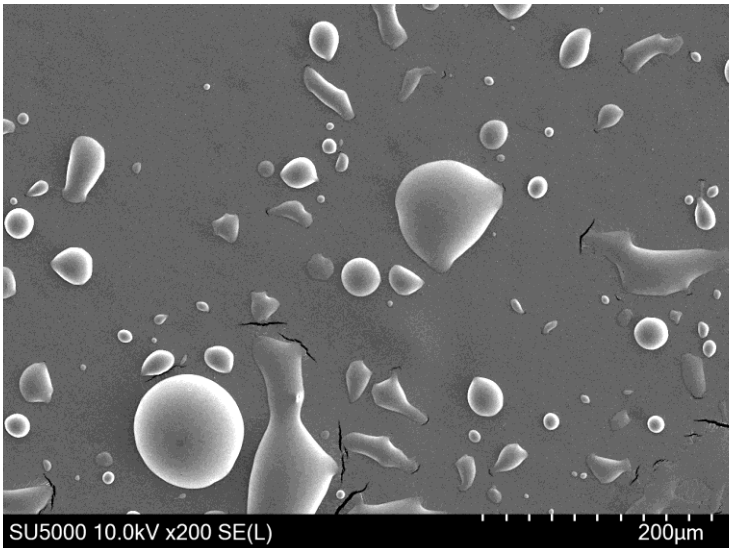


**Figure 1. The Scanning Electron Microscopy of GCPs**.

Scanning Electron Microscopy (SEM) was capable of observing the surface morphology and microstructure of samples with high resolution and magnification. For collagen peptides, SEM technology provides intuitive visualization of their fibrous structure, particle size, and surface morphology.

Figure 1 (SU5000 model, accelerating voltage of 10.0 kV, magnification ×200, secondary electron imaging mode) revealed the surface morphological characteristics of GCPs. Based on the scale bar (200 μm), the GCPs prepared in previous experiments exhibited the smooth and delicate morphology, and no obvious fracture or impurity phase was observed in the structure, indicating high sample purity and no significant structural damage was introduced during the preparation. This was similar to the characteristics observed in pearl peptides by Feng et al.[14], laying a foundation for subsequent studies on separation, purification, and other physicochemical properties.

### *3.2. Amino Acid Composition Analysis of GCPs*

To gain a more specific understanding of the types and quantities of all amino acids constituting the active peptides from GCPs, an LA8080 amino acid analyzer was used to conduct amino acid composition analysis on the unpurified GCPs enzymatic hydrolysate. 17 amino acids were detected, including 6 essential amino acids and 7 hydrophobic amino acids. The total amino acid content in GCPs was 69.092 g/100 g, with hydrophobic amino acids accounting for 20.97 g/100 g and essential amino acids for humans accounting for 24.084 g/100 g. Phenylalanine, the most abundant amino acid in GCPs, serves as a precursor for synthesizing other compounds with antioxidant activity, such as phenylacetic acid and phenyllactic acid, thus having an impact on the antioxidant activity of substances[15]. Other hydrophobic amino acids, such as Ala, Leu, and Ile, have also been found to play a role in antioxidant activity[16]. Additionally, studies have shown that arginine possesses antioxidant properties, and its content in GCPs was relatively high[17], at 5.270 g/100 g. Therefore, it was speculated that GCPs has potential antioxidant functions.

**Table 3.** Analysis of amino acid component.

| **Items** | **Content（g/100 g）** |
| --- | --- |
| Gly | 19.632 |
| Phe$^{*\#}$ | 8.584 |
| Lys$^{*}$ | 7.157 |

| | |
|---|---|
| Arg | 5.370 |
| Leu*# | 4.454 |
| Glu | 4.429 |
| Tyr | 4.298 |
| Ala# | 2.965 |
| Asp | 2.936 |
| Ser | 2.084 |
| Ile*# | 2.012 |
| Thr | 1.815 |
| Val*# | 1.533 |
| Pro# | 1.078 |
| His | 0.391 |
| Met*# | 0.344 |
| Cys | 0.010 |

Note: * indicates essential amino acids for humans, # indicates hydrophobic amino acids.

### *3.3. Measurement of Antioxidant Activity in vitro of GCPs (GCPs)*

The antioxidant activity of GCPs with different molecular weights and concentrations was determined experimentally, as shown in Figure 2. "VC" refers to Vitamin C, used as a positive control in the antioxidant assays.

To explore the impact of concentration on the antioxidant activity of the samples, the freeze-dried peptide powders of four gradient molecular weights obtained after ultrafiltration were first dissolved in ultrapure water and then adjusted to concentrations of 1 mg/mL, 5 mg/mL, and 10 mg/mL. Antioxidant activity assays were then conducted.

Analysis revealed that the purified GCPs exhibited considerable antioxidant activity *in vitro*. The antioxidant activity of GCPs increased with concentration. The effect of molecular weight on its antioxidant activity was unstable, but in most cases, GCPs with lower molecular weight had stronger antioxidant capacity.

As shown in Figure 3, GCP-II exhibited the highest antioxidant activity *in vitro*. Its ABTS radical scavenging activity, superoxide anion scavenging activity, and ferrous ion chelating ability were relatively good, with maximum values reaching 86.4%, 72.05%, and 83.62%, respectively. In comparison, its DPPH radical scavenging activity was weaker, and its total reducing power at a concentration of 10 mg/mL was only 65.13%.

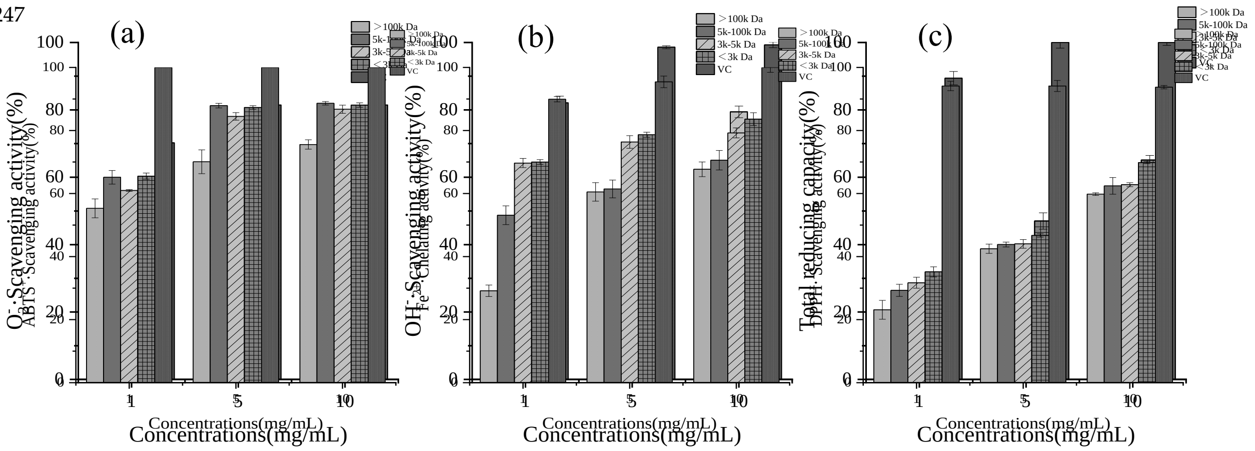


**Figure 2.** $ABTS^{+}$·scavenging activity (a), DPPH· scavenging activity (b), $OH^{-}$·scavenging activity (c) of GCPs, $O_2^{-}$·scavenging activity (d), total reducing capacity (e), $Fe^{2+}$·chelating activity (f) with different molecular weight and concentration.

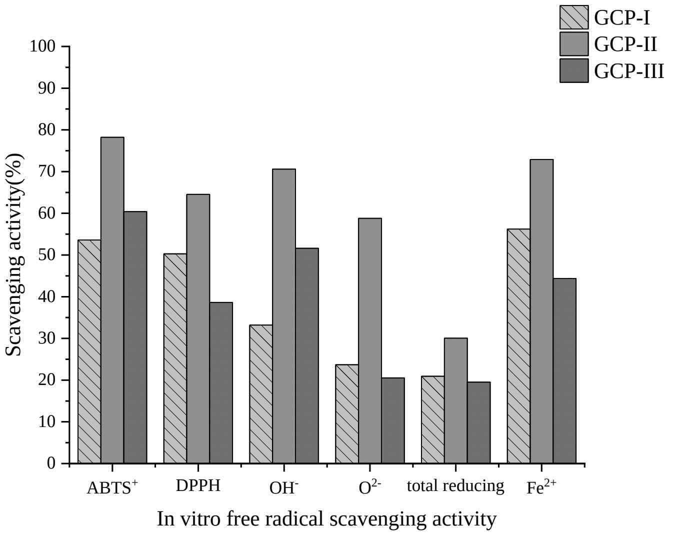


Figure 3. *In vitro* free radical scavenging activity of GCP-I, GCP-II and GCP-III

### *3.4. Secondary Structure Analysis of GCPs and Digestive Products*

The secondary structures of collagen primarily include $\alpha$-helix, β-sheet, β-turn, and random coil[18]. During digestion, these structures were broken down into smaller peptide fragments and amino acids due to the action of enzymes, resulting in changes to the secondary structures. As shown in Figure 4, GCP-II exhibited a distinct positive absorption peak near a wavelength of 190 nm and notable negative absorption peaks at 208 nm and near 230 nm, indicative of a typical $\alpha$-helix structure[19]. Its β-sheet structure was present near 208 nm, manifested as a negative absorption peak, while the positive absorption peak at 222 nm suggests that GCP-II also possesses β-sheet structure[20][21]. In contrast, the digestive products of GCP-II exhibited a significant negative absorption peak near 200 nm, indicating that the original secondary structures have been disrupted by digestion, leading to the formation of a disordered structure of random coils. Additionally, the negative absorption peaks of GCP-II digestive products in the range of 260-310 nm further confirmed the generation of random coil structures.

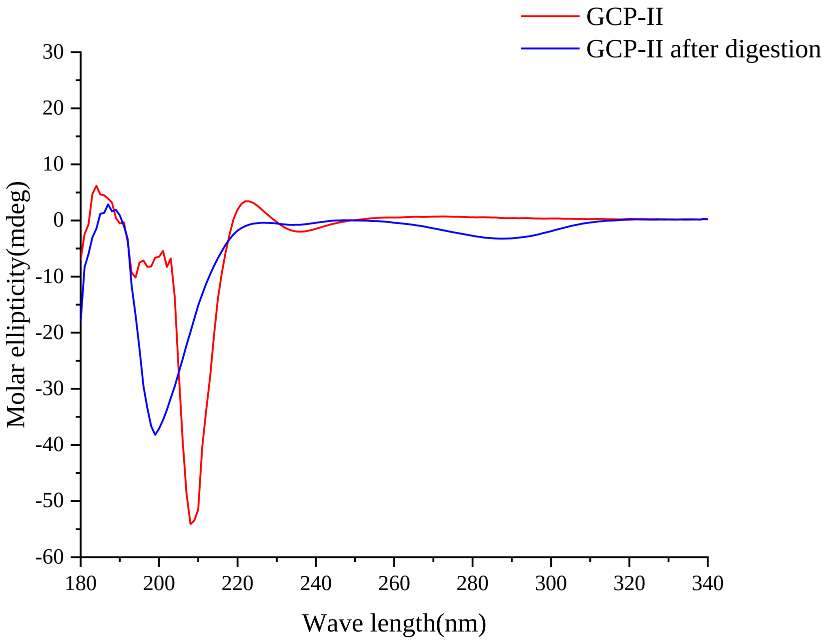


**Figure 4.** Changes in the secondary structure of GCP-II before and after digestion.

### *3.5. Analysis of Amino Acid Composition of Digestive Products of GCPs*

To investigate the impact of simulated *in vitro* digestion experiments on the amino acid composition of GCPs, GCP-II, which exhibited the highest overall antioxidant activity in previous experiments, and its digestive products were selected as samples for further research and discussion. As shown in Table 4, glycine and arginine were abundant in GCP-II, with concentrations reaching 7.032 g/100 g and 2.819 g/100 g, respectively. These two amino acids were often considered to have a strong correlation with the antioxidant activity of substances, indicating that the notable antioxidant capacity of GCP-II. During the simulated *in vitro* digestion experiments, the amino acid composition of the peptides may undergo a series of changes, primarily influenced by the enzymatic action during the simulated digestion process, the digestion conditions, and the intrinsic properties of the peptides themselves. Following a series of digestive reactions, the total amino acid content in GCP-II decreased to 1.448 g/100 g, with nearly all detected amino acids showing substantial reductions in their concentrations. The contents of glycine, arginine, and hydrophobic amino acids such as phenylalanine and valine, which were known to influence antioxidant activity, also decreased significantly. Therefore, it was speculated that the *in vitro* antioxidant capacity of the digestive products of GCP-II will be markedly reduced after digestion treatment.

**Table 4.** Analysis of amino acid component of GCP-II.

before and after digestion（g/100 g）

| Items | GCP-II | GCP-II after Digestion |
|---|---|---|
| Gly | 7.032 | 0.169 |
| Lys* | 5.459 | 0.058 |
| Arg | 2.819 | 0.311 |
| Ser | 1.797 | 0.098 |
| Asp | 1.135 | 0.129 |
| Glu | 0.855 | 0.153 |
| Phe*# | 0.815 | 0.104 |
| Thr | 0.641 | 0.071 |

| | | |
|---|---|---|
| Val*# | 0.639 | 0.075 |
| Leu*# | 0.487 | 0.094 |
| Gcu# | 0.419 | 0.068 |
| Ile*# | 0.304 | 0.049 |
| Met*# | 0.207 | 0.011 |
| His | 0.125 | 0.019 |
| Tyr | 0.025 | 0.038 |
| Pro# | 0.009 | 0.001 |
| Toatal | 22.768 | 1.448 |

### *3.6. Impact of Simulated in vitro Digestion on the Antioxidant Activity of GCPs*

Studies have indicated that during simulated gastrointestinal digestion, the action of digestive enzymes may lead to the cleavage or hydrolysis of peptides, altering their structure and potentially affecting their antioxidant activity[22]. As shown in Figure 5, after undergoing the complete simulated *in vitro* digestion process, all active components isolated through chromatography exhibited varying degrees of decline in their antioxidant activity. Specifically, the DPPH radical scavenging rates dropped from their original values of 50.29%, 64.55%, and 38.63% to 9.68%, 12.43%, and 11.33% respectively, representing the largest decrease among the tested indices. It was speculated that during gastrointestinal digestion, GCPs undergoes further enzymatic hydrolysis into smaller peptide fragments and amino acids, and the antioxidant activity of these newly generated small molecules was significantly lower than that of the original active fragments of GCPs. Observations from gastrointestinal digestion experiments revealed that GCPs exhibited relatively stable ABTS radical scavenging activity and hydroxyl radical scavenging activity during the digestion process. Based on these findings, it was hypothesized that certain antioxidant functions of GCPs possess good digestive stability.

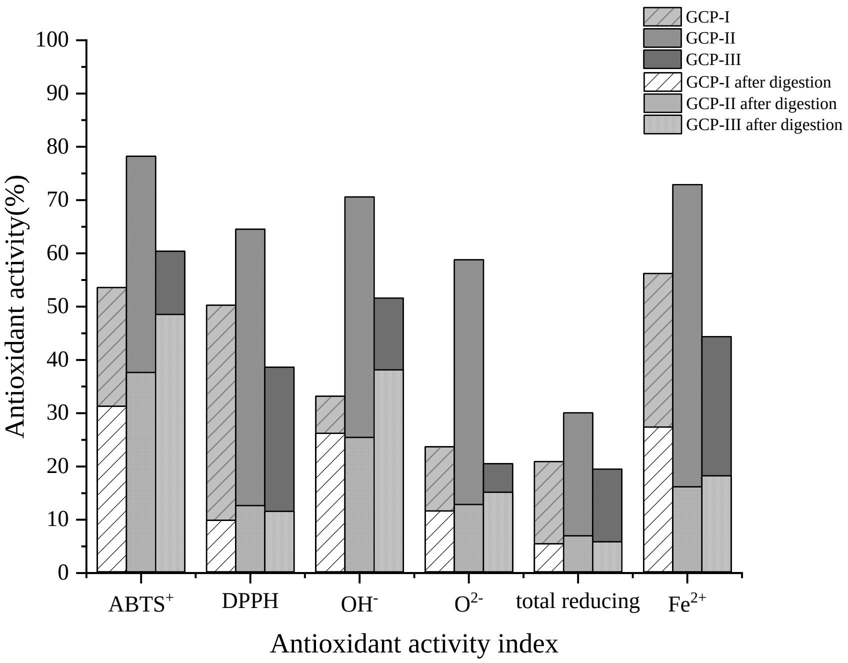


**Figure 5.** *In vitro* free radical scavenging activity of GCP-I, **GCP-II and GCP-III before and after digestion.**

### *3.7. Antioxidant Stability Assessment Results of GCP-II*

The determination of the antioxidant stability of peptides was of great significance for evaluating their antioxidant capacity, guiding applications, exploring mechanisms of action, and facilitating research and development, as well as optimization. Based on the results of previous experiments, GCP-II was selected as the main object of antioxidant stability experiment for its great antioxidant activity.

### 3.7.1. Impact of Temperature on the Antioxidant Stability of GCP-II

As shown in Figure 6, GCP-II generally exhibits good antioxidant stability at 4 °C, maintaining over 90% activity. The antioxidant property most affected by temperature was its hydroxyl radical scavenging activity, which decreases significantly as the temperature rises. When heated to 100 °C, the retention rate of hydroxyl radical scavenging activity was 79.32%. Both the ferrous ion chelating ability and superoxide anion scavenging capacity of GCP-II display the considerable thermal stability, with antioxidant retention rates still above 90% at 100 °C. Overall, the antioxidant activity of GCPs demonstrates good thermal stability in the range of 4 °C to 100 °C.

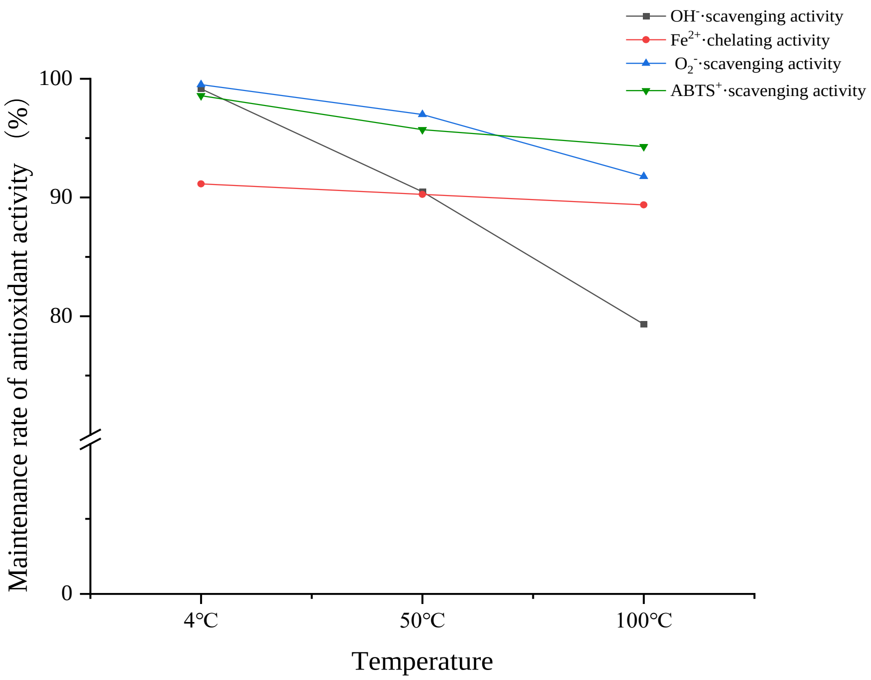


**Figure 6.** Maintenance rate of antioxidant activity of GCP-II at different temperatures.

### 3.7.2. Impact of Food Ingredient Components on the Antioxidant Stability of GCP-II

The effects of common food ingredient components on the antioxidant stability of GCP-II were illustrated in Figure 7. Among them, the ferrous ion chelating ability of GCP-II was largely unaffected by various concentrations and types of food ingredients, remaining stable at over 95%. In contrast to its thermal stability, the superoxide anion scavenging capacity of GCP-II was more significantly influenced by food components. Both NaCl and citric acid can significantly reduce its stability when their concentrations reach 4% and 0.2%, respectively. Therefore, during the processing and storage of GCP-II, it was advisable to avoid high-concentration salt environments and acidic conditions to the greatest extent possible.

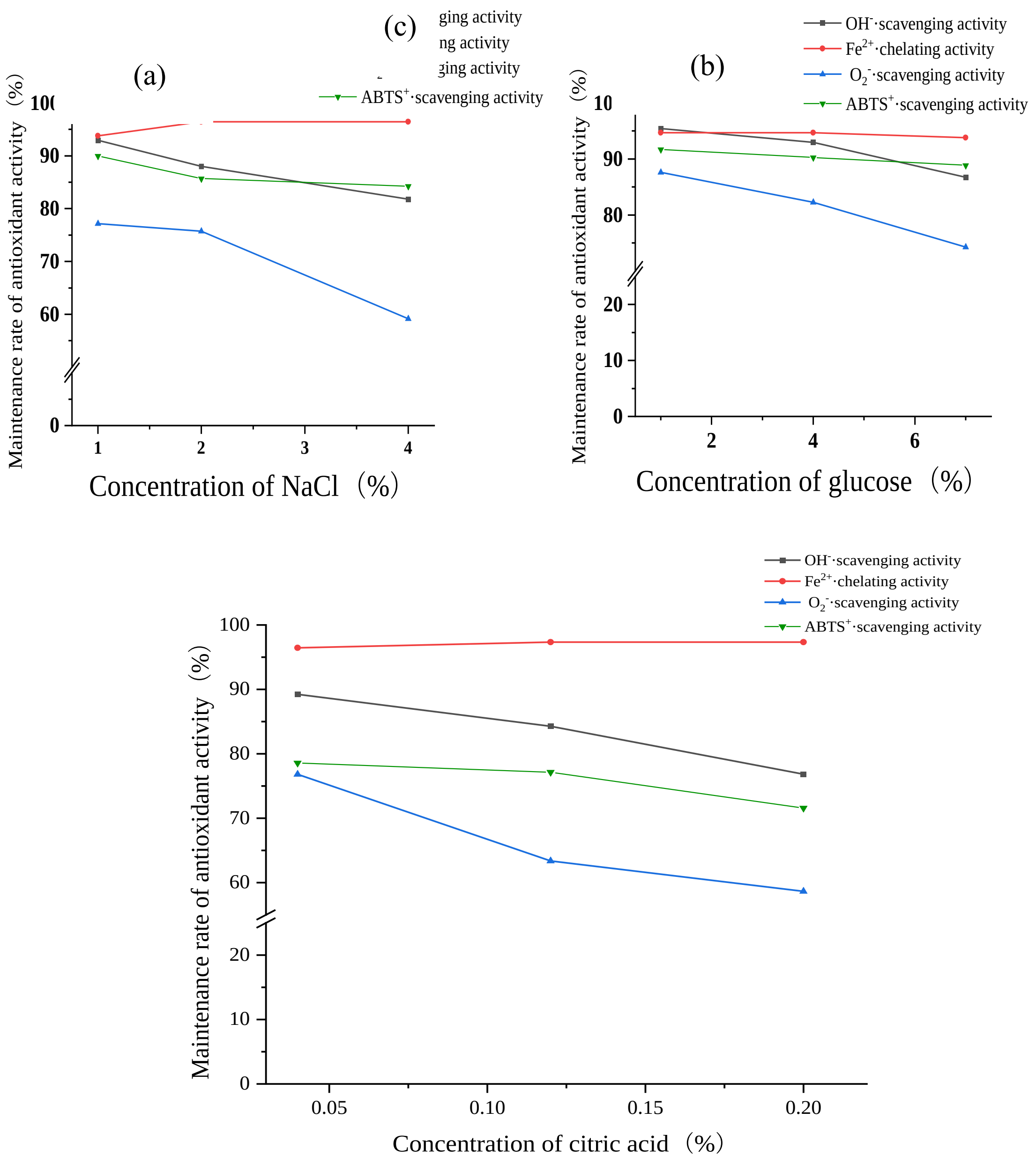


**Figure 7.** Effects of different concentrations of NaCl (a), glucose (b), and citric acid (c) on the antioxidant activity of GCP-II.

### *3.8. Structural Identification of GCP-II*

Structural identification of GCP-II was conducted using LC-MSMS, resulting in the detection of 464 sequences. Among these, 137 sequences were composed of 3 to 7 amino acids, and 327 sequences were composed of 8 to 25 amino acids. A total of 295 sequences had a confidence score of 90 or above. The Base Peak Chromatogram (BPC) of GCP-II was shown in Figure 8 (a) below, while its Total Ion Chromatogram (TIC) was presented in Figure 8 (b). By analyzing the BPC spectrum, researchers can gain insights into the distribution of different components in the sample and their relative abundances[23]. This analysis aids in identifying the peptide components within the sample and provides an initial assessment of their concentrations. In contrast to the BPC, the TIC reflects the chromatogram of all ions in the sample, providing comprehensive information about the overall

distribution and abundance of all ions in the sample[24]. It thus offers a more holistic view of the chromatographic distribution of all ions within the sample.

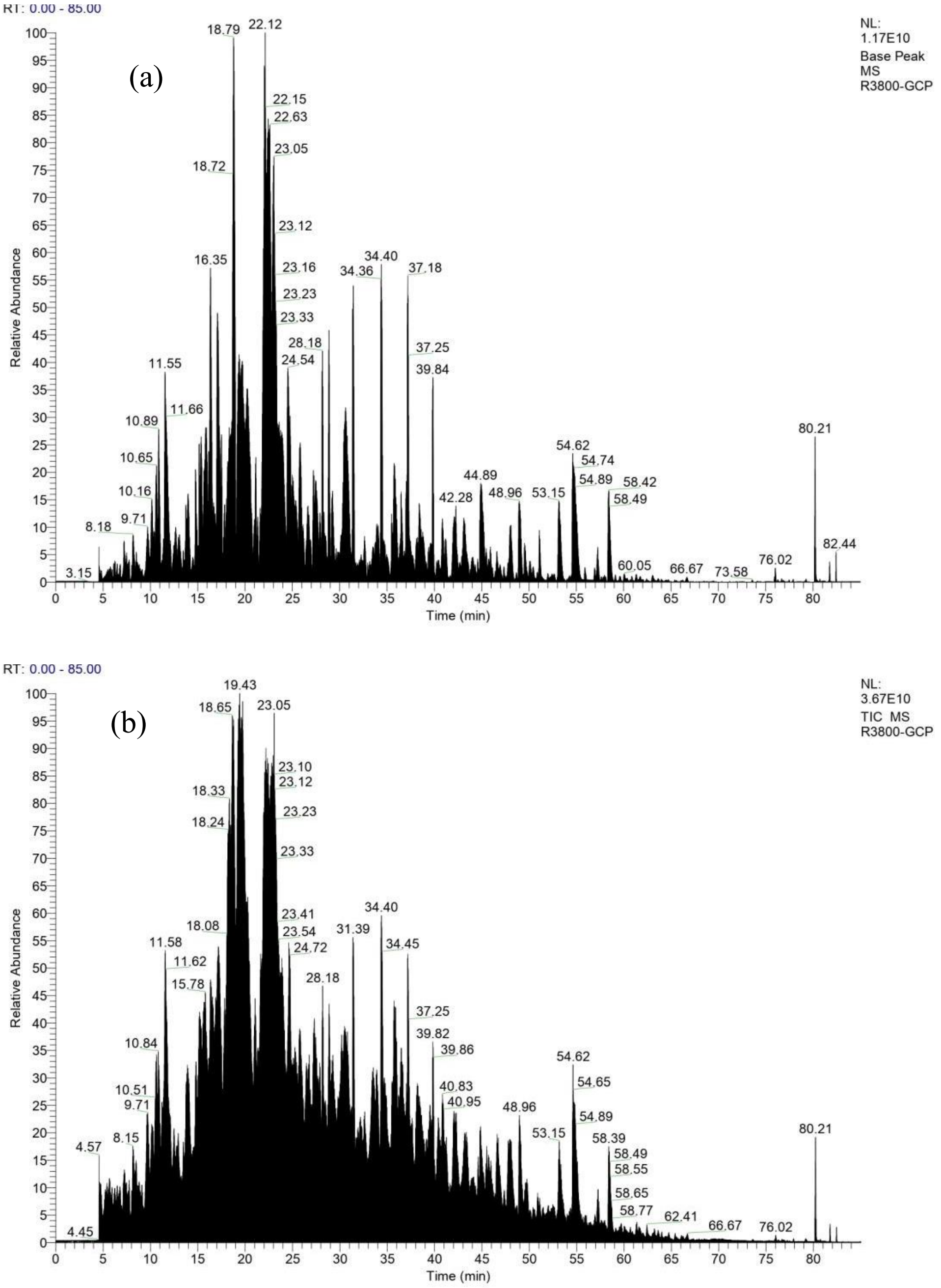


**Figure 8.** Base Peak Chromatogram (a) and Total Ion Chromatogram (b) of GCP-II.

### *3.9. Peptide Selection and Prediction of Physicochemical Properties*

Seven active fragments with potential antioxidant activity were identified and selected through comparison with the BIOPEP database. Their specific sequences and predictions of physicochemical properties were as follows.

**Table 5.** Prediction of physicochemical properties of peptides.

| Sequence | Water sol-ubi-lity | Hydroph-obi-city kcal/mol | Toxicity assess-ment | Isoelectric point | MW (molecular weight) | Net charge |
|---|---|---|---|---|---|---|

| | | | | | | |
|---|---|---|---|---|---|---|
| EKAPDPFRHF | High | 19.47 | non-toxic | 6.85 | 1243.39 | 0 |
| QGPPGPPGPS | High | 13.28 | / | 5.52 | 889.96 | 0 |
| GERGPPGPM | High | 16.54 | non-toxic | 6.00 | 897.02 | 0 |
| DGSYNIGQR | High | 15.90 | non-toxic | 6.84 | 1009.04 | 0 |
| GILTLKYPI | Lower | 6.79 | non-toxic | 8.59 | 1017.28 | 1 |
| VLSLYASGRTT | Lower | 9.11 | non-toxic | 8.72 | 1167.33 | 1 |
| ILTERGYSFVTT | Lower | 10.45 | non-toxic | 6.00 | 1386.57 | 0 |

After conducting mass spectrometry analysis on these seven peptides using LC-MSMS, the mass spectra were presented as follows:

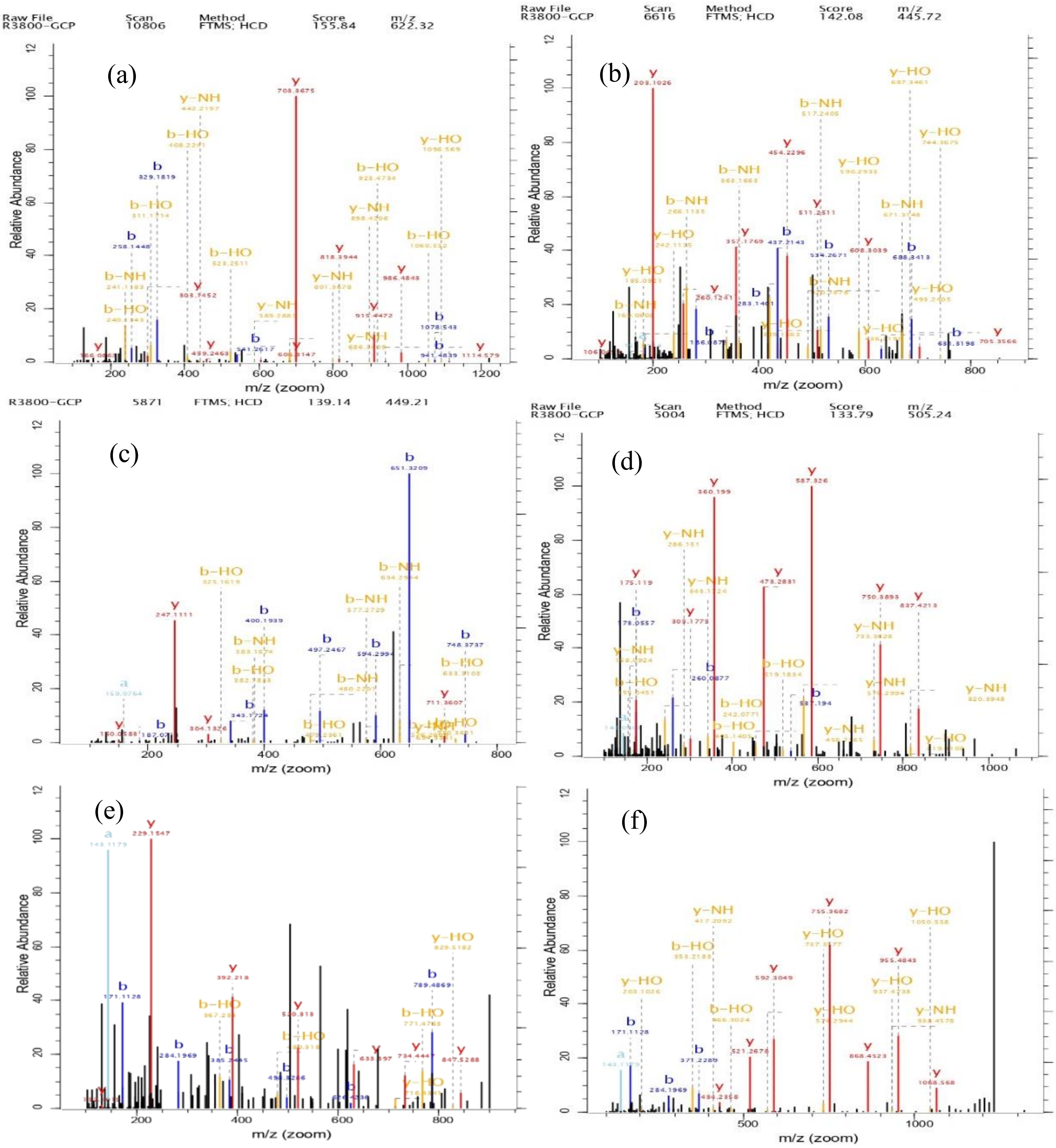

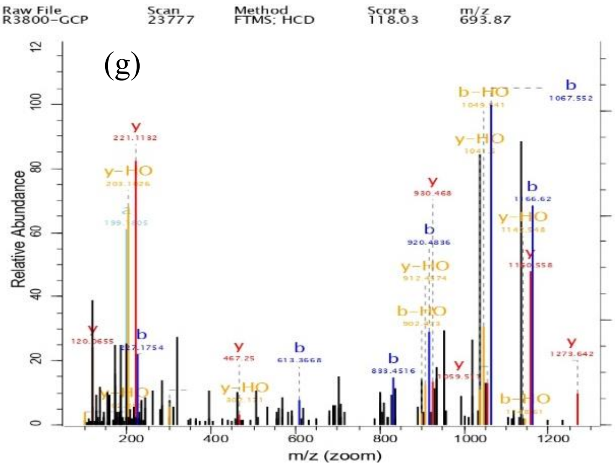


Figure 9. The mass spectrum of EKAPDPFRHF (a), QGPPGPPGPS (b), GERGPPGPM (c), DGSYNIGQR(d), GILTLKYPI(e), VLSLYASGRTT(f), ILTERGYSFVTT(g)

After screening, seven peptide sequences, namely EKAPDPFRHF, GILTLKYPI, GERGPPGPM, ILTERGYSFVTT, QGPPGPPGPS, VLSLYASGRTT, and DGSYNIGQR, were selected for polypeptide synthesis. The screening process primarily considered the functional activities of the sequences recorded in BIOPEP-UWM, along with the comprehensive scores from mass spectrometry analysis, the number of times they were tested, and their peak intensities. The comprehensive score of a peptide sequence indicates its reliability; the seven selected peptide sequences all had scores above 100, suggesting high reliability. Peak intensity represents the signal strength of the peptide sequence, which can be used for quantitative analysis and as a criterion for peptide selection. Among them, four peptide sequences were predicted to have good water solubility, at least six were non-toxic, and all seven contain amino acid sequences that have been documented to have high antioxidant activity, such as LY, RHF, LK, TERGY, GPP, LKYPI, YNI, and YA. Therefore, it was speculated that these seven peptide sequences possess high antioxidant activity.

**Table 6.** Information on selected peptides.

| Items | Sequence | Length | Score | Frequency of Detection | Peak Intensity |
|---|---|---|---|---|---|
| Peptide 1 | EKAPDPFRHF | 10 | 155.84 | 1 | 3260300000 |
| Peptide 2 | GILTLKYPI | 9 | 129.68 | 2 | 1151900000 |
| Peptide 3 | GERGPPGPM | 9 | 139.14 | 1 | 804920000 |
| Peptide 4 | ILTERGYSFVTT | 12 | 118.03 | 1 | 581870000 |
| Peptide 5 | QGPPGPPGPS | 10 | 142.08 | 1 | 359000000 |
| Peptide 6 | VLSLYASGRTT | 11 | 122.13 | 3 | 347310000 |
| Peptide 7 | DGSYNIGQR | 9 | 133.79 | 1 | 313090000 |

### *3.10. Verification of Physicochemical Properties of Synthesized Peptides*

The seven peptide sequences from Section 3.9 were synthesized and named as peptide 1, peptide 2, peptide 3, peptide 4, peptide 5, peptide 6, and peptide 7, respectively, from top to bottom according to Table 6.

#### 3.10.1. Purity Information of Synthesized Peptides

As observed from the HPLC chromatograms, the purity of all seven synthesized peptides reached above 95%, making them suitable for subsequent research on antioxidant activity and tyrosinase inhibition.

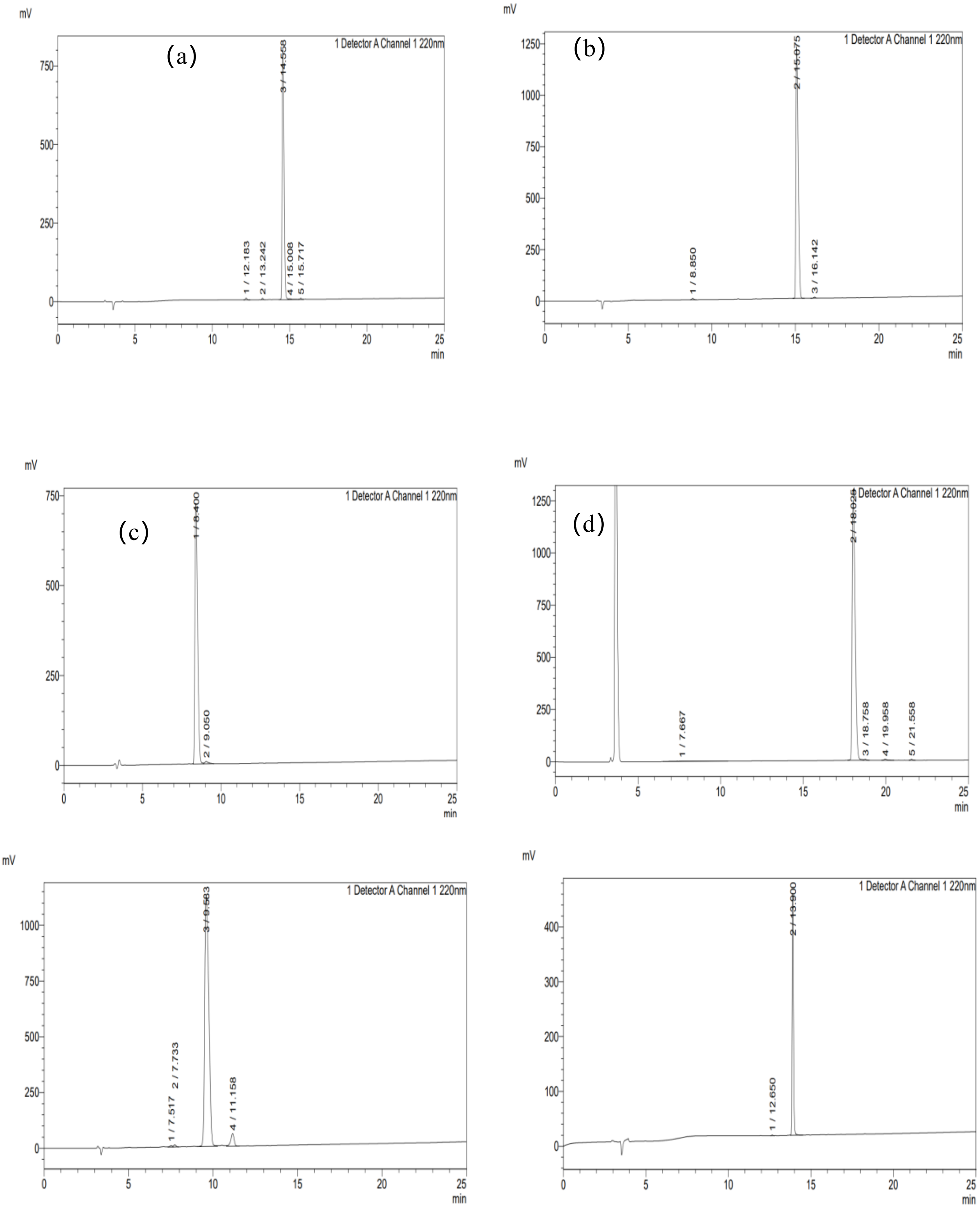

(a)
(b)
(c)
(d)
mV
min
1 Detector A Channel 1 220nm
1 / 12.183
2 / 13.242
3 / 14.558
4 / 15.008
5 / 15.717
1 / 8.850
2 / 15.075
3 / 16.142
1 / 8.400
2 / 9.050
1 / 7.667
2 / 18.025
3 / 18.758
4 / 19.958
5 / 21.558
1 / 7.517
2 / 7.733
3 / 9.583
4 / 11.158
1 / 12.650
2 / 13.900

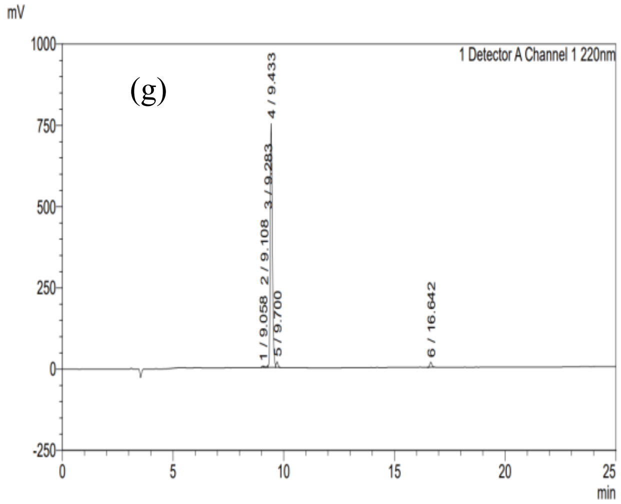


**Figure 10.** HPLC plots of peptide 1 (a), peptide 2 (b), peptide 3 (c), peptide 4 (d),peptides 5 (e), peptides 6 (f), and peptides 7 (g).

3.10.2. Verification of Mass Spectrometry Information for Synthesized Peptides

To further validate the purity of the synthesized peptides for subsequent experiments, mass spectrometry analysis was conducted by Jiangsu GenScript Biotechnology Corporation. The parameters were set as follows: nebulizer gas flow rate of 1.5 L/min; CDL temperature of 250 °C; drying gas flow rate of 5 L/min; blocking temperature of 200 °C; T-flow rate of 0.2 mL/min; and solvent B consisting of 50% $H_2O$ + 50% MeOH. Under these parameters, the mass spectrometry results for the seven peptides were presented below.

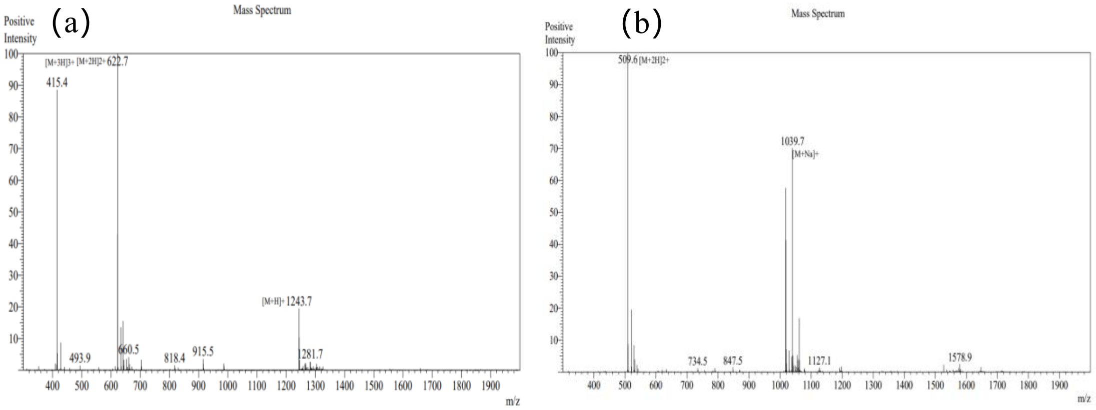

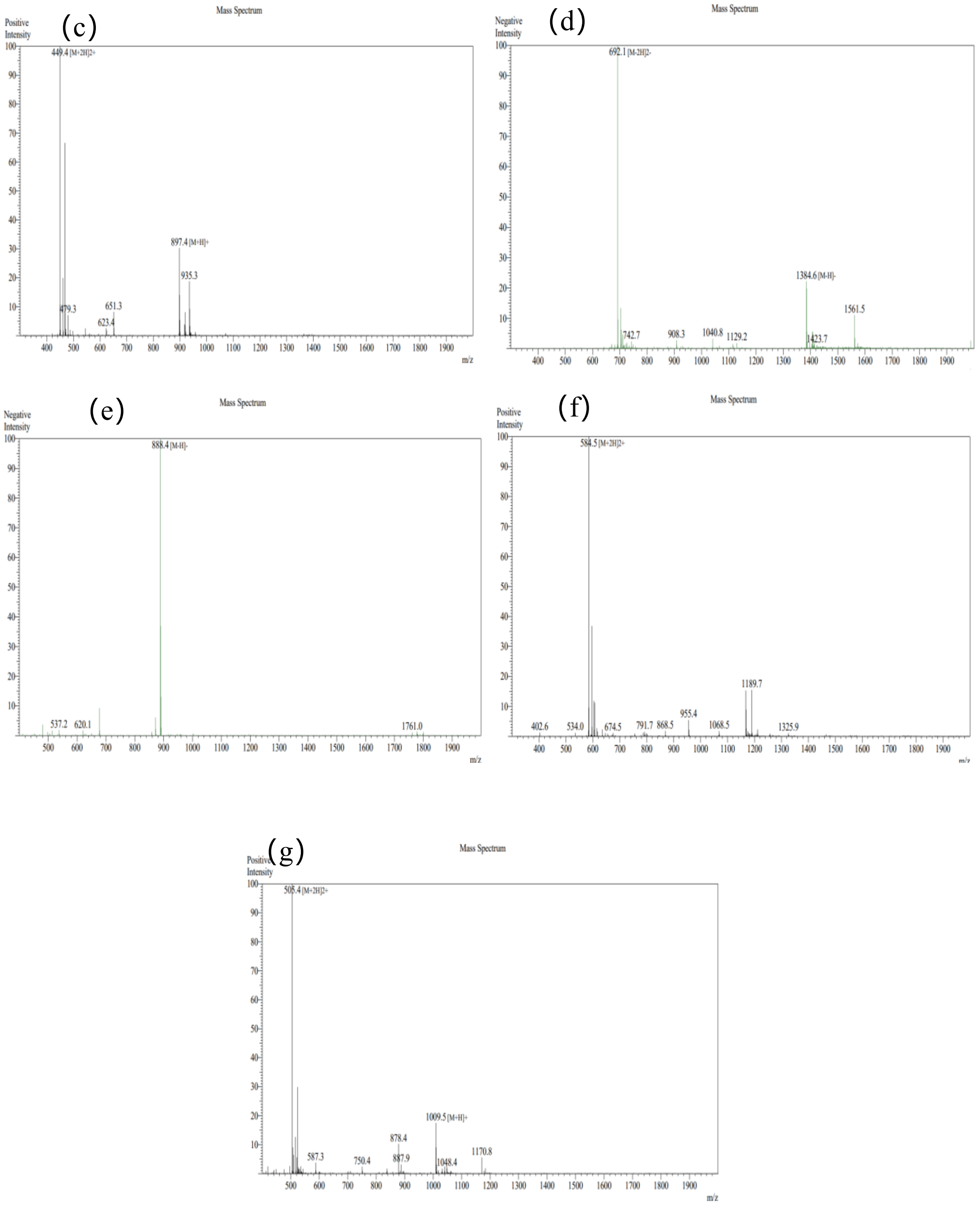


**Figure 11.** Mass spectrum of peptide 1 (a), peptide 2 (b), peptide 3 (c), peptide 4 (d),peptides 5 (e), peptides 6 (f), and peptides 7 (g).

### 3.10.3. Verification of Antioxidant Activity of Synthetic Peptides

#### 3.10.3.1 Determination of ABTS Radical Scavenging Activity

As shown in Figure 12, the overall ABTS radical scavenging activity of the seven synthetic peptide segments was relatively good. Among them, peptide 6 exhibits the highest activity, with a scavenging rate of 63.31% at a concentration of 1 mg/mL. In addition to peptide 6, peptides 1, 4, and 7 also demonstrate considerable ABTS radical scavenging abilities, all exceeding 40%. Notably, these three peptides contain the aromatic amino acid Y (Tyr). Studies have indicated that all phenolic hydroxyl groups on tyrosine significantly impact the antioxidant activity of active substances[25]. These hydroxyl groups can act as

hydrogen donors to capture radicals, and the phenoxy radicals released after a series of reactions exhibit strong stability, thereby enhancing the ABTS radical scavenging capacity of these active substances[26]. Furthermore, research has found that H (His) also exerts a certain influence on the antioxidant activity of active fragments[27][28].

However, in comparison, the antioxidant activity of the peptide GQCHV was substantially improved. For the peptide GQCH, both activities were substantially enhanced. When C was replaced with A, both activities were significantly reduced, and the removal of H resulted in the loss of antioxidant activity [29]. This indicates that the aromatic amino acids Tyr (Y) and His (H) have a beneficial effect on the ABTS radical scavenging activity of substances[30].

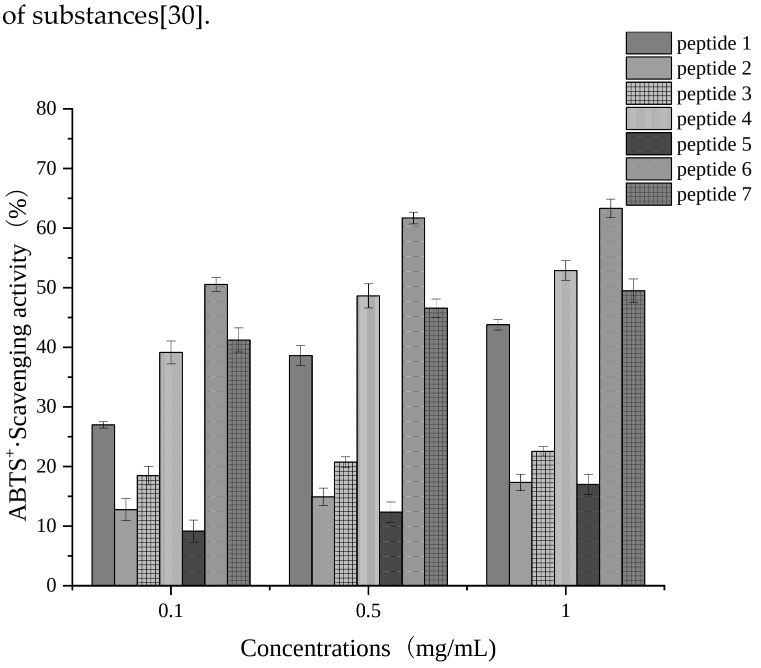


Figure 12. ABTS+·scavenging activity at various concentrationsfor each synthetic peptide.

3.10.3.2 Determination of DPPH Radical Scavenging Activity

The DPPH radical scavenging activity of each active fragment increased steadily with increasing concentration, but the overall scavenging activity was lower than that observed for ABTS radicals, which was similar to the trend observed for GCPs. As shown in Figure 13, peptide 5 exhibited the relatively strongest DPPH radical scavenging activity, reaching 49.98%, followed by peptides 1 and 3 with 37.73% and 35.41% respectively. The higher scavenging activity of peptide 5 may be attributed to the significant impact of proline at the third position from its N-terminus on its antioxidant properties, which was similar to the results obtained by Zhu[31] for the DPPH radical scavenging activity of short peptides synthesized from oligopeptide sequences in the enzymatic hydrolysis products of grass carp fish meat.

Furthermore, H. et al.[32] studied and analyzed the DPPH radical scavenging activity of GL-9 (GASRHWTFL) and found that it exhibited high scavenging activity with a trend similar to that of glutathione. Another study indicated that the sequence WRKKDPLND obtained from digestive hydrolysates of halfbeak anchovy possesses relatively high DPPH radical scavenging ability[33]. Gupta A et al.[34] extracted a peptide with the sequence VKEAMAPK from cheddar cheese, which exhibited DPPH radical scavenging activity comparable to that of commercial antioxidants such as BHA, t-BHQ, and Ferulic acid.

Taken together, the content of proline, histidine, glycine, and tryptophan has a significant impact on the DPPH radical scavenging capacity of active fragments.

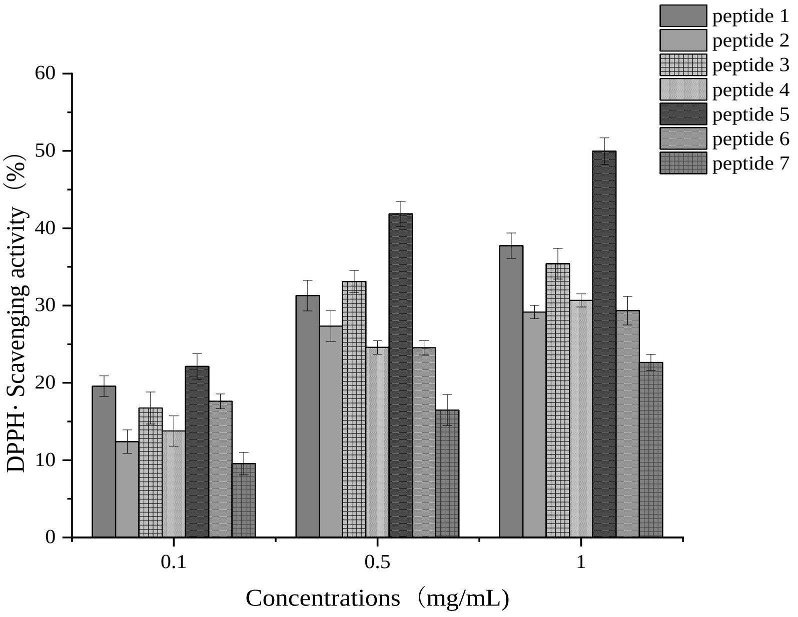


**Figure 13.** DPPH· scavenging activity at various concentrationsfor each synthetic peptide.

3.10.3.3 Determination of Hydroxyl Radical Scavenging Activity

In the experiment assessing hydroxyl radical scavenging activity, peptides 3 and 6 demonstrated strong scavenging capabilities, with scavenging rates of 55.98% and 55.42% respectively at a concentration of 1 mg/mL. Overall, the hydroxyl radical scavenging activity of all synthetic peptides was considerable, with scavenging rates almost exceeding 30% at the highest concentration and maintaining above 10% even at the lowest concentration of 0.1 mg/mL. Notably, all seven peptides synthesized in our experiment contained hydrophobic amino acids with an overall high content, which may account for their high hydroxyl radical scavenging activity[35].

Additionally, studies have found that glycine (Gly) has a significant influence on the antioxidant activity of substances. Peptide 3, which has a high glycine content[36], also exhibited high hydroxyl radical scavenging activity, aligning with these findings.

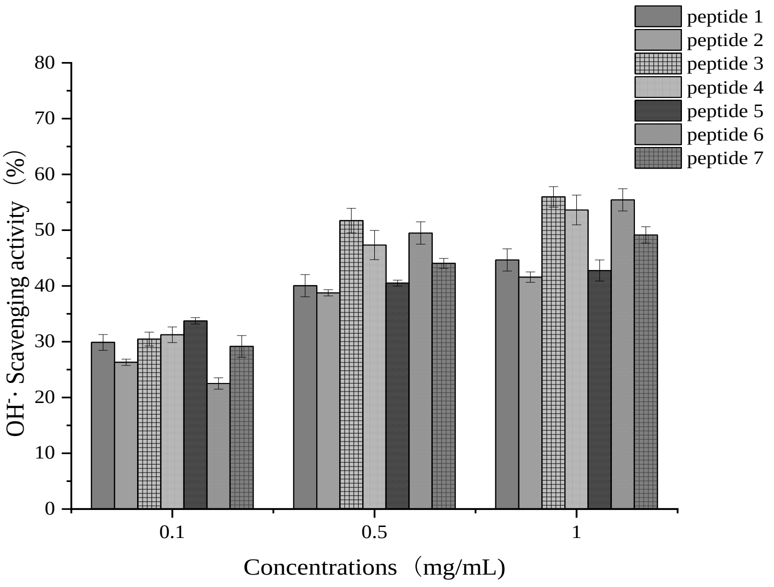


**Figure 14.** OH-· scavenging activity at various concentrations for each synthetic peptide.

3.10.3.4 Determination of Superoxide Anion Scavenging Activity

In the superoxide anion scavenging experiments, the synthetic peptides generally exhibited strong scavenging capabilities. Among them, peptide 1 demonstrated the highest scavenging activity, reaching 42.14%, followed by peptides 6 and 7 with scavenging rates of 38.61% and 36.09% respectively. Studies have speculated that aspartic acid present in peptide sequences may significantly contribute to their superoxide anion scavenging ability. Compared to other synthetic fragments, peptides 1 and 7 contain aspartic acid, aligning with the research direction inferred in these studies[37]. Yang et al.[38] found that the sequence SVDGKEDLIW exhibited the highest overall antioxidant activity and demonstrated strong superoxide anion scavenging ability in mouse oxidative stress response experiments. Notably, this sequence also contains aspartic acid (D).

Additionally, studies have indicated that valine (Val) or leucine (Leu) at the N-terminus can positively affect the antioxidant activity of active fragments. Peptide 6, which has valine at its N-terminus and was rich in hydrophobic amino acids, was speculated to possess high superoxide anion scavenging ability due to these characteristics[39].

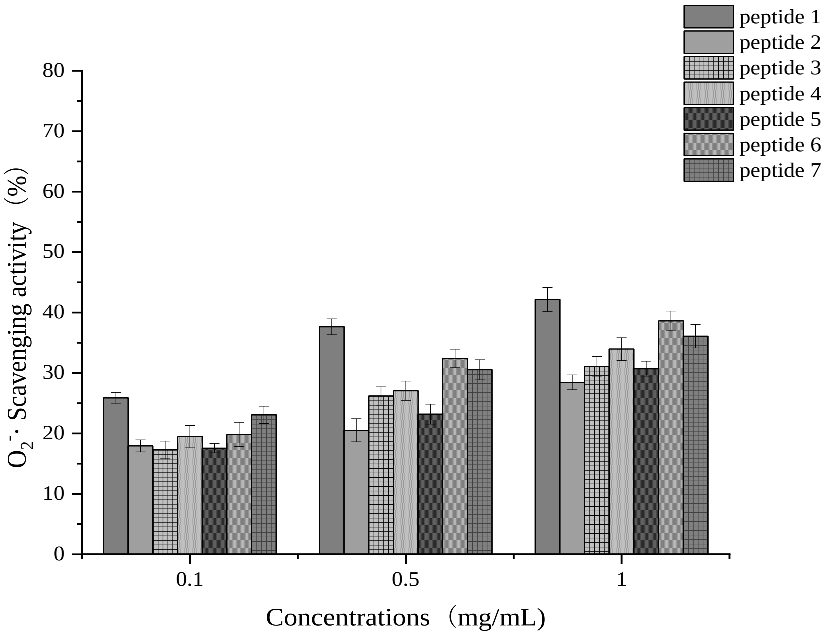


**Figure 15.** $O_2^{-}\cdot$ scavenging activity at various concentrations.for each synthetic peptide.

3.10.3.5 Determination of Ferrous Ion Chelating Ability

As illustrated in Figure 16, peptides 7 and 3 exhibit strong ferrous ion chelating abilities, while peptide 2 demonstrates the weakest metal chelating ability, with a chelating rate of only 16.81% at a concentration of 1 mg/mL. At the same concentration, peptide 7 achieves a chelating rate of 44.72%, and peptide 3 reaches 40.59%. Both of these peptides contain two acidic amino acids, glutamic acid (G) or aspartic acid (D). Studies have indicated that acidic amino acid residues can chelate transition metal ions, thereby achieving antioxidant effects[40]. It was speculated that the presence of these two acidic amino acids may contribute to the strong metal chelating abilities of these two peptides[41][42]. Similarly, peptide 7 not only contained abundant hydrophobic amino acids but also was enriched with acidic amino acids such as glutamine (Q), tyrosine (Y), tryptophan (W), and asparagine (N). This might be one of the reasons for the good antioxidant activity of peptide 7.

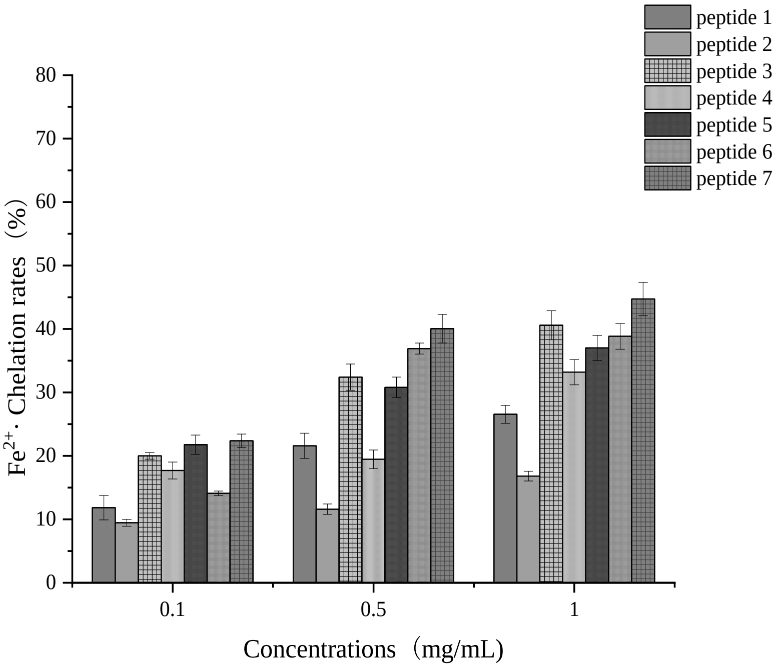


**Figure 16.** $Fe^{2+}\cdot$ chelation rates at various concentrations for each synthetic peptide.

## *3.11. Determination of Synergistic Effects of Peptides*

Based on the experimental results from Section 3.10.3, peptides 3 and 6 were selected for evaluation of their synergistic effects. Considering the antioxidant activities of these two peptides, DPPH radical scavenging ability, hydroxyl radical scavenging ability, and superoxide anion scavenging ability were chosen as indicators for assessing their synergistic effects.

As shown in Figure 17, with increasing concentrations, these two peptides demonstrated a certain degree of synergistic effect overall. In the ABTS radical scavenging experiment and superoxide anion scavenging experiment, the actual values for peptides 3 and 6 at concentration gradients of 0.1 to 1 mg/mL were higher than the theoretical values. Similarly, in the DPPH radical scavenging experiment and hydroxyl radical scavenging experiment, peptides 3 and 6 also exhibited notable synergistic effects, with the most pronounced effect observed at a concentration of 0.1 mg/mL. At this concentration, the DPPH radical scavenging ability of peptides 3 and 6 increased from a theoretical value of 20.65% to an actual value of 21.53%. Under the same concentration, their hydroxyl radical scavenging ability also increased from 24.96% to 27.12%.

In a word, it was concluded that peptides 3 and 6 exhibited a certain degree of synergism in antioxidant activity experiments at different concentrations.

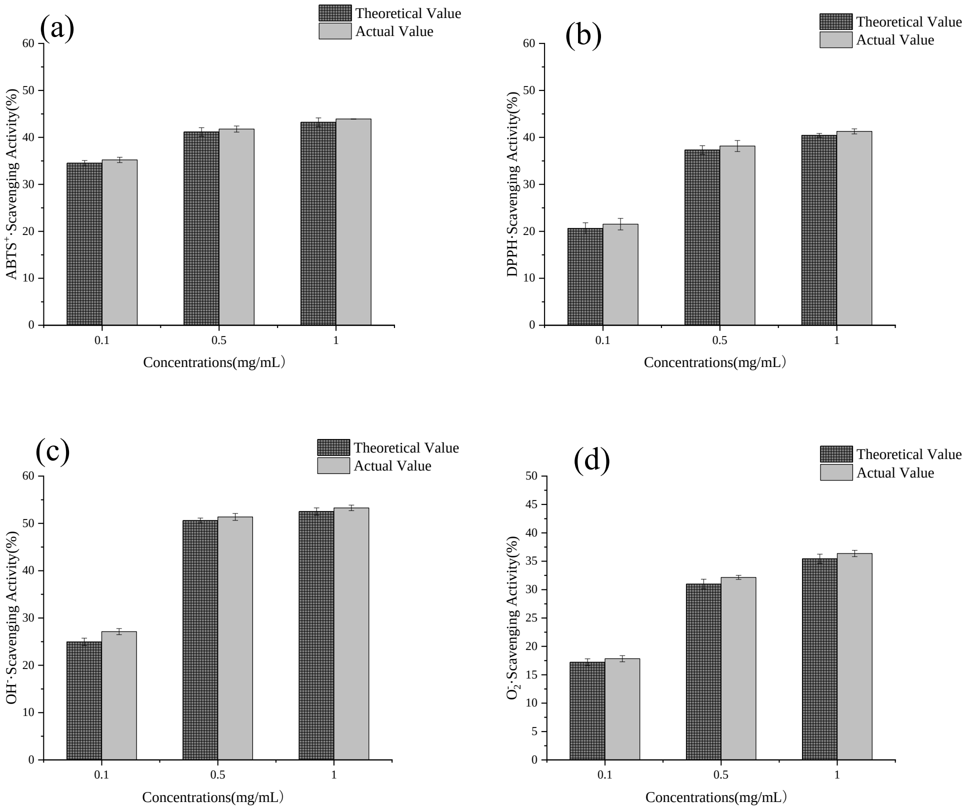


**Figure 17.** Analysis of synergistic effect of synthetic peptides.

## 4. Conclusion

Through systematic experimental design and data analysis, this study conducted an in-depth exploration of the preparation, purification, structural characterization, antioxidant activity, and stability of collagen peptides derived from grass carp swim bladders (GCPs). Then, ultrafiltration and chromatography were performed on the preliminarily prepared GCPs, and the samples obtained by chromatography were named GCP-I, GCP-II and GCP-III, respectively. After analysis of the results of previous antioxidant experiments, GCP-II was selected as the main sample for the synthetic peptide experiments. The sequence of GCP-II was analyzed, from which 7 bioactive fragments with high antioxidant potential were selected and synthesized. Their sequences were EKAPDPFRHF, GILTLKYPI, GERGPPGPM, ILTERGYSFVTT, QGPPGPPGPS, VLSLYASGRTT and DGSYNIGQR and were named as peptide 1, peptide 2, peptide 3, peptide 4, peptide 5, peptide 6 and peptide 7, respectively. Among them, the two sequences with the highest comprehensive antioxidant activity were peptide 3 and peptide 6, and these two peptides showed synergistic effects in many antioxidant experiments. The research results indicated that GCPs, as a polypeptide with considerable antioxidant activity, maintains high antioxidant activity under the influence of different environments and concentrations of food ingredients, thus demonstrating good overall stability.

However, there were still some limitations in this study. For instance, although peptide segments with high antioxidant activity have been screened out, further in-depth research is needed to investigate their specific antioxidant mechanisms. Additionally, the safety and effectiveness of GCPs in practical applications need to be verified through more animal experiments and clinical trials.

**Author Contributions:** Conceptualization, Suxin Li and Jinhui Gu; Data curation, Suxin Li, Weiqiang Qiu and Wenzheng Shi; Formal analysis, Suxin Li, Jinhui Gu and Yiyi Liu; Funding acquisition, Wenzheng Shi; Investigation, Suxin Li and Yiyi Liu; Methodology, Suxin Li, Jinhui Gu and Yiyi Liu; Project administration, Suxin Li; Resources, Weiqiang Qiu and Wenzheng Shi; Software, Suxin Li; Supervision, Weiqiang Qiu and Wenzheng Shi; Validation, Suxin Li and Jinhui Gu; Visualization, Suxin Li and Jinhui Gu; Writing – original draft, Suxin Li and Wenzheng Shi; Writing – review & editing, Suxin Li. All authors have read and agreed to the published version of the manuscript.

**Data Availability Statement:** The original contributions presented in the study are included in the article, further inquiries can be directed to the corresponding author.

**Acknowledgments:** This study was supported by National Key Research and Development Program of China (grant number 2019YFD0902003) and SciTech Funding by CSPFTZ Lingang Special Area Marine Biomedical Innovation Platform.

**Conflicts of Interest:** The authors declare no conflicts of interest.

## Reference:

1. Zhang, Z.; Chen, W.; Xu, T.; et al. Exploring the Indicator Gut Microbiota Taxa in Grass Carp (Ctenopharyngodon idella): Correlations with Growth Rates. Aquaculture **2025**, 599, 742080. https://doi.org/10.1016/j.aquaculture.2024.742080
2. Xie, X.-D.; Feng, L.; Jiang, W.-D.; Wu, P.; Liu, Y.; Ren, H.-M.; Jin, X.-W.; Zhang, R.-N.; Zhou, X.-Q. From Antioxidant to Muscle Enhancer: Resveratrol's Role in Grass Carp (Ctenopharyngodon idella) Nutrition. Aquacult. Rep. **2024**, 39, 102499. https://doi.org/10.1016/j.aqrep.2024.102499
3. Zeng, X.; Zhou, X.-Q.; Jiang, W.-D.; Wu, P.; Liu, Y.; Ma, Y.-B.; Tang, L.; Li, S.-W.; Kuang, S.-Y.; Feng, L. Histidine Promotes Muscle Growth and Protein Deposition in Grass Carp (Ctenopharyngodon idella): Evidence from In Vivo and In Vitro Models. Food Biosci. **2024**, 62, 105537. https://doi.org/10.1016/j.fbio.2024.105537
4. Suxin Li et al., Aquaculture and Fisheries,https://doi.org/10.1016/j.aaf.**2023**.12.009
5. Cristina Torres-Fuentes, María del Mar Contreras, Isidra Recio, Manuel Alaiz, Javier Vioque,Identification and characterization of antioxidant peptides from chickpea protein hydrolysates,Food Chemistry,Volume 180,**2015**,Pages 194-202,ISSN 0308-8146.
6. Shen, C.H.; Yang, J.; Zhang, Y.H., et al. Optimization of Double-Enzyme Hydrolysis Process of Pigeon Breast Meat and Evaluation of Its Antioxidant Activity. Food & Machinery. **2023**, 39(04), 163-169.
7. Manujaya W. Jayamanna Mohottige, Angéla Juhász, Mitchell G. Nye-Wood, Katherine A. Farquharson, Utpal Bose, Michelle L. Colgrave, Beyond nutrition: Exploring immune proteins, bioactive peptides, and allergens in cow and Arabian camel milk, Food Chemistry,Volume 467**,2025**,142471,ISSN 0308-8146.
8. Tao Zi, Zhao Zhengang. Construction of lemongrass essential oil microemulsion and its antioxidant activity analysis [J]. Modern Food Science and Technology, **2018**,34(10): 156-164.
9. G.R. Zhao, H.M. Zhang, T.X. Ye, Z.J. Xiang, Y.J. Yuan, Z.X. Guo and L.B. Zhao, Food Chem. Toxicol., 46, 73 (**2008**).
10. Li N, Shen X R, Liu Y M, et al. Isolation, characterization, and radiation protection of Sipunculusnudus L. polysaccharide. International Journal of Biological Macromolecules, **2016**, 83:288- 296.
11. Xiang Annie, Xu Shuang, Ju Hongmei et al. Purification, structural characterization and antioxidant activ-ity of selenoprotein from Cyanobacteria chinensis [J]. Journal of Northwest Agricultural Sciences, **2019**,31(03):299-309.
12. Zhang L, Hu Y, Duan X Y, et al. Characterization and antioxidant activities of polysaccharides from thirteen boletus mushrooms. International Journal of Biological Macromolecules, **2018**, 113: 1- 7.
13. Minekus M，Alminger M，Alvito P，et al. A standardised static *in vitro* digestion method suitable for food - an international consensus[J]. Food & Function，**2014**，5(6): 1113-1124.
14. FENG Simin, WANG Jing, WANG Yuying, et al. Preparation and Properties of Pearl Peptide Chelated Calcium[J]. Scie nce and Technology of Food Industry, **2022**, 43(1): 119−126. (in Chinese with English abstract). doi: 10.13386/j.issn1002-0306.2021040018.
15. LIU H, SUN H N, ZHANG M, et al. Production, identification and characterization of antioxidant pep-tides from potato protein by energy-divergent and gathered ultrasound assisted enzymatic hydrolysis[J]. Food Chemistry, **2023**, 405: 134873.
16. Wang, L.; Li, Z.Y.; Fan, X.; Zhang, T.; Wang, H.; Ye, K.P. Novel antioxidant peptides from bovine blood: Purification, identification and mechanism of action. LWT. **2024**, 205, 116499. https://doi.org/10.1016/j.lwt.2024.116499.
17. Ren, L.K.; Yang, Y.; Ma, C.M.; Fan, J.; Bian, X.; Liu, B.X.; Wang, D.F.; Zhu, P.Y.; Fu, Y.; Zhang, N. Identification and in silico analysis of novel antioxidant peptides in broken rice protein hydrolysate and its cytoprotective effect against $H_2O_2$-induced 2BS cell model. Food Research International. **2022**, 162(Pt B), 112108. https://doi.org/10.1016/j.foodres.2022.112108.
18. Mi Chunxiao, Yang Lu, Yang Heqi, et al. Tilapia skin antioxidant peptide structure-activity parsing and microcapsule s teady research [J/OL]. Food industry science and technology, 1-28 ,**2024**. HTTP: / / https://doi.org/10.13386/j.issn1002-030 6.2024060441.
19. Yuan Jiang, Mingdi Zhang, Songyi Lin, Sheng Cheng,Contribution of specific amino acid and secondary structure to the antioxidant property of corn gluten proteins,Food Research International,Volume 105,**2018**,Pages 836-844,ISSN 0963-9969,

20. Shen Zhang, Yichu Shan, Shurong Zhang, Zhigang Sui, Lihua Zhang, Zhen Liang, Yukui Zhang,NIPTL-Novo: Non-isobaric peptide termini labeling assisted peptide de novo sequencing,Journal of Proteomics,Volume 154,**2017**,Pages 40-48,ISSN 1874-3919.
21. Fang Shiqi, Ren Qingxi, Zhou Zhilei, et al. The separation and purification of rice wine peptide research progress and its functions [J/OL]. Food and fermentation industry, 1-13 ,**2024**. https://doi.org/10.13995/j.cnki.11-1802/ts.040716.
22. García Fillería, S.; Nardo, A.E.; Paulino, M.; Tironi, V. Peptides Derived from the Gastrointestinal Digestion of Amarant h 11S Globulin: Structure and Antioxidant Functionality. Food Chem. Mol. Sci. **2021**, 3, 100053. https://doi.org/10.1016/j.fochms.2021.100053.
23. Wang S.Study on memory improving effect, absorption metabolism and mechanism of action of walnut peptide [D]. South China university of technology, **2023**. DOI: 10.27151 /, dc nki. Ghnlu. 2023.000209.
24. Yoshida H, Komiya A, Ohtsuki R, et al. Relationship of hyaluronan and HYBID (KIAA1199) expression with roughness parameters of photoaged skin in Caucasian women[J]. Skin Research and Technology, **2018**, 24(4): 562-569.
25. Liu Hui, LIU Fang. Study on the whitening effect and antioxidant effect of iso-astilbe [J]. Daily Chemical Industry (Chinese and English),**2024**,54(11):1368-1374.
26. Hernández-Ledesma B, Dávalos A, Bartolomé B, et al. Preparation of antioxidant enzymatic hydrolysates from α-lactalbumin and β-lactoglobulin. Identification of active peptides by HPLC-MS/MS[J]. Journal of Agricultural and Food Chemistry, **2005**, 53(3): 588-593.
27. Liu Hui, LIU Fang. Study on the whitening effect and antioxidant effect of iso-astilbe [J]. Daily Chemical Industry (Chinese and English),**2024**,54(11):1368-1374.
28. Hannah Sunde, Kate Ryder, Alaa El-Din A. Bekhit, Alan Carne,Analysis of peptides in a sheep beta lactoglobulin hydrolysate as a model to evaluate the effect of peptide amino acid sequence on bioactivity,Food Chemistry,Volume 365,**2021**,130346,ISSN 0308-8146.
29. Liu Hui, LIU Fang. Study on the whitening effect and antioxidant effect of iso-astilbe [J]. Daily Chemical Industry (Chinese and English),**2024**,54(11):1368-1374.
30. Shabestarian, H., Asoodeh, A., Homayouni-Tabrizi, M. and Hossein-Nejad-Ariani, H. (**2017**), Antioxidant and Angiotensin I Converting Enzyme (ACE) Inhibitory Properties of GL-9 Peptide. Journal of Food Processing and Preservation, 41: e12838.
31. Aursuwanna, T.; Noitang, S.; Sangtanoo, P.; Srimongkol, P.; Saisavoey, T.; Puthong, S.; Reamtong, O.; Karnchanatat, A. Investigating the cellular antioxidant and anti-inflammatory effects of the novel peptides in lingzhi mushrooms. Heliyon. **2022**, 8(10), e11067. https://doi.org/10.1016/j.heliyon.2022.e11067.
32. Rodríguez, M.; Tironi, V.A. Chemical and cell antioxidant activity of amaranth flour and beverage after simulated gastrointestinal digestion. Role of peptides. Food Research International. **2023**, 173(Pt 2), 113410. https://doi.org/10.1016/j.foodres.2023.113410.
33. Yu, X.; Chen, Y.N.; Qi, Z.G.; Chen, Q.; Cao, Y.J.; Kong, Q.S. Preparation and identification of a novel peptide with high antioxidant activity from corn gluten meal. Food Chemistry. **2023**, 424, 136389. https://doi.org/10.1016/j.foodchem.2023.136389.
34. Yang, R.W.; Li, X.F.; Lin, S.Y.; Zhang, Z.M.; Chen, F. Identification of novel peptides from 3 to 10 kDa pine nut (Pinus koraiensis) meal protein, with an exploration of the relationship between their antioxidant activities and secondary structure. Food Chemistry. **2017**, 219, 311-320. https://doi.org/10.1016/j.foodchem.2016.09.163
35. Peixin Wang, Jiawen Zhang, Yibin Tang, Zhigang Zhang, Yi Zhang, Jiamiao Hu. Purification and characterization of antioxidant peptides from hairtail surimi hydrolysates and their effects on beef color stability. J Food Sci. **2021**; 86: 2898–2909.
36. Lobo, V., Patil, A., Phatak, A., & Chandra, N. (**2010**). Free radicals, antioxidants and functional foods: Impact on human health. Pharmacognosy Reviews, 4(8), 118–126.
37. Khammuang, S.; Sarnthima, R.; Sanachai, K. Purification and identification of novel antioxidant peptides from silkworm pupae (Bombyx mori) protein hydrolysate and molecular docking study. Biocatalysis and Agricultural Biotechnology. **2022**, 42, 102367. https://doi.org/10.1016/j.bcab.2022.102367.
38. Wang, J.N.; Yang, G.; Li, H.F.; Zhang, T.; Sun, D.; Lu, W.P.; Zhang, W.J.; Wang, Y.H.; Ma, M.; Cao, X.F.; Zhang, B.; Guo, Y.C. Preparation and identification of novel antioxidant peptides from camel bone protein. Food Chemistry. **2023**, 424, 136253. https://doi.org/10.1016/j.foodchem.2023.136253.
39. V.P. Shanmugam, S. Kapila, T. Kemgang Sonfack, R. Kapila,Antioxidative peptide derived from enzymatic digestion of buffalo casein,International Dairy Journal,Volume 42,**2015**,Pages 1-5,ISSN 0958-6946.
40. Shi Y N, Kovacs-Nolan J, Jiang B, et al. Antioxidant activity of enzymatic hydrolysates from eggshell membrane proteins and its protective capacity in human intestinal epithelial Caco-2 cells. Journal of Functional Food, **2014**, 10: 35-45.
41. Krobthong, Sucheewin; Yingchutrakul, Yodying (**2020**). Identification and enhancement of antioxidant P1-peptide isolated from <i>Ganoderma lucidum</i> hydrolysate. Food Biotechnology, 34(4), 338-351.
42. Cristina Torres-Fuentes, María del Mar Contreras, Isidra Recio, Manuel Alaiz, Javier Vioque,Identification and characterization of antioxidant peptides from chickpea protein hydrolysates,Food Chemistry,Volume 180,**2015**,Pages 194-202,ISSN 0308-8146.

.
