# Supplementary figures and images for: Physicochemical Properties and Stability of Antioxidant Peptides from Swim Bladder of Grass Carp (Ctenopharyngodon idella)
Source: Foods. 2025 Mar 30;14(7):1216. doi: 10.3390/foods14071216 (PMC12512379; doi:10.3390/foods14071216)

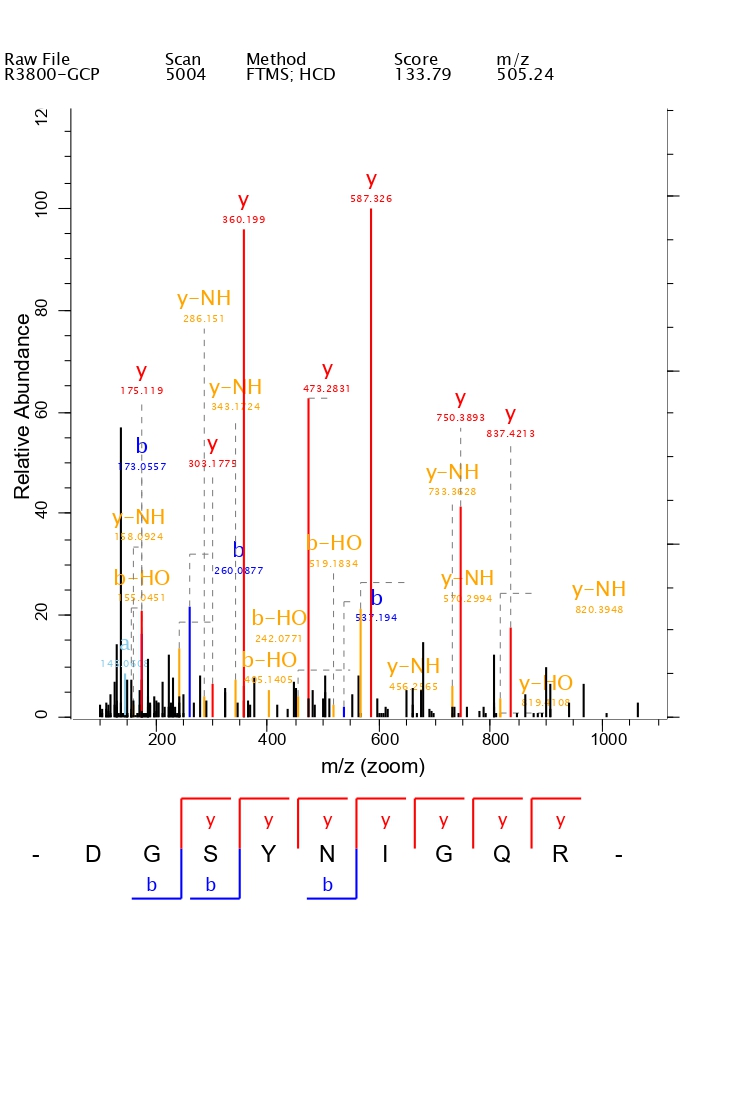

Supplement: Supplementary file 1 [file foods-14-01216-s001.zip › DGSYNIGQR.jpg]

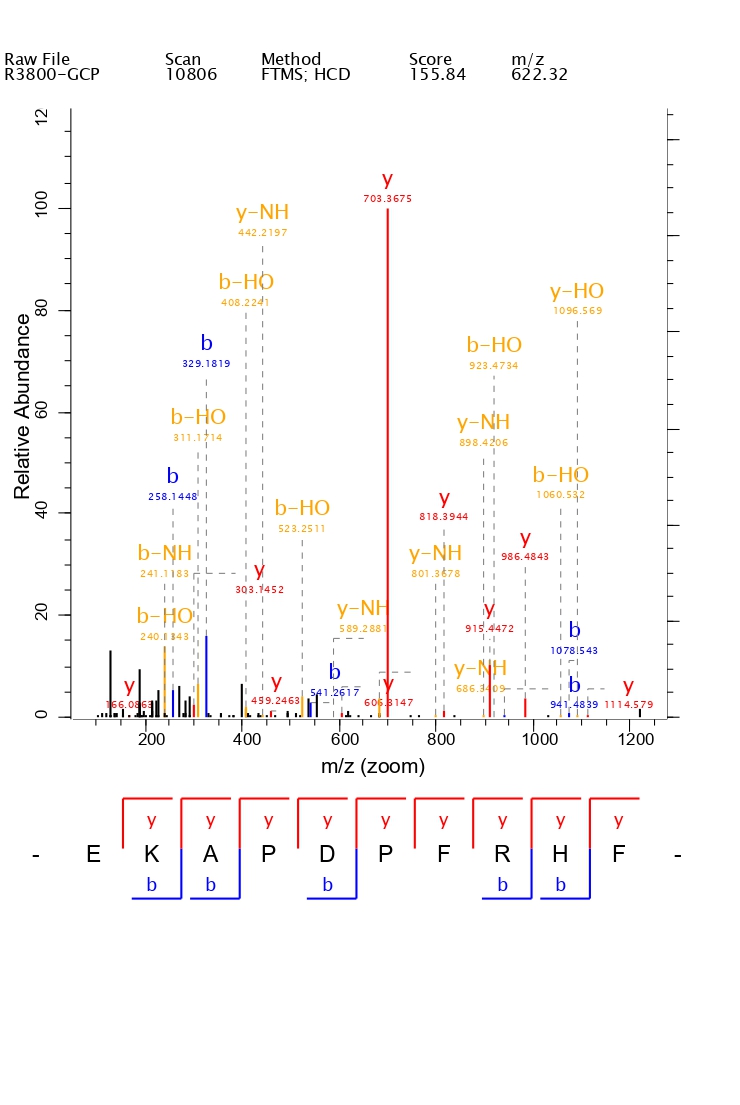

Supplement: Supplementary file 1 [file foods-14-01216-s001.zip › EKAPDPFRHF.jpg]

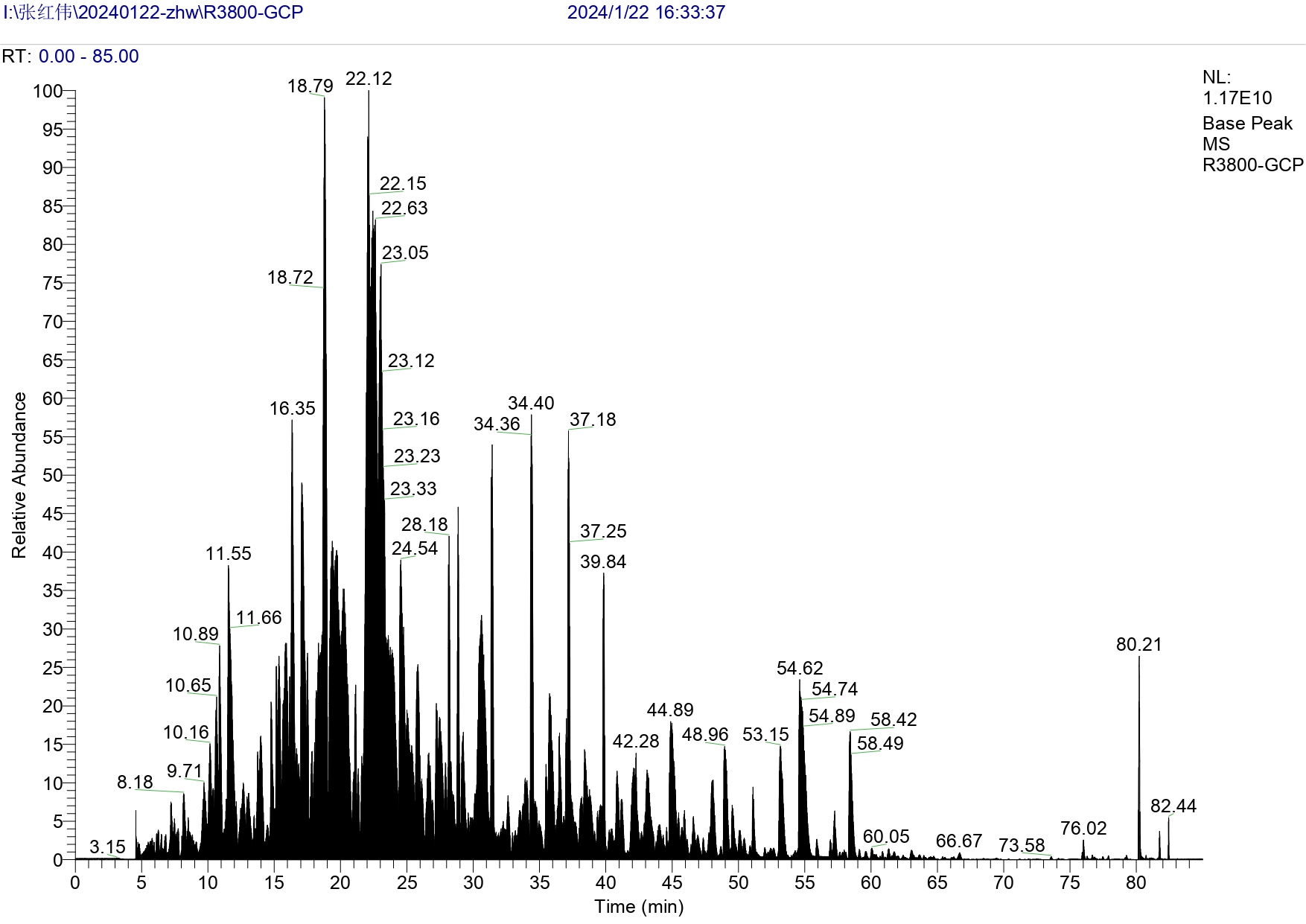

Supplement: Supplementary file 1 [file foods-14-01216-s001.zip › GCP_BASE PEAK_page-0001.jpg]

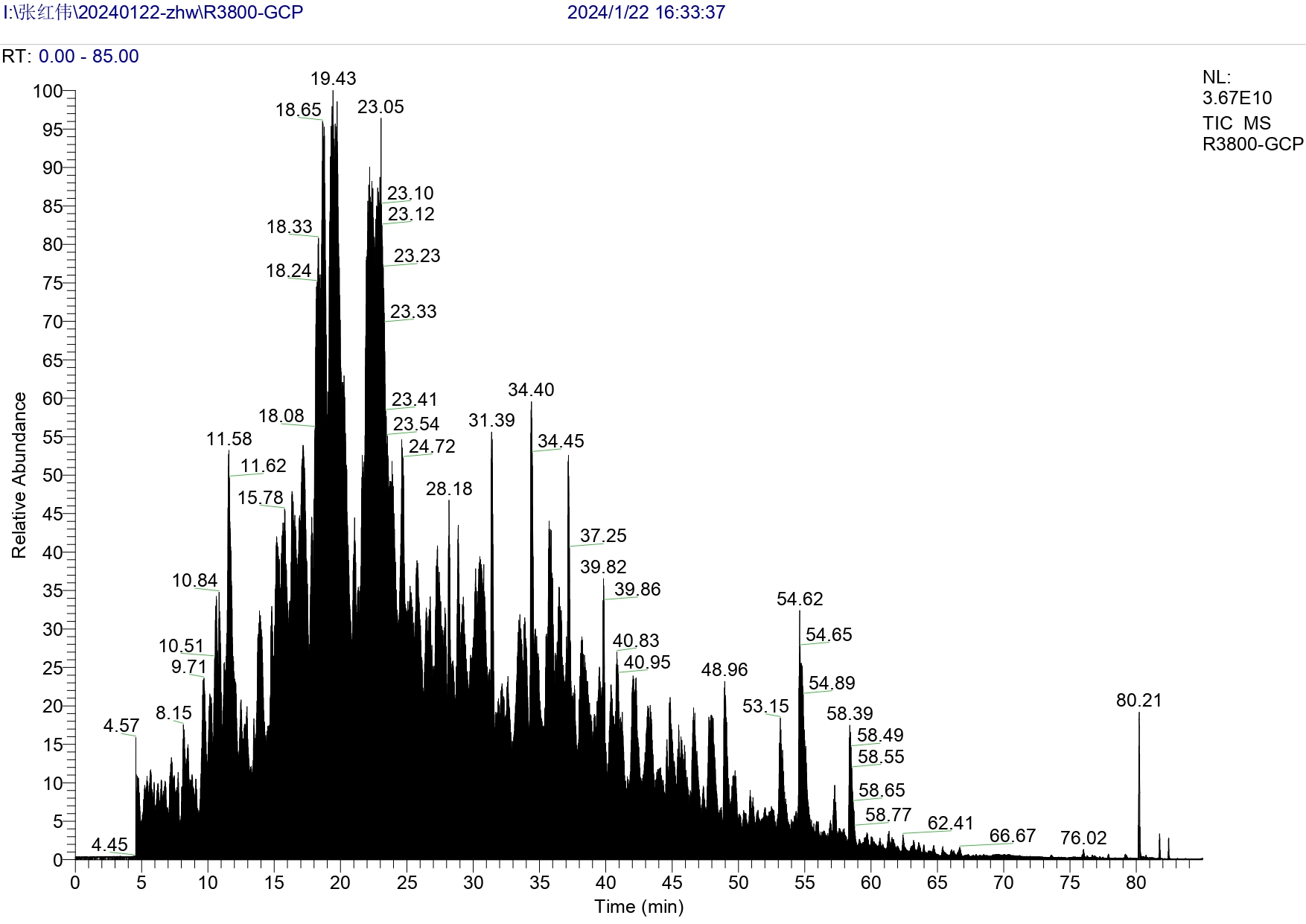

Supplement: Supplementary file 1 [file foods-14-01216-s001.zip › GCP_TIC_page-0001.jpg]

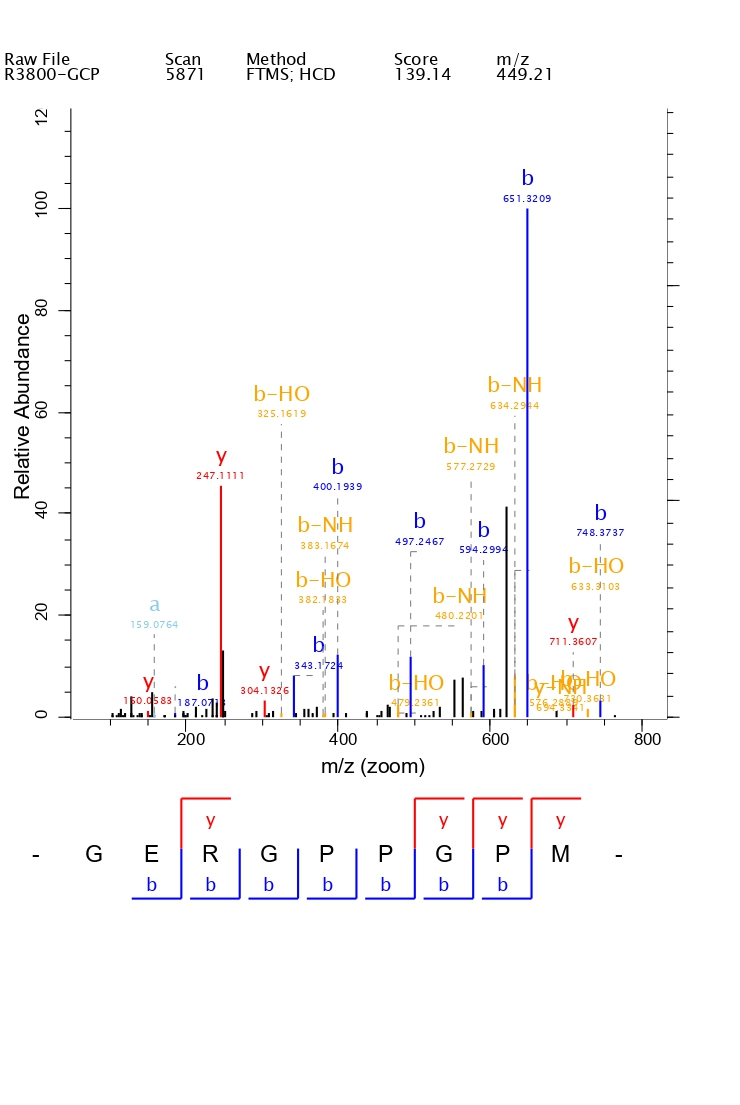

Supplement: Supplementary file 1 [file foods-14-01216-s001.zip › GERGPPGPM.jpg]

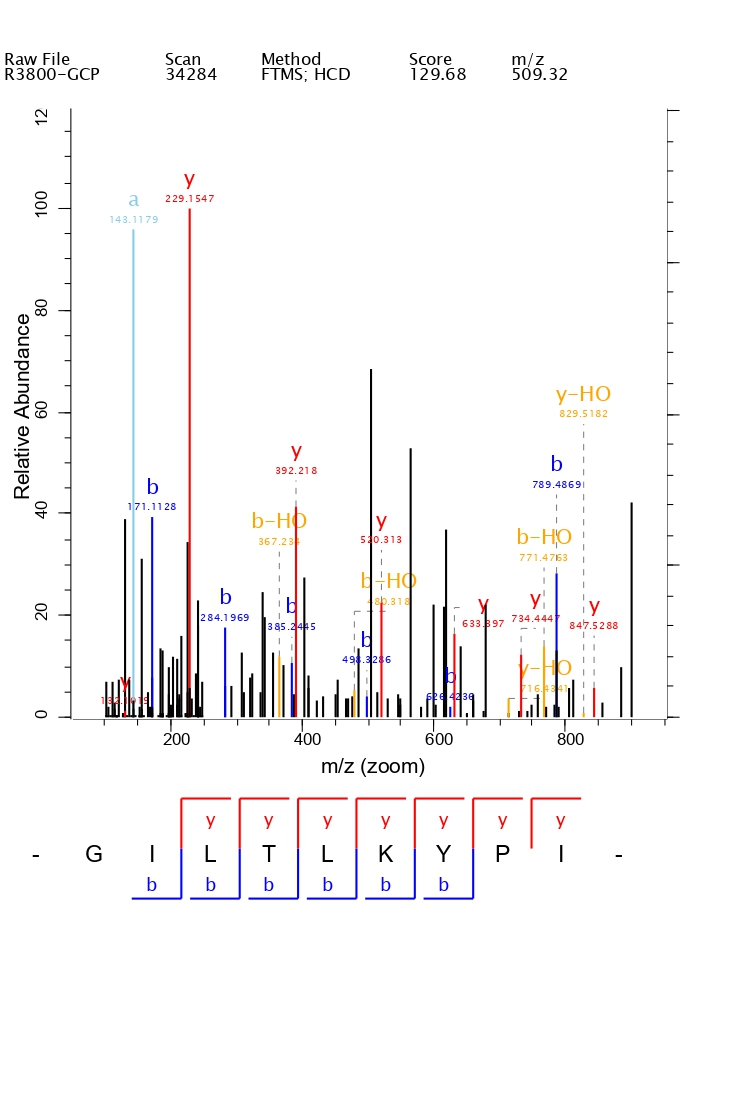

Supplement: Supplementary file 1 [file foods-14-01216-s001.zip › GILTLKYPI.jpg]

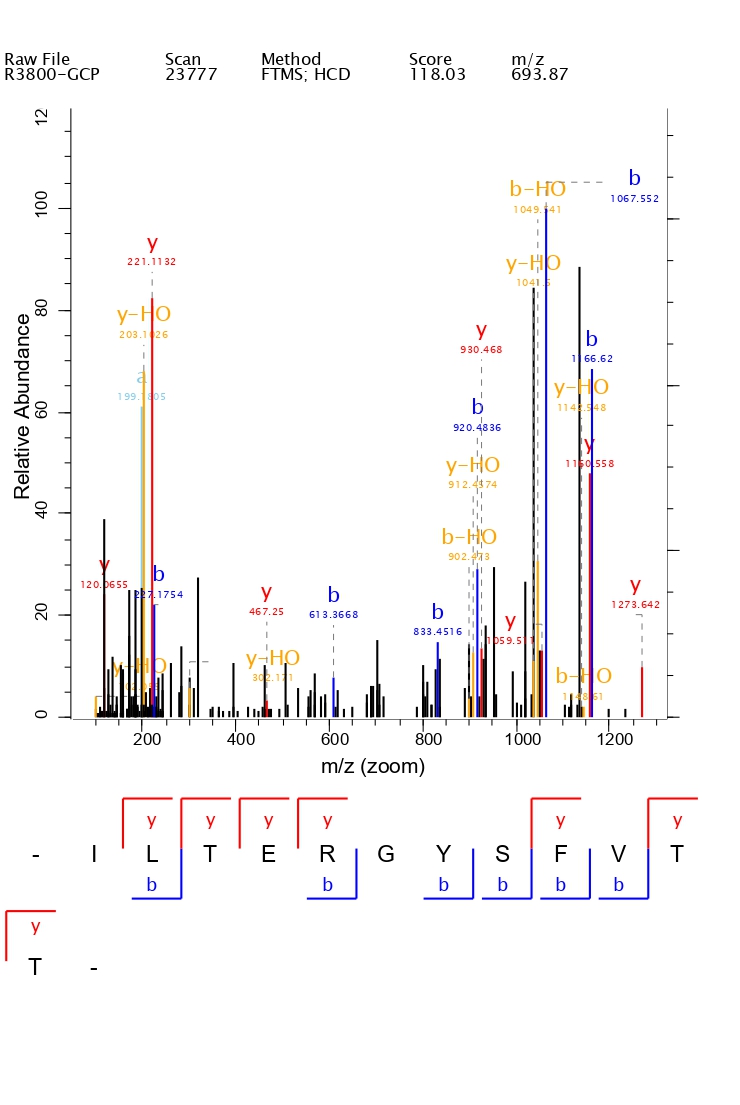

Supplement: Supplementary file 1 [file foods-14-01216-s001.zip › ILTERGYSFVTT.jpg]

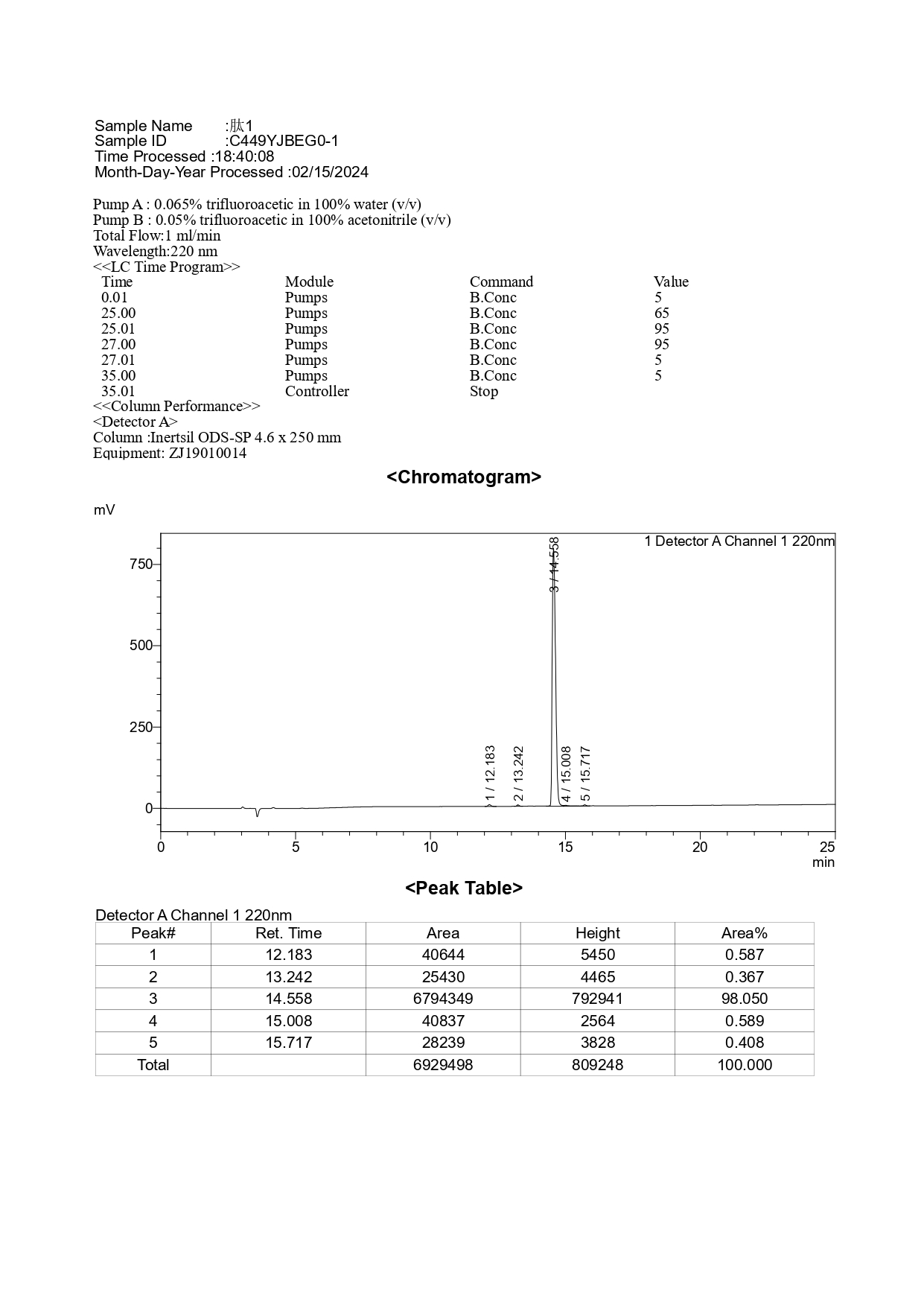

Supplement: Supplementary file 1 [file foods-14-01216-s001.zip › peptide-1-HPLC.jpg]

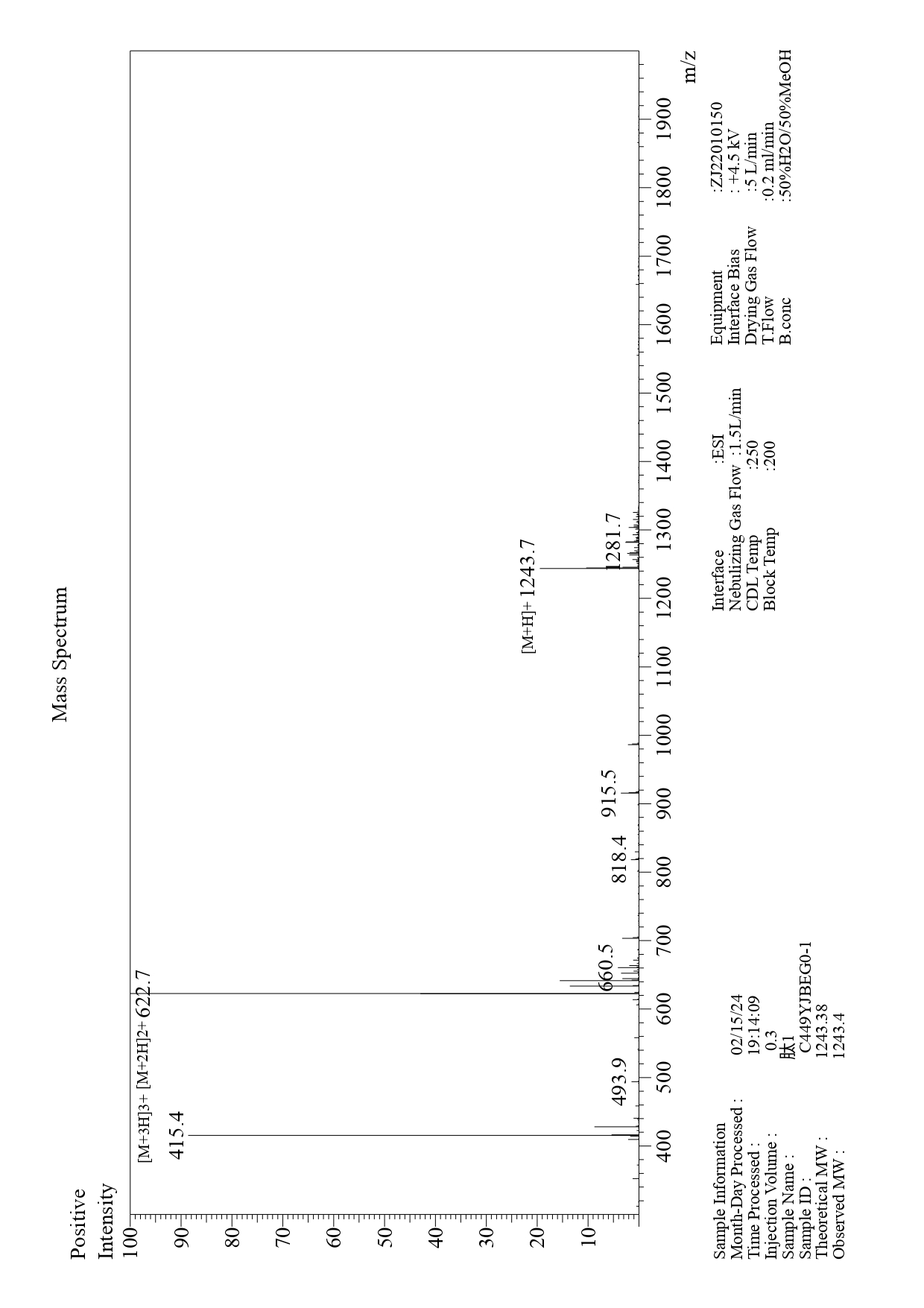

Supplement: Supplementary file 1 [file foods-14-01216-s001.zip › peptide-1-MS.jpg]

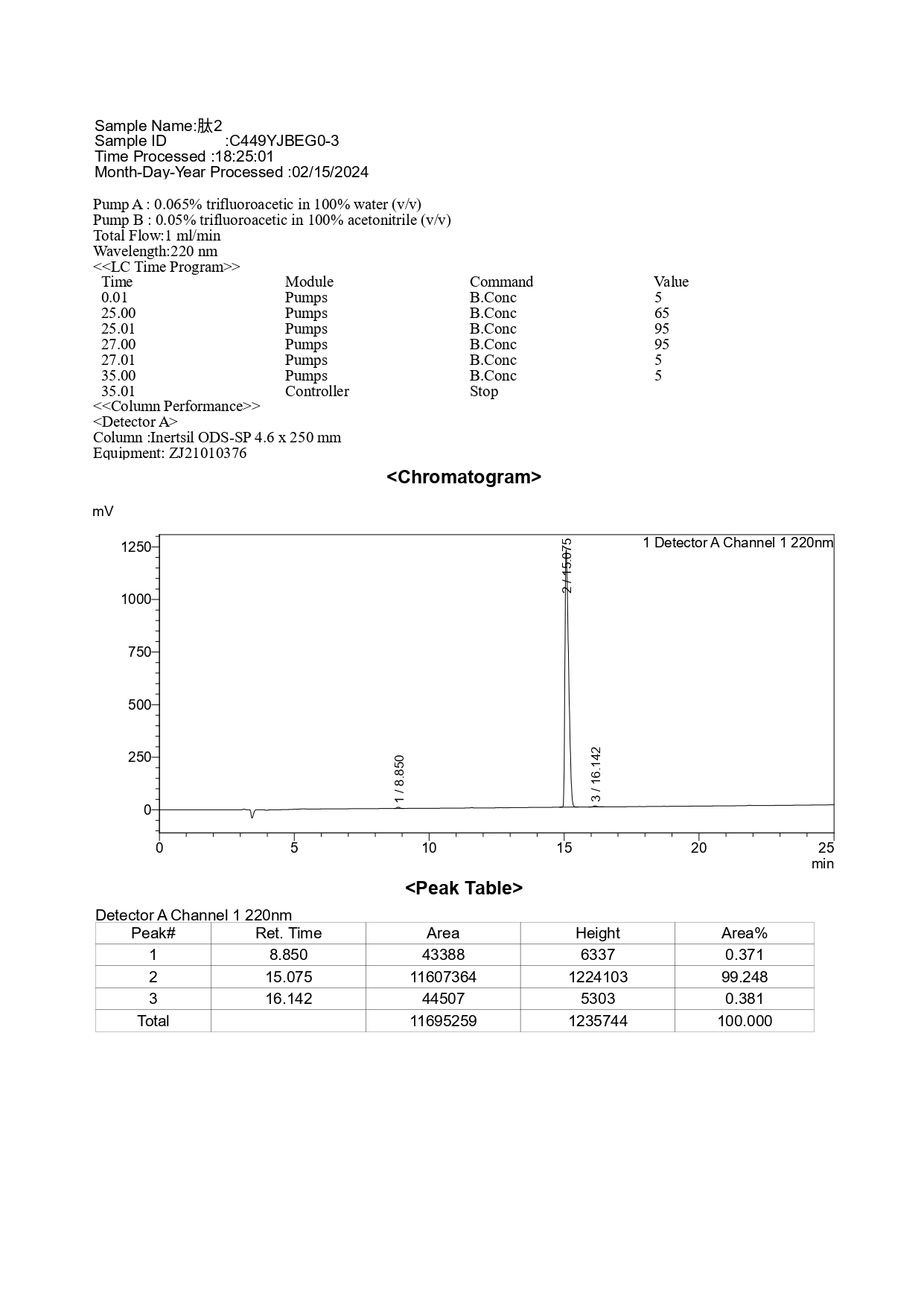

Supplement: Supplementary file 1 [file foods-14-01216-s001.zip › peptide-2-HPLC.jpg]

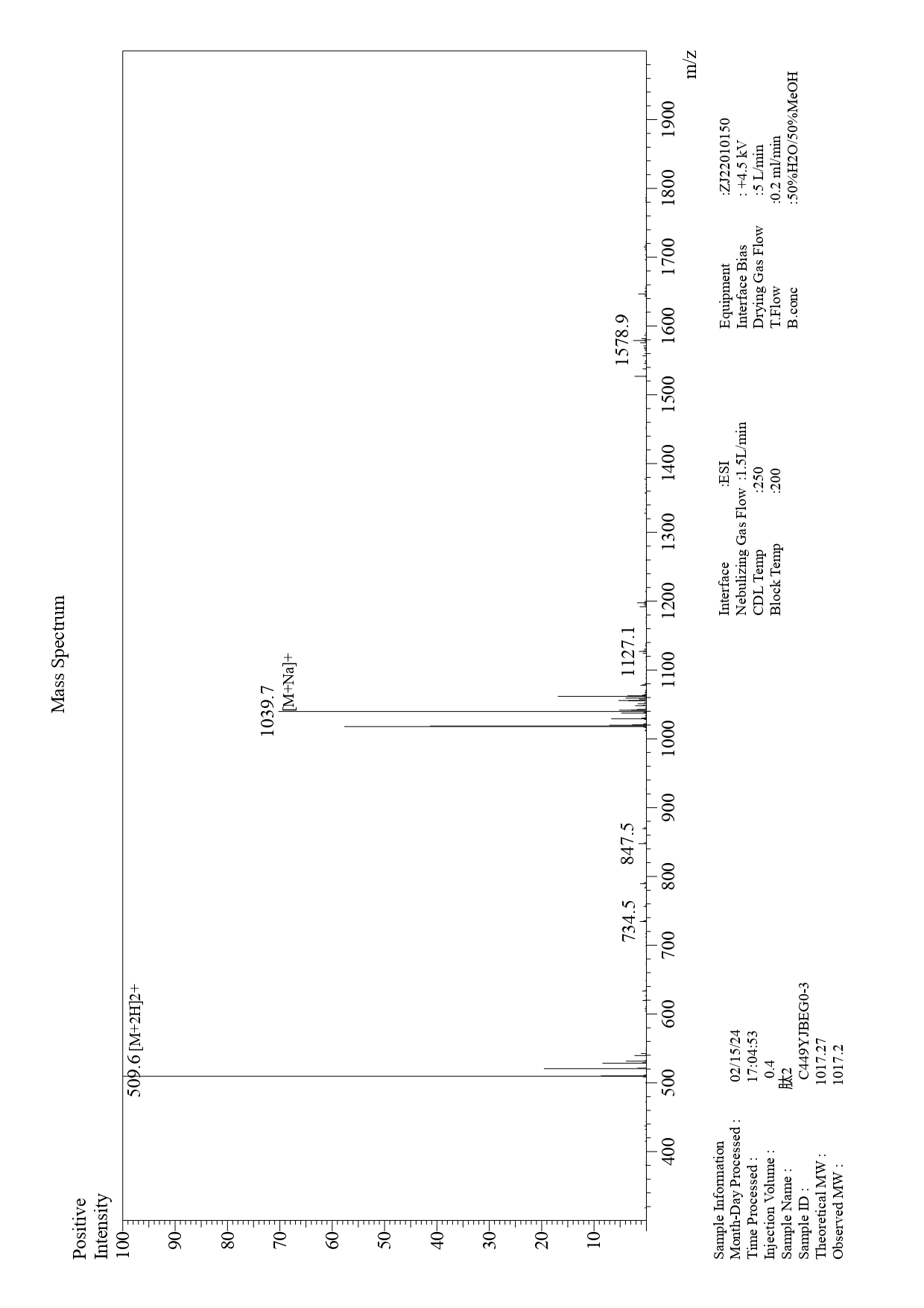

Supplement: Supplementary file 1 [file foods-14-01216-s001.zip › peptide-2-MS.jpg]

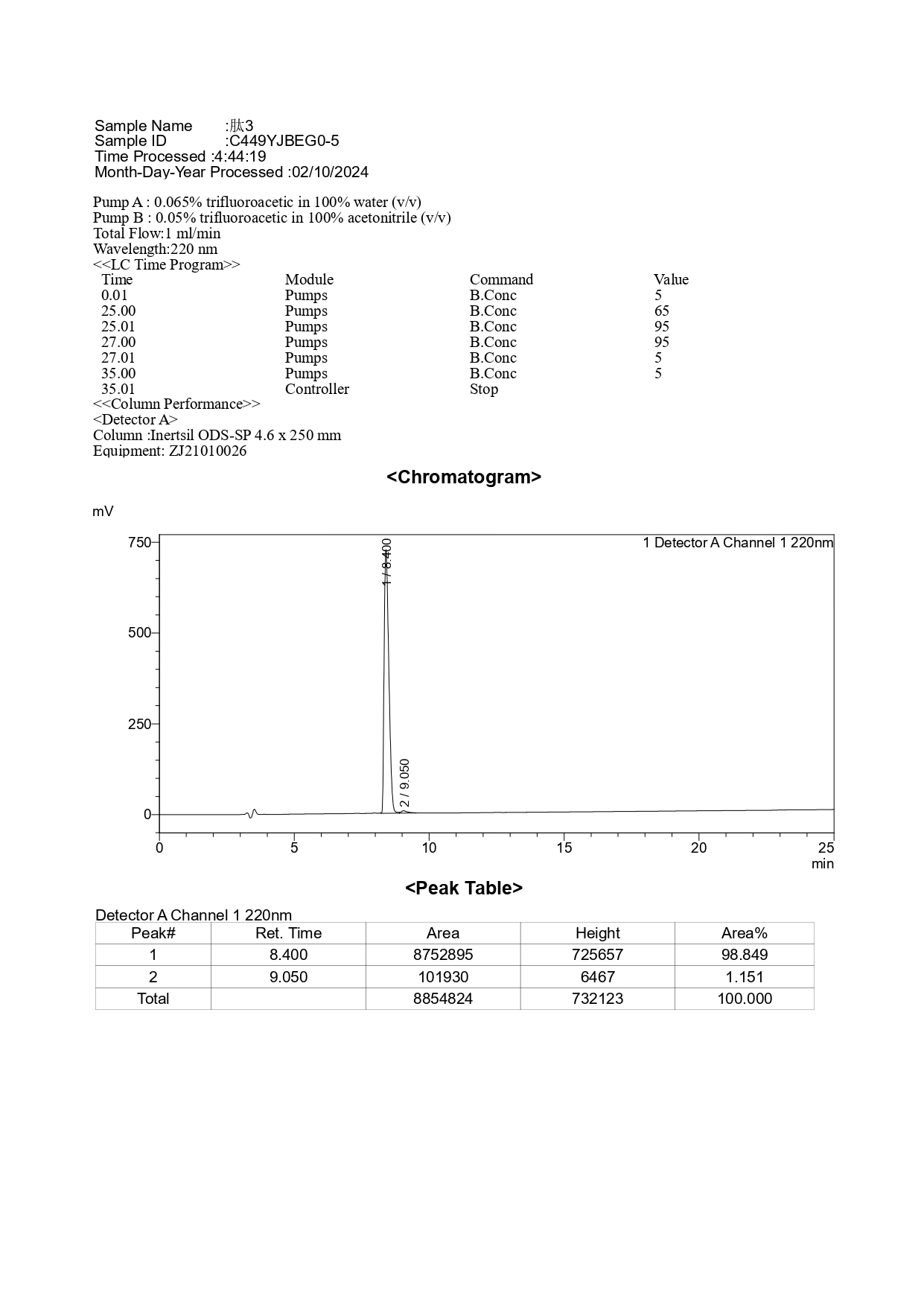

Supplement: Supplementary file 1 [file foods-14-01216-s001.zip › peptide-3-HPLC.jpg]

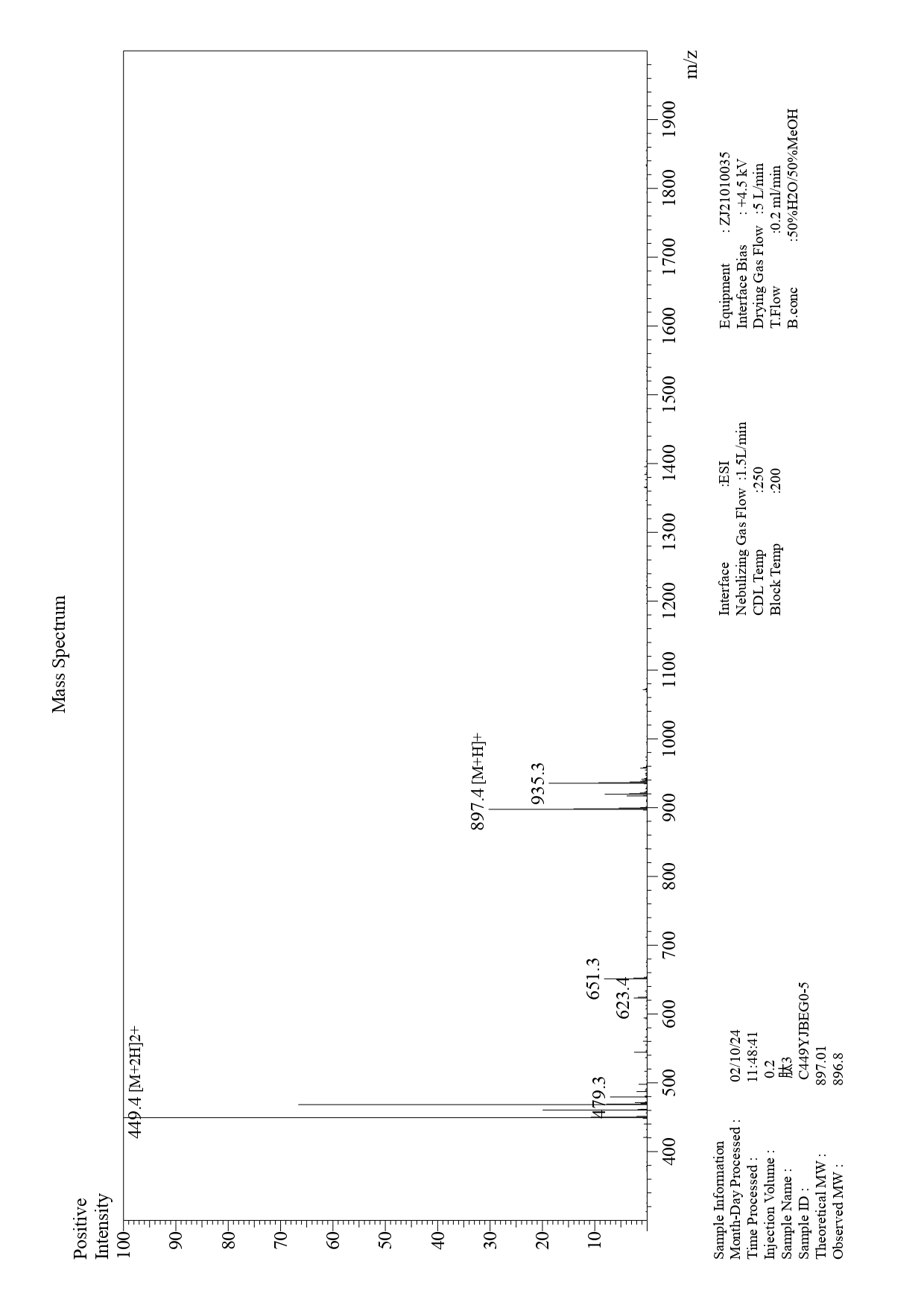

Supplement: Supplementary file 1 [file foods-14-01216-s001.zip › peptide-3-MS.jpg]

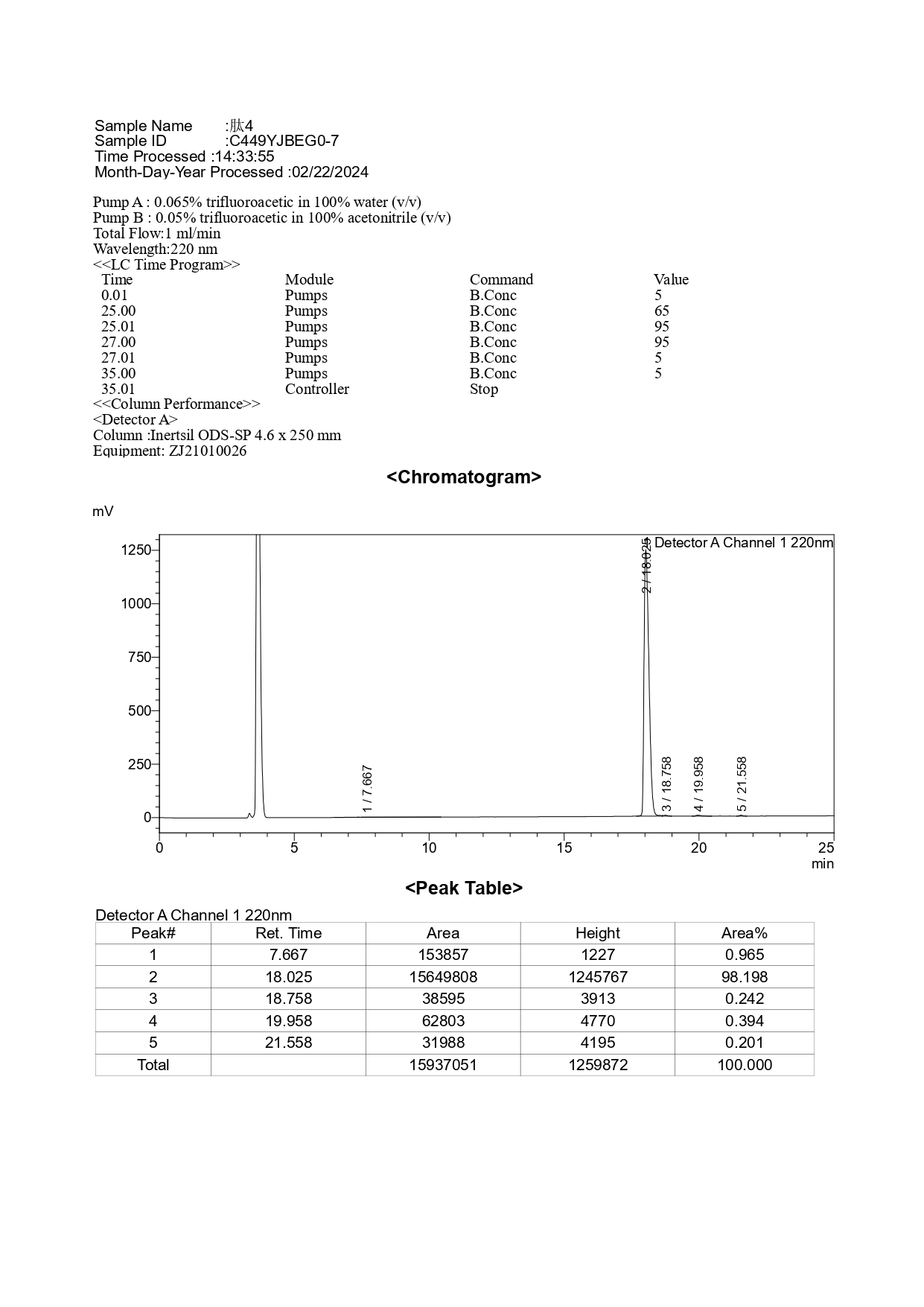

Supplement: Supplementary file 1 [file foods-14-01216-s001.zip › peptide-4-HPLC.jpg]

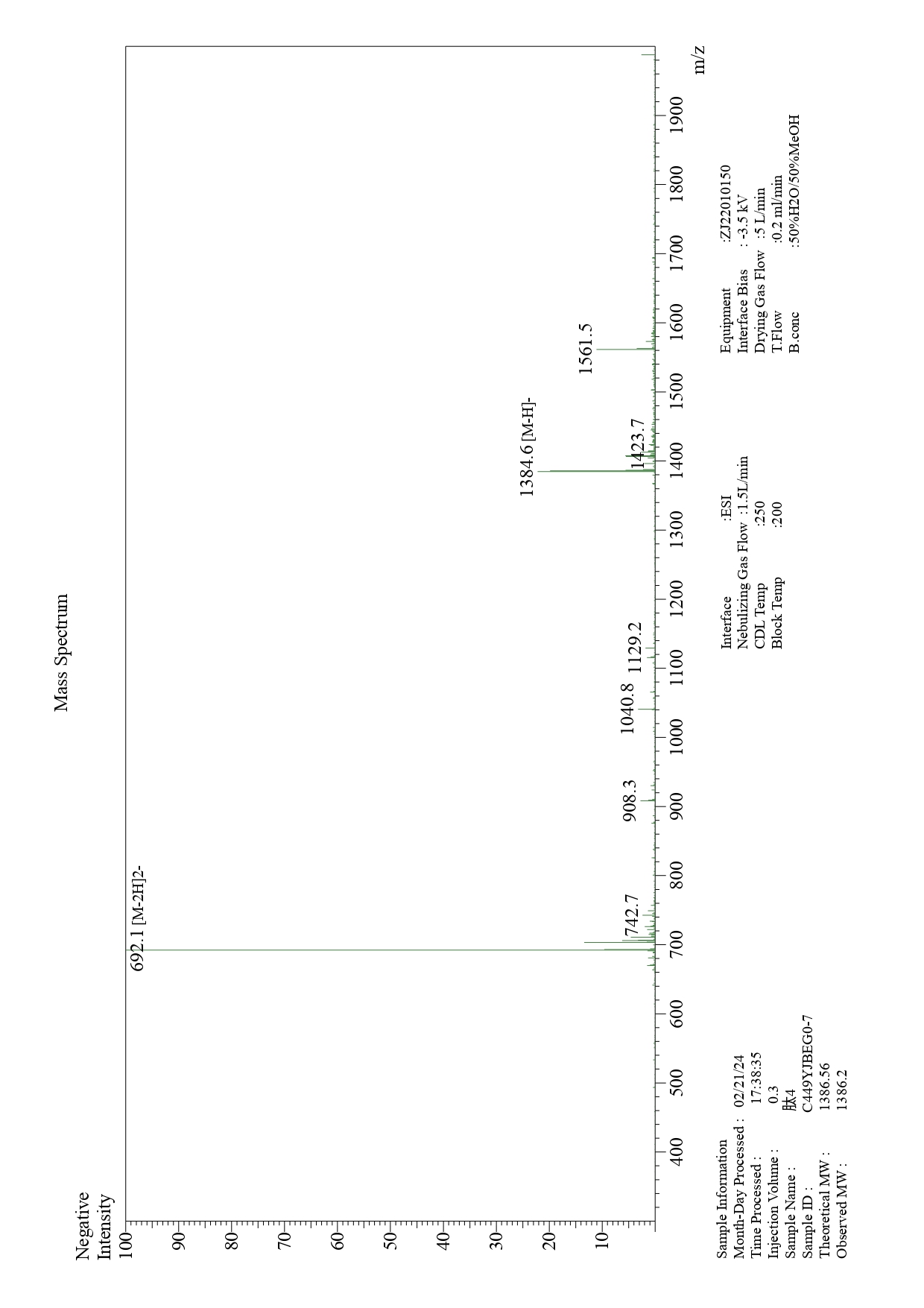

Supplement: Supplementary file 1 [file foods-14-01216-s001.zip › peptide-4-MS.jpg]

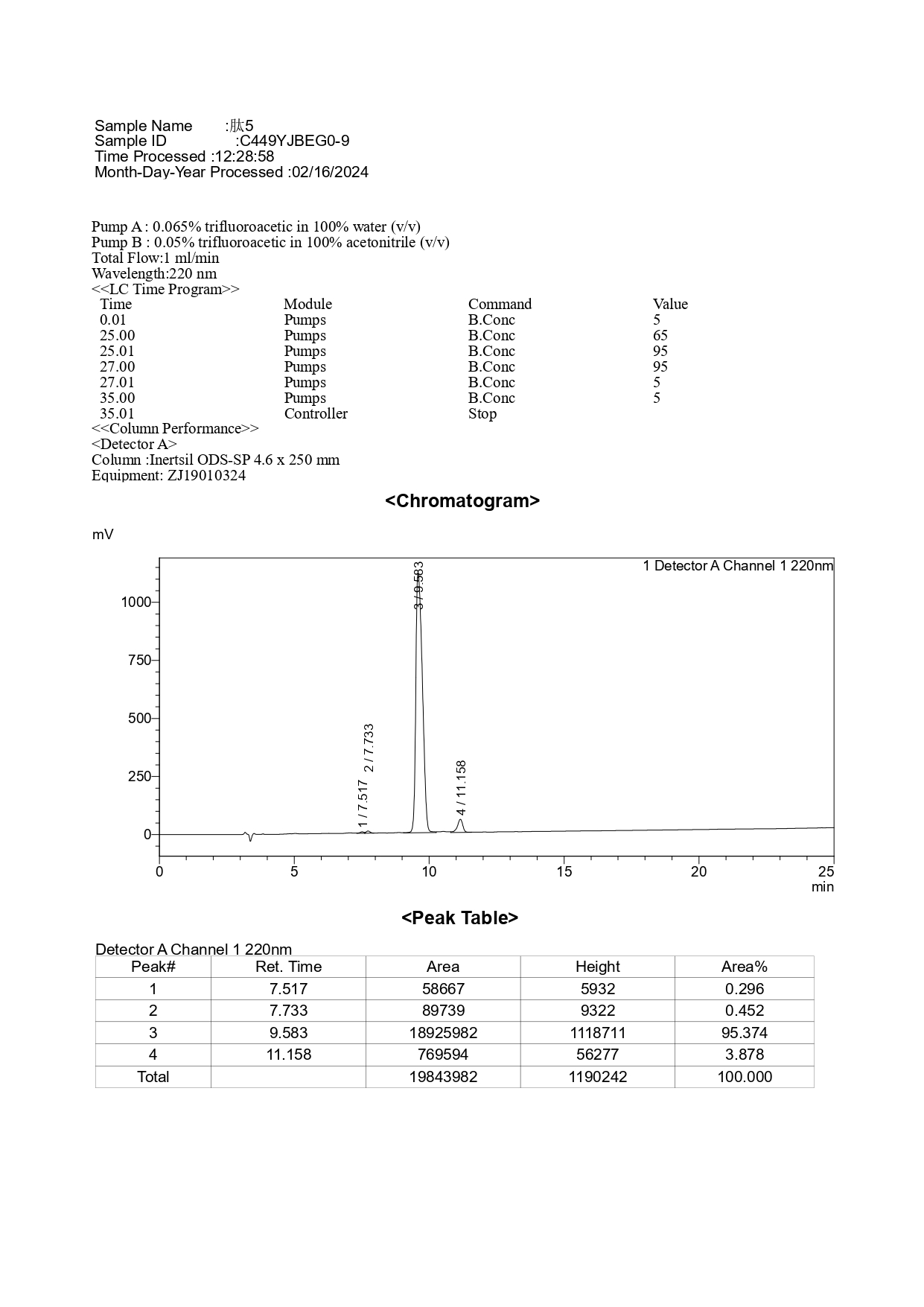

Supplement: Supplementary file 1 [file foods-14-01216-s001.zip › peptide-5-HPLC.jpg]

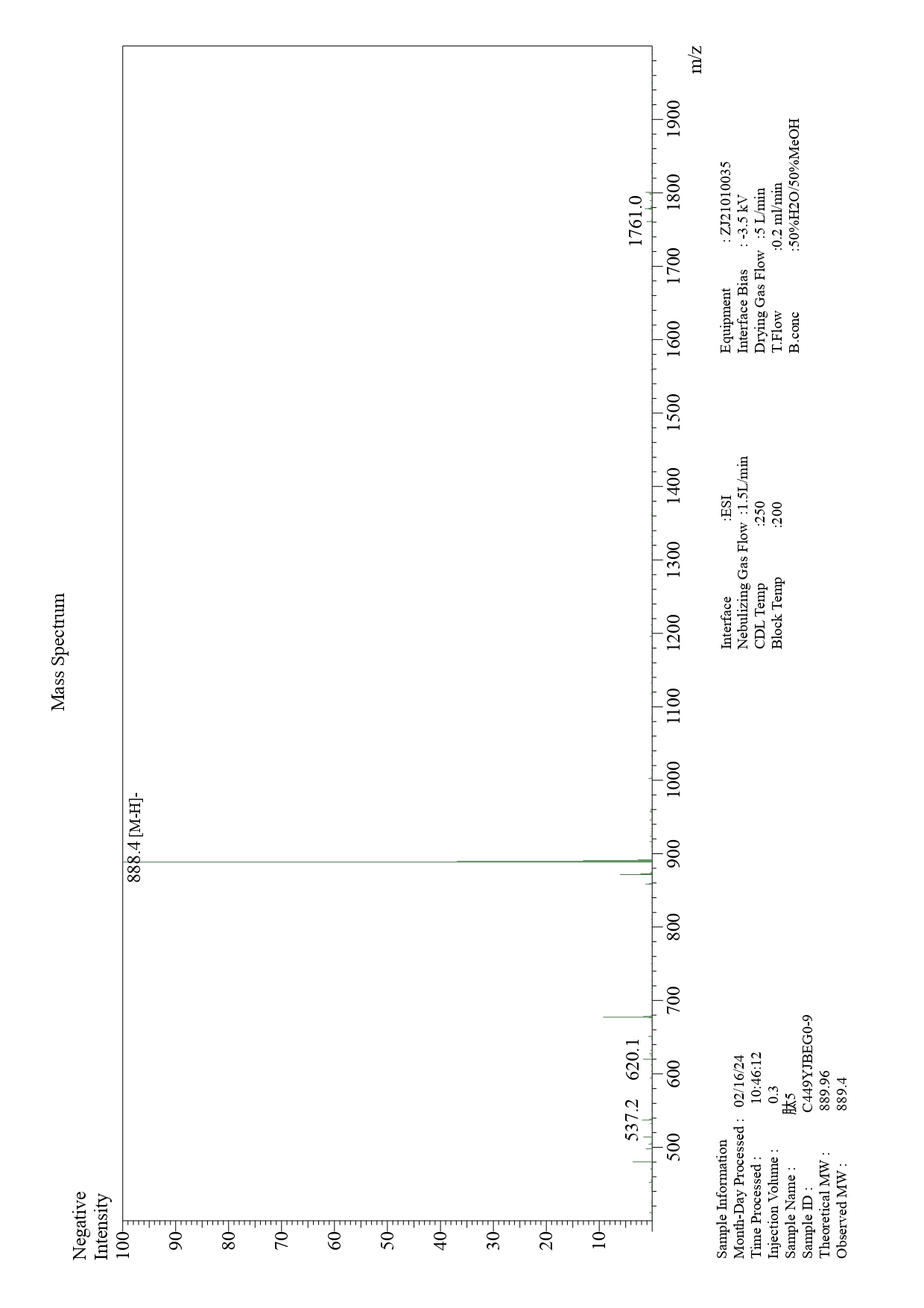

Supplement: Supplementary file 1 [file foods-14-01216-s001.zip › peptide-5-MS.jpg]

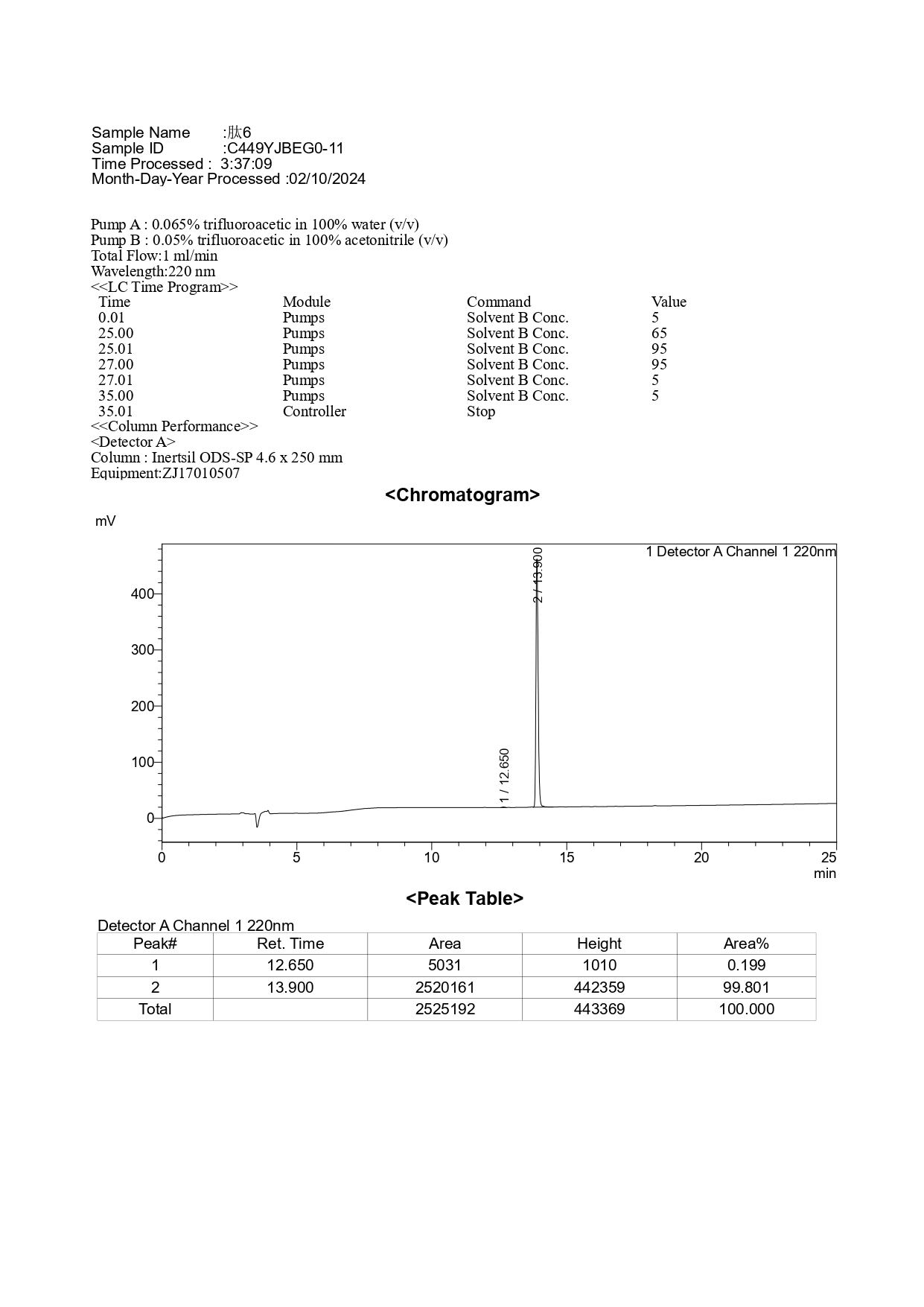

Supplement: Supplementary file 1 [file foods-14-01216-s001.zip › peptide-6-HPLC.jpg]

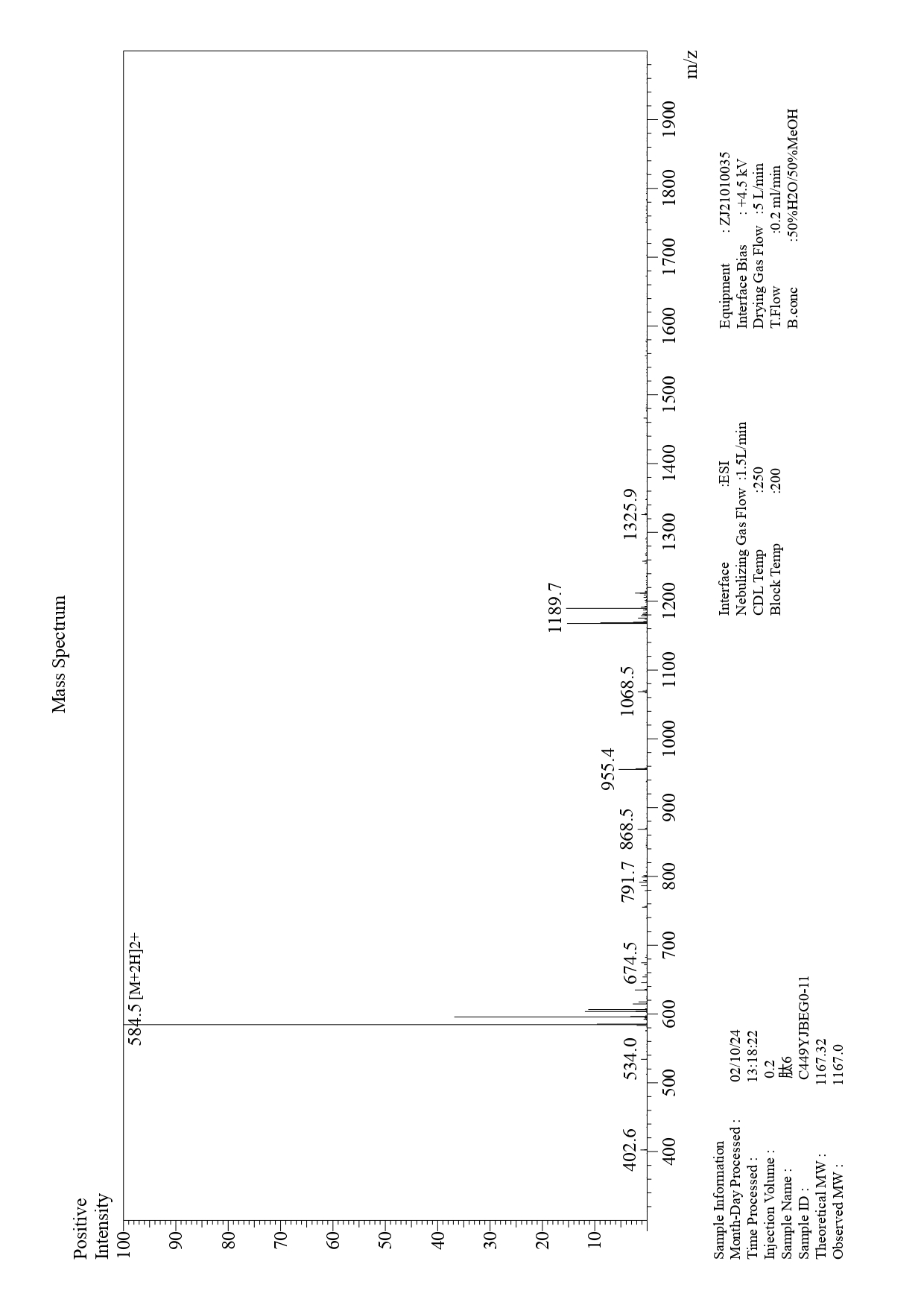

Supplement: Supplementary file 1 [file foods-14-01216-s001.zip › peptide-6-MS.jpg]

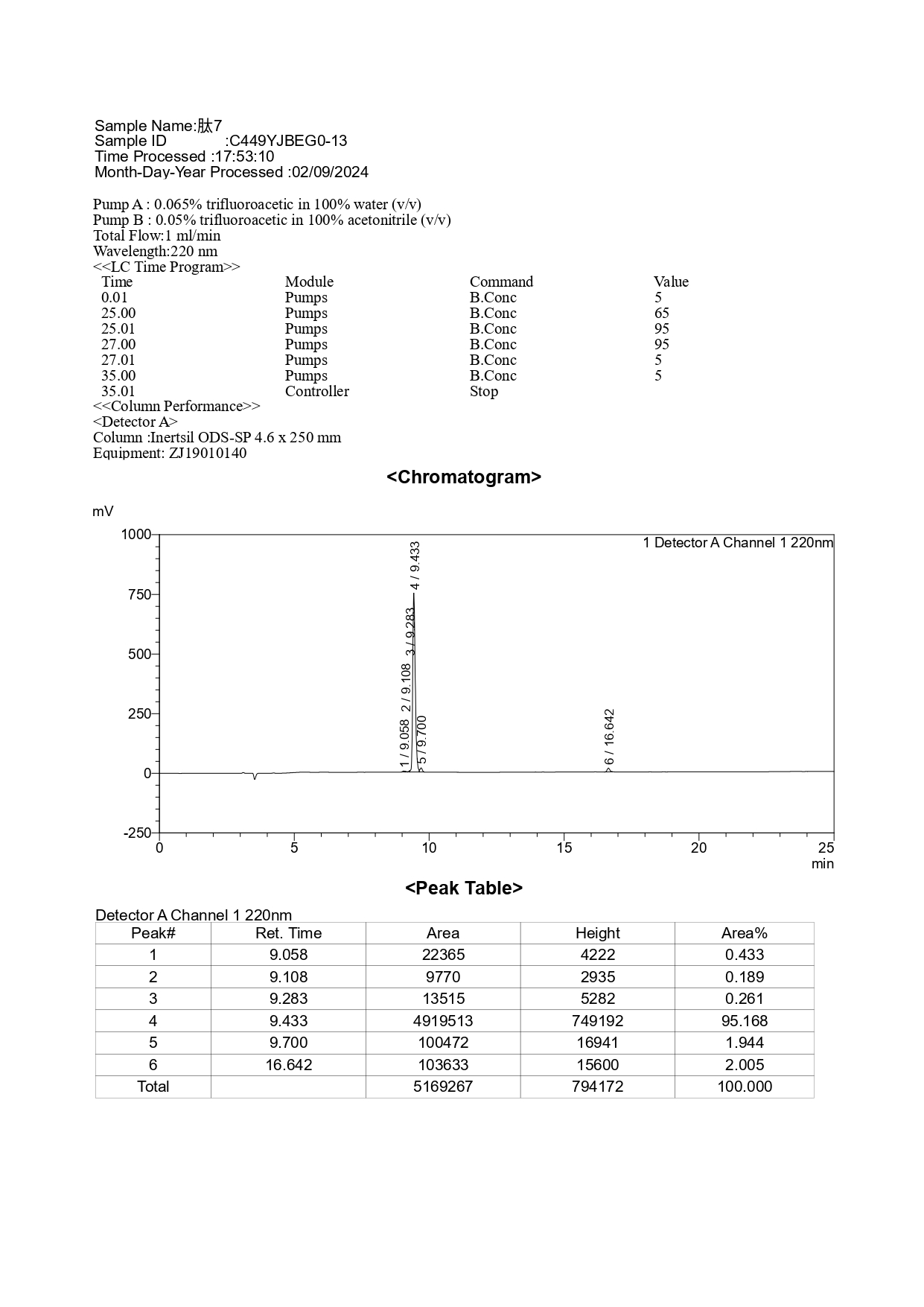

Supplement: Supplementary file 1 [file foods-14-01216-s001.zip › peptide-7-HPLC.jpg]

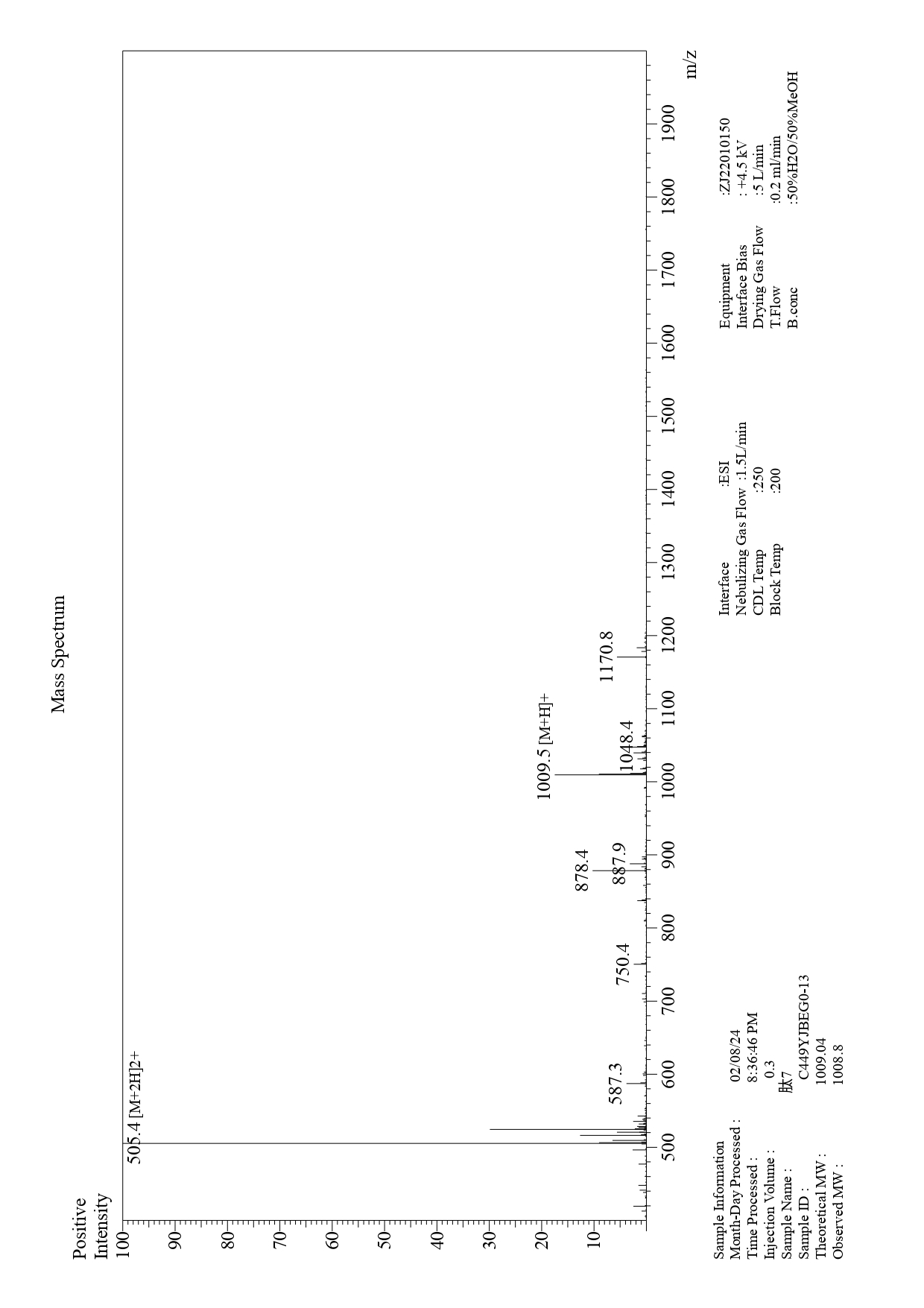

Supplement: Supplementary file 1 [file foods-14-01216-s001.zip › peptide-7-MS.jpg]

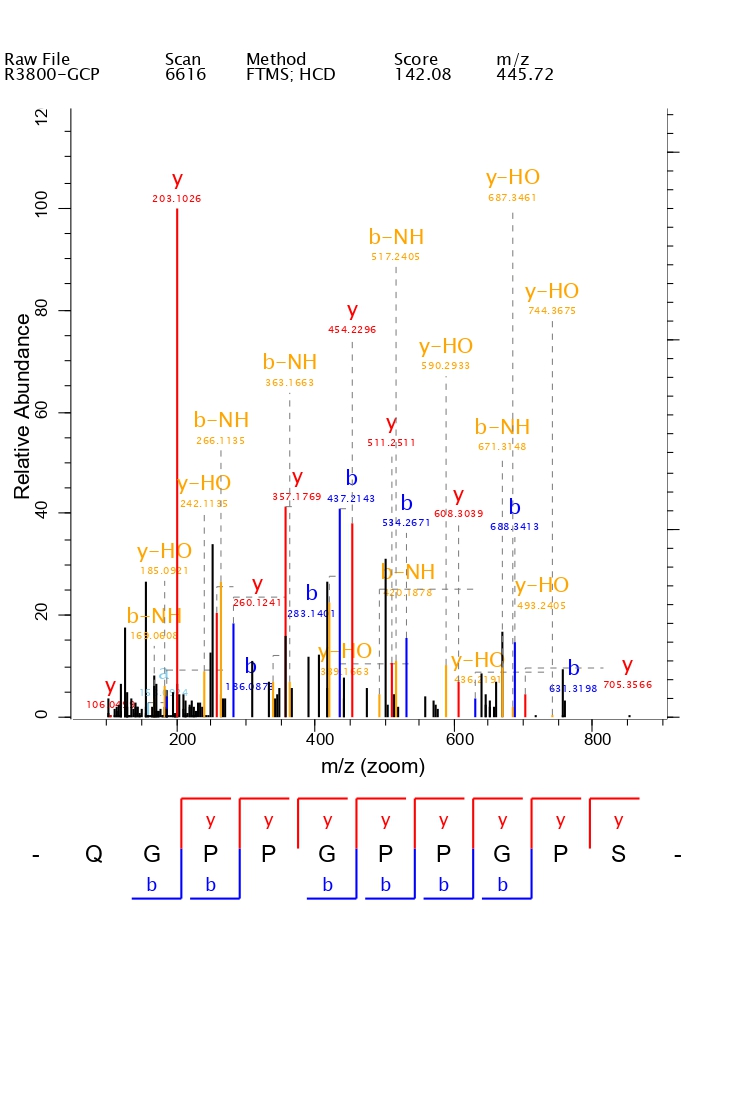

Supplement: Supplementary file 1 [file foods-14-01216-s001.zip › QGPPGPPGPS.jpg]

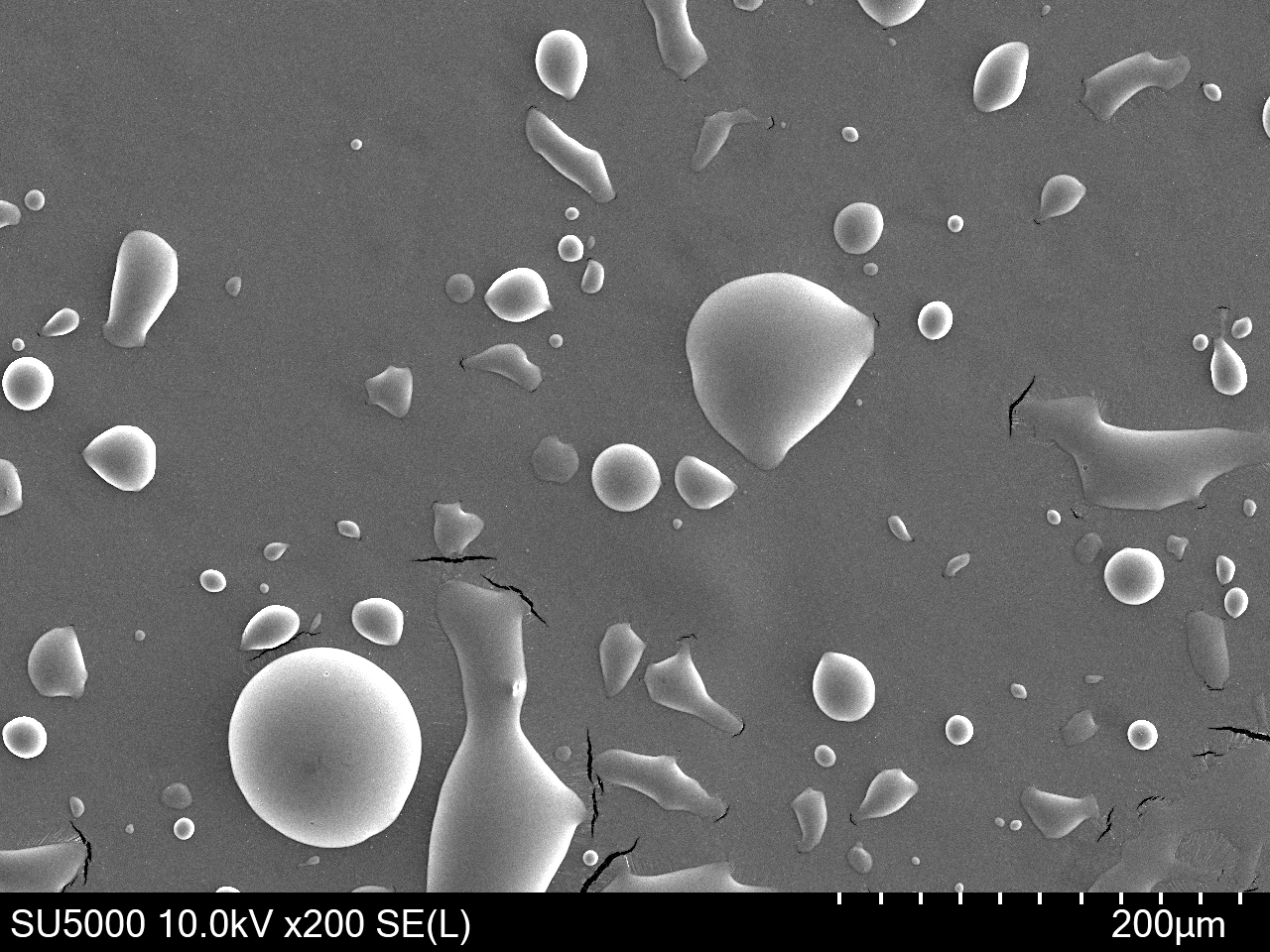

Supplement: Supplementary file 1 [file foods-14-01216-s001.zip › SEM.tif]

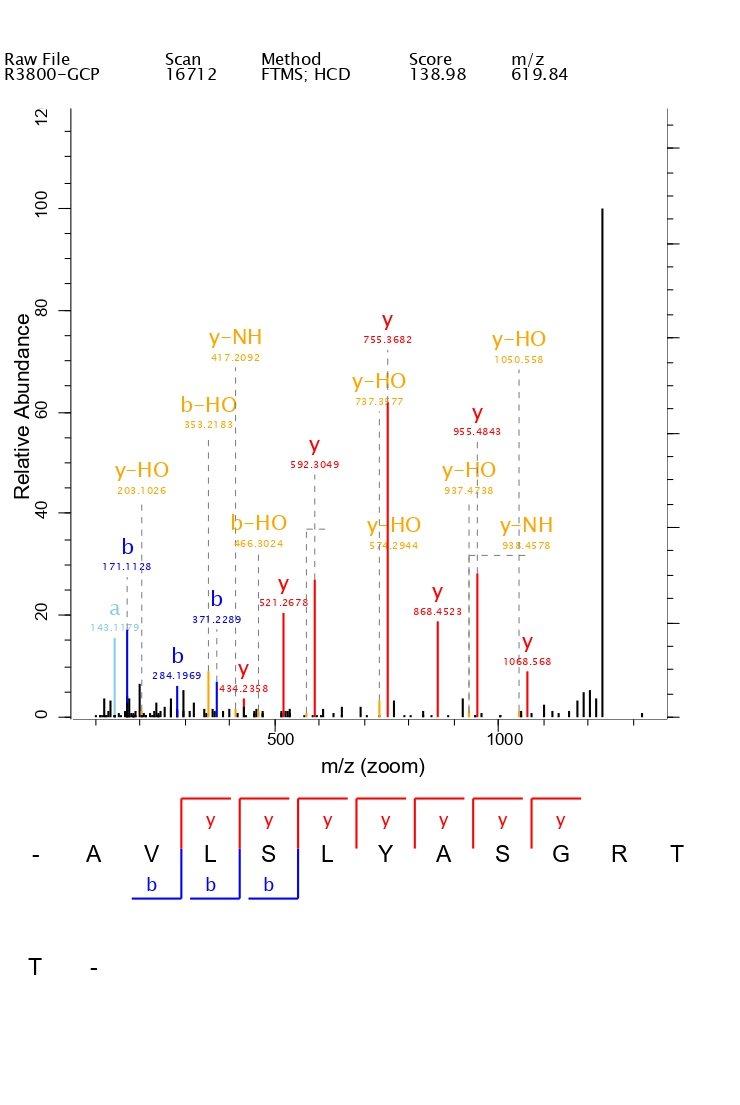

Supplement: Supplementary file 1 [file foods-14-01216-s001.zip › VLSLYASGRTT.jpg]
